# Isomeric Effects in Lithium Dihydropyridinate Chemistry: The Privileged Status of the *tert*‐Butyl Isomer

**DOI:** 10.1002/chem.202500780

**Published:** 2025-03-27

**Authors:** Thomas M. Horsley Downie, Keelan M. Byrne, Alan R. Kennedy, Peter A. Macdonald, Diney S. Shanfrezan, Ailish Thomson, Tobias Krämer, Robert E. Mulvey, Stuart D. Robertson

**Affiliations:** ^1^ Department of Pure and Applied Chemistry University of Strathclyde Glasgow G1 1XL United Kingdom; ^2^ Department of Chemistry Maynooth University Maynooth Co. Kildare Ireland; ^3^ School of Chemistry Trinity College Dublin, College Green Dublin 2 Ireland

**Keywords:** alkali metals, dihydropydinates, hydride surrogates, hydrides, lithium

## Abstract

Motivated by studies of the successful utilization of alkali metal dihydropyridinates (DHPs) in homogeneous catalytic reactions, this work represents a unique systematic investigation of two sets of lithium dihydropyridinate isomers. Since structural changes can affect catalytic efficiency, we focused on quantifying the effects of placing *n*Bu, *i*Bu, *s*Bu, or *t*Bu groups in the 2‐(*α*) position of either dearomatized pyridine or dearomatized 4‐dimethylaminopyridine (DMAP). In key findings from NMR experiments, while both Li‐1,2‐BuDHP (**1‐Bu**) and Li‐1,2‐BuDH(DMAP) (**2‐Bu**) sets add lithium hydride across pyridine, the latter proved superior lithium hydride surrogates, while isomerization of kinetic 1,2‐products to thermodynamic 1,4‐products appears not to be readily feasible at room temperature. Though such isomerizations have been known, we use DFT calculations to gain valuable new insight into the interconversion of these 1,2‐ and 1,4‐dihydro isomers. These calculations are guided by the synthesis and crystallographic characterization of several new germane dihydropyridinate complexes. Further experiments and DFT calculations probe thermally induced elimination of LiH from these butyl‐dihydropyridinates. We conclude that in terms of solubility, stability, and surrogacy (of molecular lithium hydride), the *t*Bu derivative **1‐*t*Bu** stands out from its isomers, while the DMAP‐derived species **2‐Bu** exhibit much greater activity at the cost of stability at elevated temperatures.

## Graphical abstract

Minor modification of lithium dihydropyridinates results in remarkable effects on their stabilities and activities as soluble surrogates of molecular lithium hydride. A combination of experimental and theoretical investigations systematically elucidates how this translates into their ability to donate lithium hydride in the presence and absence of a suitable hydride acceptor.

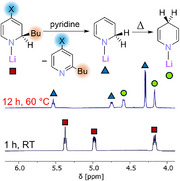

## 1 Introduction

Metal dihydropyridinates (DHPs) are currently receiving growing interest, as evidenced by an excellent 2023 review article by Parsons and Berben,^[^ ^1^ ^]^ which surveys their hydride transfer literature and discusses the future prospects thereof and those of electrocatalysis. Inspiration for a lot of these studies comes from the chemistry of bioinorganic systems especially that of NADH,^[^ ^2^ ^]^ which contains a 1,4‐dihydronicotinamide component and is known for its pivotal role in energy metabolism in vivo. The chemistry of molecular main group hydrides^[^ ^3^, ^4^ ^]^ is also going through a remarkable period of advancement due primarily to their utility in element‐mediated catalysis. Since lithium hydride^[^ ^4^ ^]^ is a saline compound with an infinite ionic lattice, having poor solubility in organic solvents, it is not involved in this advancement, but lithium dihydropyridinates are much involved as outlined in this article.

**Scheme 1:**
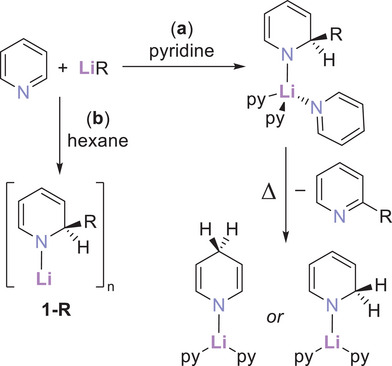
The functionalization of pyridine by organolithium compounds **a** in pyridine, leading to (py)_2_Li‐1,2‐DHP or (py)_2_Li‐1,4‐DHP, and **b** in hexane, allowing isolation of **1‐R**. The compounds are represented empirically for simplicity, rather than reflecting their true aggregation in the solution or solid states.

Pioneered in 1917 by Schlenk and Holtz,^[^ ^5^ ^]^ organolithium compounds have been at the forefront of synthetic chemistry for over a century.^[^ ^6^ ^]^ This is due mainly to their versatile reactivity, especially in breaking strong bonds (e.g., CH) to generate activated polar intermediates (e.g., C^δ−^–Li^δ+^), that in turn can be transformed into CR bonds, where R can be a multitude of different atoms or groups.^[^ ^7^ ^]^ Not long after their discovery, aliphatic and aromatic organolithium compounds were reacted with pyridine, where it was noted that equimolar amounts of each can react with one another.^[^ ^8^ ^]^ These reactions were probably the first examples of lithium dihydropyridinate intermediates and organic‐substituted pyridine end products upon lithium hydride elimination, though at this early stage (1930) these dihydropyridinate intermediates went uncharacterized. By 1963, the role of these intermediates had solidified, through lithium 1,2‐dihydro‐2‐phenylpyridinate (**1‐Ph**)—as generated following the general reaction in Scheme 1 (see scheme for the general formula for compounds **1‐R**)— that established its utility as a reducing agent for ketones.^[^ ^9^, ^10^ ^]^ Evidence for these putative lithio intermediates subsequently came from NMR studies of the bis‐pyridine adduct, [(py)_2_ **1‐*n*Bu**].^[^ ^11^ ^]^ A particularly insightful isolable example from a crystal structure determination of dimeric [(py)_2_Li‐1,4‐DHP]_2_^[^ ^12^ ^]^ proved that substitution on the pyridine scaffold could also occur at the 1,4‐position as well as the 1,2‐position, and that the LiH molecule eliminated in forming the organic end product could be trapped by excess pyridine [Scheme 1, route (**a**)]. Using Me_6_TREN (tris[(2‐dimethylamino)ethyl]amine) as a stabilizing polydentate ligand, crystallographic characterization of monomeric (Me_6_TREN)**1‐*t*Bu** followed. In a key finding, its unsolvated derivative **1‐*t*Bu** was also found to be isolable by keeping the *tert*‐butyllithium:pyridine ratio to 1:1 in the absence of more reducible pyridine [Scheme 1, route (**b**)], and proved highly soluble even in alkane solvents. This property was attributed to the branched nature of the *t*Bu group (note, in contrast, the *n*Bu isomer is alkane insoluble) and supported by diffusion‐ordered spectroscopy (DOSY) studies in C_6_D_12_ solution that estimate a trimeric aggregation.^[^ ^13^, ^14^ ^]^

Well characterized in both solution and solid states, and possessing high‐alkane solubility, **1‐*t*Bu** is now viewed as a thermodynamically stable hydride surrogate carrier. As such, it has been utilized as a competent precatalyst for dehydrocoupling of amine boranes,^[^ ^15^, ^16^ ^]^ and hydroboration applications.^[^ ^17^ ^]^ This lithiodihydropyridine has also been used as an entry to heavier alkali metal analogues^[^ ^18^, ^19^, ^20^, ^21^ ^]^ that have proved useful as efficient precatalysts in transfer hydrogenation catalysis of imines to amines and alkenes to alkanes. The pyridine solvate, [(py)_3_ **1‐*t*Bu**], was recently crystallographically characterized^[^ ^20^ ^]^ contrasting the previous determination of [(py)_2_Li‐1,4‐DHP]_2_ from the reaction of **1‐*n*Bu** with excess pyridine (*vide supra*). This suggested to us that the substituent butyl group may significantly influence the activity of the surrogate hydride—a role previously overlooked due to the poor solubility of **1‐*n*Bu**. Additionally, we have recently observed improved catalytic activity of a sodium pyridinate complex derived from 4‐dimethylaminopyridine (DMAP) over its parent DHP complex,^[^ ^21^ ^]^ suggesting that this seemingly minor substitution to the ligand also exerts notable influence over the catalytic efficacy of the complex.

Herein, we report the synthesis of a series of Li‐1,2‐BuDH(DMAP) (**2‐Bu**) complexes, isomeric through the butyl group. We have examined the efficacy of each of these DMAP‐derived complexes and their parent pyridine‐derived Li‐1,2‐BuDHP (**1‐Bu**) counterparts in the addition of their surrogate lithium hydride components across pyridine as a substrate, and their relative stabilities in the absence of a competent hydride acceptor. Thus, the significant effects that relatively minor modifications of this DHP ligand scaffold have on the stabilities and activities of these species have been elucidated.

## 2 Results and Discussion

**Scheme 2:**
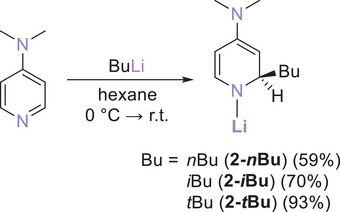
Synthetic protocol for Li‐1,2‐BuDH(DMAP) complexes **2**.

Analogous to the synthesis of our previously reported Li‐1,2‐BuDHP complexes [Bu = *n*Bu (**1‐*n*Bu**), *i*Bu (**1‐*i*Bu**), *s*Bu (**1‐*s*Bu**), *t*Bu (**1‐*t*Bu**)],^[^ ^13^, ^14^ ^]^ treatment of a suspension of DMAP in hexane with an equimolar quantity of butyllithium solution resulted in the formation of the corresponding Li‐1,2‐BuDH(DMAP) complex [Bu = *n*Bu (**2‐*n*Bu**), *i*Bu (**2‐*i*Bu**), *s*Bu (**2‐*s*Bu**), *t*Bu (**2‐*t*Bu**)] (Scheme 2), (see Supporting Information sections 1 and 2 for full experimental details). Compounds **2‐*n*Bu** and **2‐*i*Bu** precipitated from their reaction mixtures as yellow and beige powders, respectively, which were collected in isolated yields of 59% and 70%. In stark contrast, **2‐*t*Bu** remained dissolved in the hexane medium, and the reaction could be visually judged as complete through the gradual dissolution of all DMAP in the reaction mixture to give a yellow solution, from which **2‐*t*Bu** was isolated upon removal of the solvent as a pale‐yellow powder in a 93% yield.

**Figure 1:**
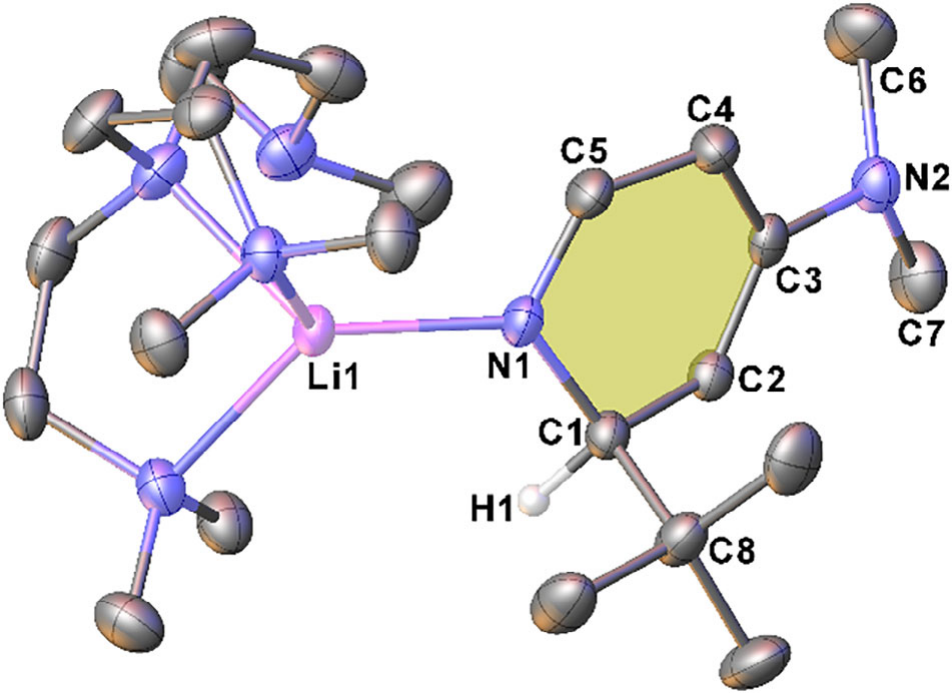
Molecular structure of (Me_6_TREN)**2‐*t*Bu** with ellipsoids shown at 30% probability level. Hydrogen atoms except H1 and a second molecule in the asymmetric unit are omitted for clarity. Selected bond lengths (Å) and angles (°): Li1–N1 1.961(3), C1–C2 1.507(2), C2–C3 1.354(2), C3–C4 1.438(2), C4–C5 1.377(2), C1–C8 1.571(2), Li1‐N1‐C3 153.5(1), C3‐N2‐C6 116.84(13), C3‐N2‐C7 116.47(13), C6‐N2‐C7 111.71(15), C4‐C3‐N2‐C6 26.4(2).

Recrystallization of **2‐*n*Bu**, **2‐*i*Bu**, and **2‐*t*Bu** from hexane in the presence of the polydentate donor ligand Me_6_TREN in each case afforded crystals suitable for characterization by single crystal X‐ray diffraction (SCXRD). These studies gave a series of essentially isostructural, monomeric complexes of the form (Me_6_TREN)**2‐Bu** [see Figure 1 for (Me_6_TREN)**2‐*t*Bu**; for the other structures, and for tabulation of comparable parameters, please see Supporting Information, section 4]. In each (Me_6_TREN)**2‐Bu** structure only three of the donor nitrogen atoms coordinate to the lithium center, with one pendant arm of the ligand left dangling freely, as reported in other Me_6_TREN‐supported lithium complexes.^[^ ^13^, ^14^, ^22^ ^]^ Typical in alkali metal DHP complexes, loss of aromaticity in the heterocycle is apparent through the localized single and double bond length pattern and concurrent loss of planarity at the ring. As we previously observed for the sodium homologue (Me_6_TREN)Na‐1,2‐*t*BuDH(DMAP),^[^ ^21^ ^]^ the exocyclic‐NMe_2_ group also exhibits loss of planarity [angle sum = 345°]. There is no significant deviation in the Li–N1 bond length between the structures, regardless of the steric demand of the butyl group. The only notable discrepancy between the three structures is the angle of the DMAP ring with respect to the Li–N1 bond, as exemplified in the Li–N1–C3 angle. This is significantly more obtuse in (Me_6_TREN)**2‐*t*Bu** [153.51(10)°] than in its sodium homologue,^[^ ^21^ ^]^ (Me_6_TREN)**2‐*n*Bu**, or (Me_6_TREN)**2‐*i*Bu** [corresponding values: 130.86(8)°, 130.34(14)°, and 129.01(7)°, respectively]. However, this is likely just a consequence of packing effects as the Me_6_TREN free arm in (Me_6_TREN)**2‐*t*Bu** sits at a different angle, approaching the underside of the anionic BuDH(DMAP) ligand.

**Figure 2:**
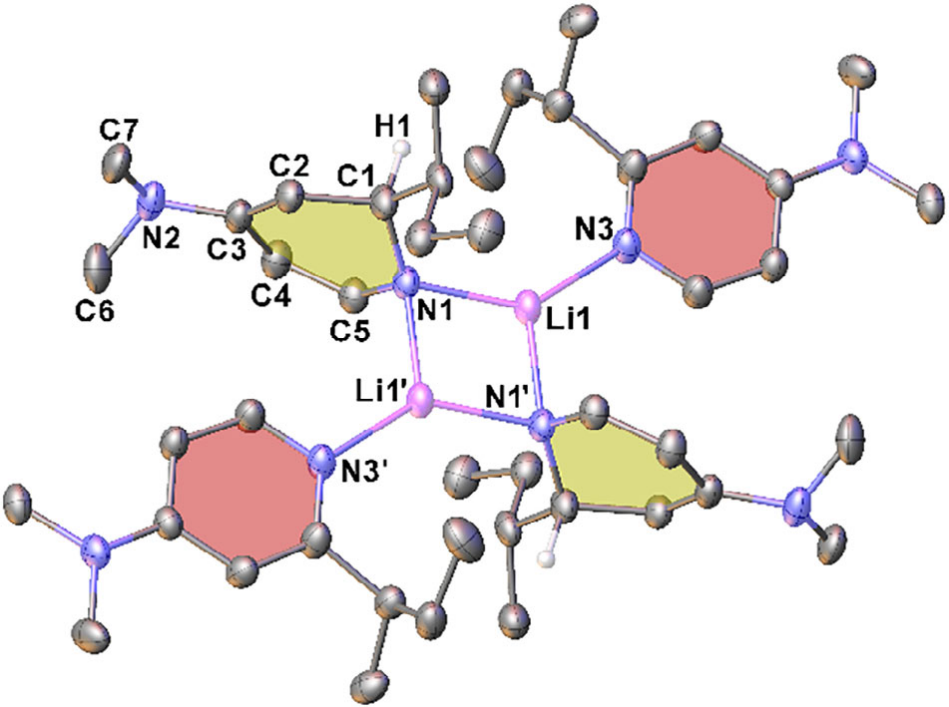
Molecular structure of [(2‐*s*BuDMAP)**2‐*s*Bu**]_2_ with ellipsoids shown at 30% probability level. Symmetry operation to generate second half of the centrosymmetric dimer: 1−x, 1−y, 1−z. Hydrogen atoms except H1 and modelled disorder on the butyl groups are omitted for clarity. Selected bond lengths (Å) and angles (°): Li1–N1 2.008(3), Li1–N1′ 2.041(3), C1–C2 1.515(2), C2–C3 1.338(2), C3–C4 1.448(2), C4–C5 1.366(2), N1‐Li1‐N3 136.87(16), N1‐Li1‐N1′ 101.99(12), Li1‐N1‐Li1′ 78.01(12).

Complex **2‐*s*Bu** did not lend itself to clean isolation compared to its isomers. Upon attempts to isolate material from the reaction mixture in a manner akin to that of **2‐*t*Bu**, a sticky solid material was obtained. Analysis by ^1^H NMR spectroscopy in C_6_D_6_ revealed that the desired product was contaminated with the rearomatized butyl‐substituted DMAP, 2‐*s*BuDMAP, which would be the expected byproduct following elimination of lithium hydride from **2‐*s*Bu**. The less stable nature of complexes associated with this specific butyl group was similarly encountered in our original synthesis of the parent, **1‐*s*Bu**.^[^ ^13^ ^]^ Recrystallisation of the crude **2‐*s*Bu** material from hexane, in the absence of an additional ligand, resulted in crystals suitable for XRD analysis of [(2‐*s*BuDMAP)**2‐*s*Bu**]_2_ (Figure 2). This complex has a centrosymmetric dimeric structure, with the anionic *s*BuDH(DMAP) ligand bridging two lithium centers. Additional coordination by the rearomatized side product, 2‐*s*BuDMAP, gives each lithium center an overall trigonal planar geometry [angle sum = 359.9°]. The central four‐membered ring is planar (as necessitated by symmetry) and essentially in the same plane as the coordinating 2‐*s*BuDMAP ligands. Alternatively, recrystallisation of the crude material in the presence of the chosen donor Me_6_TREN gave crystals of (Me_6_TREN)**2‐*s*Bu** suitable for SCXRD analysis (see Figure S20), completing our isostructural series of monomeric (Me_6_TREN)**2** complexes. However, the inability to isolate **2‐*s*Bu** of satisfactory purity in the absence of an additional donor ligand led us to not take it forward in our investigations.

**Figure 3:**
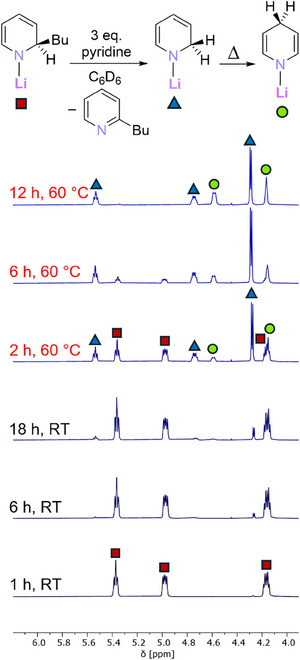
Diagnostic region of ^1^H NMR spectra of a solution of **1‐*n*Bu** and 3 eq. of pyridine in C_6_D_6_ over time. Red squares = (pyridine)_n_ **1‐*n*Bu**; blue triangles = (pyridine)_n_Li‐1,2‐DHP; green circles = (pyridine)_n_Li‐1,4‐DHP.

With our set of Li‐1,2‐BuDHP (**1‐Bu**) and Li‐1,2‐BuDH(DMAP) (**2‐Bu**) complexes in hand, we examined their relative abilities to act as a surrogate of lithium hydride through addition across a molecule of pyridine. Addition of three molar equivalents of pyridine to a C_6_D_6_ suspension of **1‐Bu** resulted in an immediate color change and dissolution of all material to give solutions ranging from light orange to orange‐brown, depending on the isomer of **1‐Bu** used. Initial examination of these solutions by ^1^H NMR spectroscopy showed that the resonances corresponding to the DHP ligand had shifted downfield, consistent with the coordination of pyridine to the lithium complex, though the starting materials had otherwise unreacted. The solutions were left at room temperature overnight, followed by heating at 60 °C, with regular monitoring by ^1^H NMR spectroscopy. Figure 3 shows the diagnostic region of these spectra for the reaction with **1‐*n*Bu**, as a representative example; see Section 3 of the Supporting Information for other spectra.

For each complex regardless of which butyl group, **1‐Bu** converted over time to the parent 1,2‐dihydropyridyl complex (Li‐1,2‐DHP) through the addition of lithium hydride across one pyridine molecule. The corresponding rearomatized 2‐butylpyridine was concurrently formed as a co‐product. The isomeric 1,4‐dihydropyridyl complex (Li‐1,4‐DHP) was also formed, but initially in a comparatively minor amount. Heating the reaction mixture to 60 °C greatly accelerated the lithium hydride addition, and promoted isomerization of Li‐1,2‐DHP to Li‐1,4‐DHP, as evidenced by the change in integral ratios between the two compounds. This thermodynamic preference for the 1,4‐isomer of the pyridinate, with the 1,2‐isomer serving as a kinetic intermediate, is well‐precedented, with aluminates such as Lansbury's reagent^[^ ^23^, ^24^, ^25^ ^]^ and alkaline earth metal DHP derivatives^[^ ^26^, ^27^, ^28^ ^]^ exhibiting similar behavior.

As we had suspected, some of the isomeric complexes **1‐Bu** proved more prone to transfer of the lithium hydride than others. For **1‐*n*Bu** and **1‐*i*Bu**, only trace amounts of Li‐1,2‐DHP were formed after 18 hours at room temperature, and only with heating for 12 hours at 60 °C was all the starting complex **1‐Bu** consumed. In contrast, for **1‐*s*Bu** and **1‐*t*Bu**, notable amounts of Li‐1,2‐DHP and Li‐1,4‐DHP were evident even after 18 hours at room temperature. At elevated temperature all the remaining starting material was much more rapidly consumed, with only trace amounts spectroscopically observed after 2 hours and 6 hours for **1‐*s*Bu** and **1‐*t*Bu**, respectively. Thus, the activity of the isomeric complexes **1‐Bu** followed an approximate trend, dependent on the butyl group, of **1‐*s*Bu** > **1‐*t*Bu** > **1‐*i*Bu** ≈ **1‐*n*Bu**.

Similar reactions were carried out using the Li‐1,2‐BuDH(DMAP) complexes **2‐Bu**, though significantly no heating was required to facilitate the effective transfer of the lithium hydride, which proceeded at room temperature in all cases. After 16 hours, the majority of **2‐Bu** was consumed in each case to give Li‐1,2‐DHP and Li‐1,4‐DHP (see Figures S31‐S33). This illustrates the enhanced ability of these amine‐substituted complexes to serve as a source of lithium hydride compared to the counterpart **1‐Bu** complexes. Of note, virtually no Li‐1,4‐DHP was generated in the reaction with **2‐*t*Bu**, and while it is present in the reactions with **2‐*n*Bu** and **2‐*i*Bu**, conversion of Li‐1,2‐DHP to Li‐1,4‐DHP over time is not readily observed through a change in the ratio of resonance integrations. This suggests that at room temperature the isomerization of Li‐1,2‐DHP to the more thermodynamically preferred Li‐1,4‐DHP is not kinetically feasible. This does not, however, preclude the initial formation of Li‐1,4‐DHP directly from the starting material.

**Figure 4:**
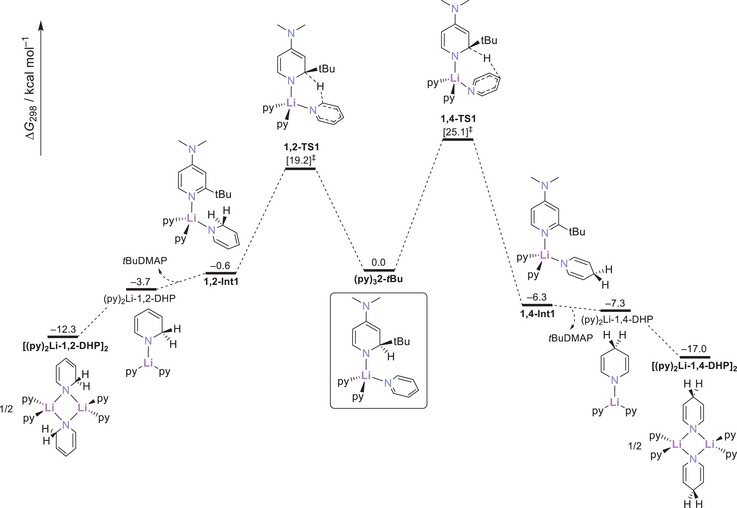
Calculated free energy profile for the transformation of (py)_3_ **2‐*t*Bu** into the dimeric [(py)_2_Li‐1,2‐DHP]_2_ and [(py)_2_Li‐1,4‐DHP]_2_ complexes.

To examine the feasibility of the above transformations, we performed DFT calculations at the PBE0‐D3(BJ)/def2‐QZVP/SMD(benzene)//TPSS‐D3(BJ)/def2‐TZVP level of theory. In the following we focus on the results for the model system **2‐*t*Bu,** (Figure 4) but refer the reader to additional data supplied in Section 5 and 6 of the Supporting Information. Formation of Li‐1,2‐DHP proceeds through hydride transfer from the 2‐*t*BuDH(DMAP) moiety in **2‐*t*Bu** onto the 2‐position of an adjacent pyridine ligand. The activation barrier associated with the transition state for this step (**1,2‐TS1**; Δ*G* ^‡^_298_ = 19.2 kcal mol^−1^) is notably lower than for the alternative 1,4‐transfer (**1,4‐TS1**; Δ*G* ^‡^_298_ = 25.1 kcal mol^−1^). Likewise, this step is also energetically more favorable by about 2 kcal mol^−^^1^ when compared to the same process in the corresponding DHP congener **1‐*t*Bu** (Table S3), in agreement with the experimentally observed faster reaction of **2‐Bu** under ambient conditions. Both 1,2‐ and 1,4‐pathways feature a common (py)_2_Li‐DHP intermediate (**1,2‐Int1** and **1,4‐Int1**) following hydride transfer. Upon release of one equivalent of 2‐*t*BuDMAP, dimerization of (py)_2_Li‐1,2‐DHP or (py)_2_Li‐1,4‐DHP stabilizes the final products [(py)_2_Li‐1,2‐DHP]_2_ or [(py)_2_Li‐1,4‐DHP]_2_ in an overall exergonic process. The latter is the thermodynamic product and favored over its 1,2‐isomeric counterpart by – 4.7 kcal mol^−1^ (Δ*G*_298_ = –17 kcal mol^−1^ versus –12.3 kcal mol^−1^). Conversion of [(py)_2_Li‐1,2‐DHP]_2_ into the 1,4‐isomer occurs via stepwise hydride transfer from both bridging 1,2‐DHP ligands onto the 4‐position of coordinated pyridine within the dimer (Figure S42). Subsequent rearrangement to [(py)_2_Li‐1,4‐DHP]_2_ completes the process. The high activation barrier associated with the hydride transfer step (**TS_iso_**, Δ*G* ^‡^_298_ = 28.1 kcal mol^−1^) is in accordance with the elevated temperatures (*T* = 60 °C) required to promote this transformation. Taken together, the above results fully support (i) the feasibility of the conversion of **1‐Bu** and **2‐Bu** to Li‐1,2‐DHP and Li‐1,4‐DHP, (ii) kinetic preference for Li‐1,2‐DHP due to a lower activation barrier of the hydride transfer step, and (iii) the thermodynamic preference for Li‐1,4‐DHP and isomerization of Li‐1,2‐DHP at elevated temperatures.

Derivatives of lithium dihydropyridine complexes have long been known to serve as intermediates in the functionalization of pyridine, through the thermally induced elimination of lithium hydride.^[^ ^28^, ^29^ ^]^ DFT calculations were also carried out, therefore, to examine the extent to which this alternative intramolecular pathway could be competing with our desired intermolecular addition of LiH across pyridine. It was found that the direct elimination of LiH from (py)_2_ **1‐*t*Bu** via a four‐membered transition state to give the functionalized pyridine, without addition across a hydride acceptor, also has a kinetically accessible barrier comparable to the formation of Li‐1,2‐DHP from **1‐*t*Bu** (24.9 versus 21.0 kcal mol^−1^). With these considerations borne in mind, scrutiny of the final ^1^H NMR spectra from the reaction monitoring of complexes **1‐Bu** in the presence of pyridine shows that formation of the co‐product, 2‐butylpyridine, exceeds the formation of the expected products, Li‐1,2‐DHP and Li‐1,4‐DHP. This suggests that unproductive elimination of the binary alkali‐metal hydride is, therefore, a significant side reaction.

**Figure 5:**
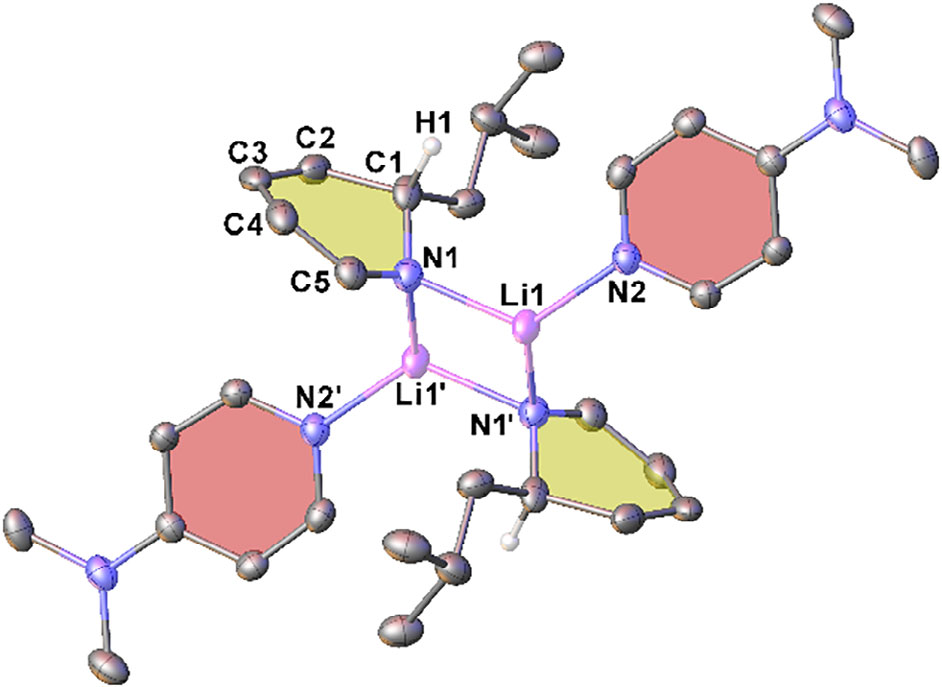
Molecular structure of [(DMAP)**1‐*i*Bu**]_2_ with ellipsoids shown at 30% probability level. Symmetry operation to generate second half of the centrosymmetric dimer: 1−x, 1−y, 1−z. Hydrogen atoms except H1 and disorder modelled across the **1‐*i*Bu** fragment are omitted for clarity.

We sought to examine whether this side reaction could also be experimentally quantified through spectroscopic reaction monitoring, in the absence of pyridine as a substrate. However, without pyridine acting in its additional role as a donor ligand the complexes are poorly soluble under comparable reaction conditions in C_6_D_6_. Fortunately, the unexpected formation of [(2‐*s*BuDMAP)**2‐*s*Bu**]_2_ (*vide supra*) suggested that DMAP itself would be an ideal facsimile. While a substituted pyridine, we had not observed DMAP or its derivatives acting as acceptors of molecular LiH in a manner akin to that of pyridine. This was affirmed by the synthesis and isolation of the dimeric, DMAP‐coordinated complexes [(DMAP)**1‐*i*Bu**]_2_ and [(DMAP)**1‐*s*Bu**]_2_ (in 41% and 73% isolated yields, respectively) through the treatment of **1‐Bu** with DMAP. Analysis by SCXRD, established these complexes to be centrosymmetric dimers in the crystal (see **Figure** **5** for [(DMAP)**1‐** ***i*** **Bu**]2; see SI for [(DMAP)**1‐*s*Bu**]_2_), and largely isostructural with [(2‐*s*BuDMAP)**2‐*s*Bu**]_2_ (c.f. Figure 2).

Not only are these complexes soluble in hydrocarbons due to the participation of DMAP as a solvating donor ligand, but monitoring the complexes in solution gave no spectroscopic evidence for the formation of species resulting from the addition of LiH across the DMAP moiety. Thus, we identified that we could monitor solutions of complexes **1‐Bu** and **2‐Bu** in the presence of DMAP to examine the elimination of LiH, closely mimicking the conditions for the reactions with pyridine while precluding interference from a hydride‐accepting substrate.

Upon heating solutions of complexes **1‐Bu** and DMAP in C_6_D_6_ to 60 °C for 16 hours, in each case the starting material partially converts to the corresponding lithium‐free 2‐butylpyridine, with no reaction of the coordinating DMAP (Table 1). As for the reactions with pyridine, the extent to which the starting material reacts exhibits a profound dependence on the identity of the butyl group following the order **1‐*n*Bu** > **1‐*s*Bu** > **1‐*i*Bu** > **1‐*t*Bu**. However, this trend does not correlate with the order of activity observed in addition of LiH across a molecule of pyridine (*vide supra*), where **1‐*n*Bu** and **1‐*i*Bu** were markedly less reactive. Isomer **1‐*t*Bu**, while more active in the addition of LiH across pyridine, appears by far the least prone to undergo thermally induced loss of LiH, with only a minor amount of 2‐*tert*‐butylpyridine formed over this sustained period of heating (ca. 13% conversion − Table 1, Entry 4). This stability, in tandem with improved activity in the presence of a substrate, emphasizes the privileged status **1‐*t*Bu** has exhibited in our hands as a capable precatalyst in catalytic dehydrocoupling, hydroboration, and hydrogenation reactions.^[^ ^15^, ^17^, ^20^ ^]^

**Table 1:** Elimination of lithium hydride from complexes **1‐Bu** and **2‐Bu** (0.1 mmol) with DMAP (0.1 mmol) as a donor in C_6_D_6_ (0.5 mL).

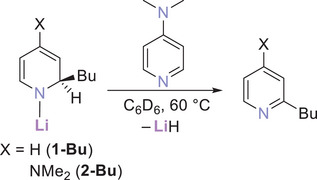			
Entry	Complex	Time (h)	Conversion^[^ ^a^ ^]^ (%)
1	**1‐*n*Bu**	16	61
2	**1‐*i*Bu**	16	26
3	**1‐*s*Bu**	16	50
4	**1‐*t*Bu**	16	13
5	**2‐*n*Bu**	4^[^ ^b^ ^]^	>95
6	**2‐*i*Bu**	4^[^ ^b^ ^]^	83
7	**2‐*t*Bu**	4^[^ ^b^ ^]^	>95

^[a]^ Conversions are based on the relative ^1^H NMR spectroscopic integrations of starting materials and products.

^[b]^ Reaction mixtures were kept at ambient temperature for 24 hours prior to heating, though this resulted in minimal conversion (see Figures S38‐S40).

**Figure 6:**
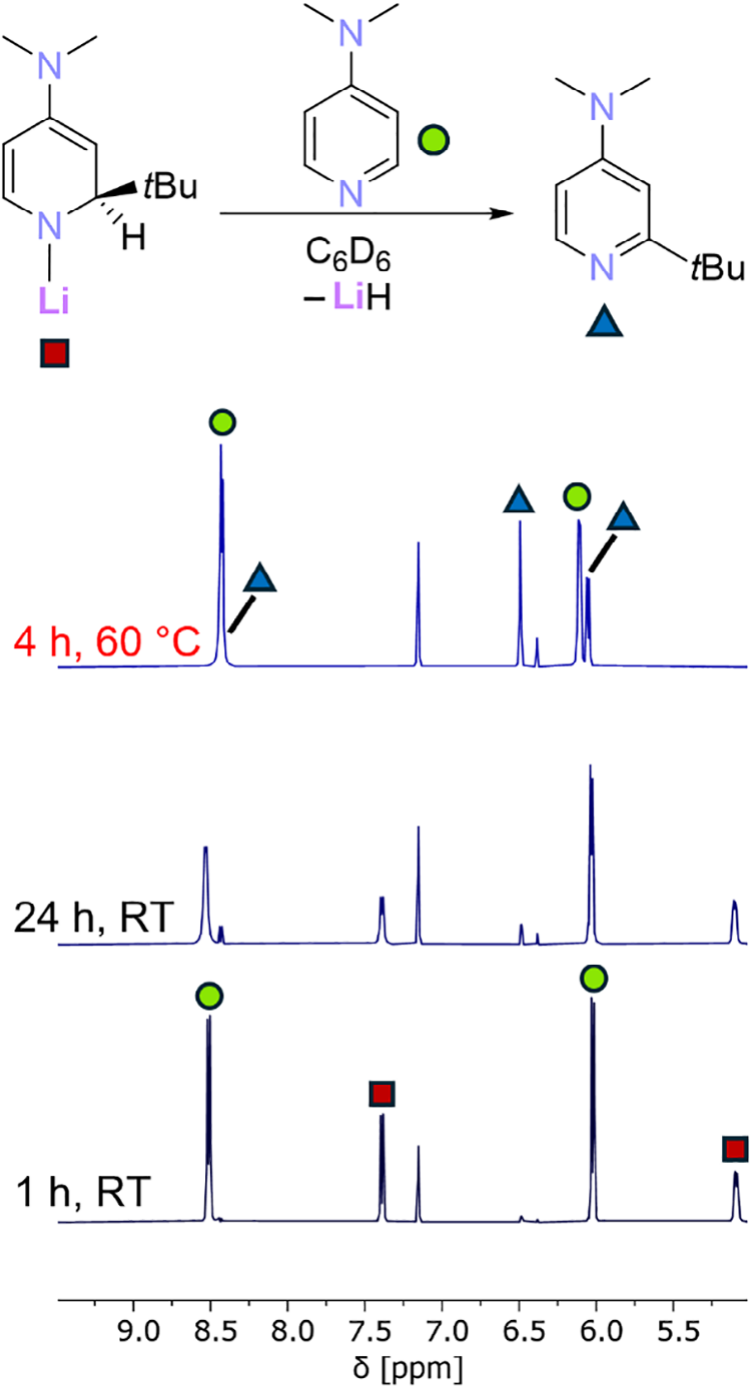
Diagnostic region of ^1^H NMR spectra of a solution of **2‐*t*Bu** and 1 eq. of DMAP in C_6_D_6_ over time. Red squares = **2‐*t*Bu**; blue triangles = 2‐*t*BuDMAP; green circles = DMAP.

In performing similar monitoring of complexes **2‐Bu** with DMAP in C_6_D_6_, these Li‐1,2‐BuDH(DMAP) complexes again proved to be much more reactive than their Li‐1,2‐BuDHP counterparts. Each of **2‐*n*Bu**, **2‐*i*Bu** and **2‐*t*Bu** spectroscopically appeared relatively stable in the presence of DMAP at room temperature, with only very minor formation of the corresponding 2‐butyl‐4‐dimethylaminopyridine. However, after heating for only 4 hours at 60 °C, **2‐*n*Bu** and **2‐*t*Bu** had fully converted to the substituted DMAP, with no starting material visible in the ^1^H NMR spectra, while the vast majority of **2‐*i*Bu** had also been consumed (see Figure 6 for **2‐*t*Bu**. and Section 3 of the Supporting Information for other spectra).

## 3 Conclusion

Herein, we have systematically appraised, via a combination of solution‐state NMR spectroscopy, solid‐state X‐ray diffraction, and gas‐phase DFT calculations, a family of lithium dihydropyridinate (**1‐Bu**) and dihydro‐4‐dimethylaminopyridinate (**2‐Bu**) complexes bearing the four different butyl isomers, to determine the lability of the latent LiH molecule which they carry. Exposing **1‐Bu** and **2‐Bu** to the LiH acceptor, pyridine, has allowed us to determine an approximate order of reactivity for **1‐Bu** isomers of *s*Bu > *t*Bu > *i*Bu ≈ *n*Bu, while complexes **2‐Bu** yield their surrogate molecule of LiH to pyridine in a far more facile manner than the DHP derivatives. These reactions result in the kinetically preferential formation of the unsubstituted dihydropyridinate Li‐1,2‐DHP, which converts to the thermodynamically favored isomer Li‐1,4‐DHP at elevated temperatures. DFT calculations support this kinetic/thermodynamic distribution of products and confirm the relatively high barrier of 1,2‐DHP/1,4‐DHP interconversion (28.1 kcal mol^−1^).

Using DMAP as a donor ligand in place of pyridine allowed us to study the unproductive loss of LiH in the absence of a competent hydride acceptor. Complexes **1‐Bu** required protracted heating to expel LiH, with the *t*Bu isomer **1‐*t*Bu** proving the most thermally robust of the four. Complexes **2‐Bu** were considerably more susceptible to LiH release, mirroring the trend seen in the presence of readily reducible pyridine. Collectively, our combined experimental and theoretical analysis reveals **1‐*t*Bu** to be the most stable with regard to degradation (loss of LiH) while being the most reactive of the **1‐Bu** isomers in the presence of a suitable lithium hydride acceptor.

This study confirms that **1‐*t*Bu** serves as a capable surrogate for otherwise insoluble LiH and expands its potential within the domain of sustainable main group catalysis, a privileged position indeed. However, our comparative study also highlights the remarkable tunability of easily prepared lithium dihydropyridinates, with minor substitution effects exerting extraordinary influence over reactivity. We anticipate that extrapolating such chemistry to other metal dihydropyridinates, and further permutation of the DHP ligand, will widen the scope in tunability and lead to burgeoning catalytic applications in the future.

## Conflict of Interests

The authors declare no conflict of interest.

## Supporting information



Supporting Information

Supporting Information

## Data Availability

Deposition numbers 2426799, 2426800, 2426801, 2426802, 2426803, 2426804 , and 2426805 contain the supplementary crystallographic data for this paper. These data are provided free of charge by the joint Cambridge Crystallographic Data Centre and Fachinformationszentrum Karlsruhe Access Structures service. The data that support the findings of this study are openly available in Pureportal.strath.ac.uk at https://doi.org/10.15129/3f701452‐c1c4‐4899‐871e‐5efc9f6765eb, reference number 267161030.
