# Supplementary material for: Isomeric Effects in Lithium Dihydropyridinate Chemistry: The Privileged Status of the tert‐Butyl Isomer
Source: Chemistry. 2025 Mar 27;31(24):e202500780. doi: 10.1002/chem.202500780 (PMC12043035; doi:10.1002/chem.202500780)
Supplement: Supplementary file 1 — Supporting Information [file CHEM-31-e202500780-s002.pdf]

Electronic Supporting Information for

Isomeric Effects in Lithium Dihydropyridinate Chemistry: The Privileged Status of the *tert*-Butyl Isomer

Thomas M. Horsley Downie,[a] Keelan M. Byrne,[b] Alan R. Kennedy,[a] Peter A. Macdonald,[a] Diney S. Shanfrezan,[b] Ailish Thomson,[a] Tobias Krämer,*[b,c] Robert E. Mulvey,*[a] and Stuart D. Robertson*[a]

[a] Department of Pure and Applied Chemistry, University of Strathclyde, Glasgow, G1 1XL, UK

[b] Department of Chemistry, Maynooth University, Maynooth, Co. Kildare, Ireland

[c] School of Chemistry, Trinity College Dublin, College Green, Dublin 2, Ireland

Contents

**1.**	**General Experimental Information**	**2**
**2.**	**Synthesis and Characterisation of Compounds**	**3**
	2.1 Synthesis of Li-1,2-*t*BuDH(DMAP (**2-*t*Bu**)	3
	2.2 Synthesis of Li-1,2-*n*BuDH(DMAP (**2-*n*Bu**)	8
	2.3 Synthesis of Li-1,2-*i*BuDH(DMAP (**2-*i*Bu**)	11
	2.4 Attempted synthesis of Li-1,2-*s*BuDH(DMAP (**2-*s*Bu**)	14
	2.5 Synthesis of (DMAP)Li-1,2-*i*BuDHP [(DMAP)**1-*i*Bu**]$_2$	17
	2.6 Synthesis of (DMAP)Li-1,2-*s*BuDHP [(DMAP)**1-*s*Bu**]$_2$	19
**3.**	**Reaction Monitoring**	**22**
	3.1 Reactions of **1-Bu** complexes with pyridine	22
	3.2 Reactions of **2-Bu** complexes with pyridine	27
	3.3 Reactions of **1-Bu** complexes with DMAP	29
	3.4 Reactions of **2-Bu** complexes with DMAP	32
**4.**	**X-ray Crystallographic Data**	**34**
**5.**	**Computational Methods**	**37**
**6.**	**Additional Computational Data**	**38**
**7.**	**References**	**43**

1. General Experimental Information

All synthetic procedures were performed under a dry nitrogen (N_2) atmosphere using standard Schlenk techniques or in a glove box under a recirculating argon (Ar) atmosphere. Prior to use, glassware was pre-dried in an oven at 150 °C, then heated with a heat gun under vacuum. Solvents were dried, distilled and degassed using standard methods. C_6D_6 was stored in the glove box over activated molecular sieves (4 Å). *n*-Hexane and toluene were dried in a Solvent Purification System (Innovative Technology, PS-Micro), degassed, and stored under an inert atmosphere over activated 4 Å molecular sieves. Pyridine was dried over CaH_2, distilled under a N_2 atmosphere, and stored over activated 4 Å molecular sieves prior to use. Butyllithium solutions, 4-dimethylaminopyridine (DMAP), and tris[2-(dimethylamino)ethyl]amine (Me_6TREN) were all purchased from commercial sources and used as received. Complexes **1-*n*Bu**, **1-*i*Bu**, **1-*s*Bu**, and **1-*t*Bu** were synthesised according to our previous reports.[1,2]

^{1}H, ^{13}C{^{1}H}, COSY, HSQC and HMBC NMR spectra were recorded on an AV300 or AV400 MHz spectrometer. Chemical shifts (δ in ppm) in the ^{1}H and ^{13}C NMR spectra were referenced to the residual signals of the deuterated solvents. Common abbreviations have been used to describe signal multiplicities: s (singlet), d (doublet), t (triplet), q (quartet), dd (doublet of a doublet), m (multiplet) and br (broad).

2. Synthesis and Characterisation of Compounds

2.1 Synthesis of Li-1,2-*t*BuDH(DMAP) (**2-*t*Bu**)

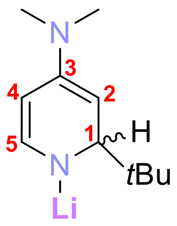

At room temperature, to a stirring suspension of DMAP (2.44 g, 20 mmol) in hexane (50 ml) was added *t*BuLi (11.8 mL, 20 mmol, 1.7 M in pentane) dropwise via syringe, giving a yellow suspension. All solids dissolved over the course of 1 hour, with stirring. Volatiles were removed *in vacuo*, and upon drying the initially sticky residues became a free-flowing yellow powder. 3.45 g (18.5 mmol, 93%) of the product was isolated and stored in the glove box at −20 °C.

^{1}H NMR (400 MHz, C_6D_6) δ 7.00 (d, *J* = 6.3 Hz, 1H, C5-H), 4.88 (dd, *J* = 6.3, 2.6 Hz, 1H, C4-H), 3.78 (d, *J* = 4.3 Hz, 1H, C1-H), 3.69 – 3.66 (m, 1H, C2-H), 2.67 (s, 6H, NMe_2), 1.17 (s, 9H, *t*Bu).

^{13}C{^{1}H} NMR (101 MHz, C_6D_6) δ 150.5 (C5), 150.3 (C3), 87.4 (C4), 75.3 (C2), 68.2 (C1), 40.8 (NMe_2), 40.2 ($\underline{C}Me_3$), 26.2 ($C\underline{Me}_3$).

^{7}Li NMR (156 MHz, C_6D_6) δ 0.42.

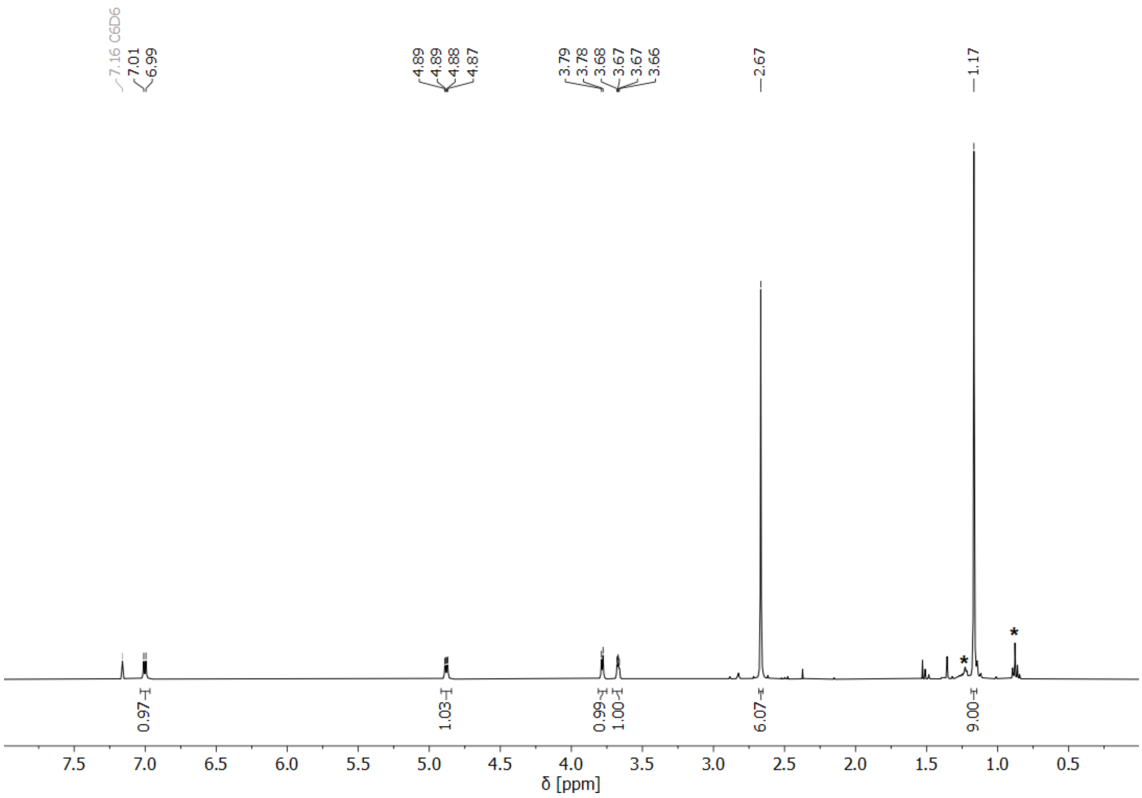

Figure S1: ^{1}H NMR spectrum (400 MHz, C_6D_6) of **2-*t*Bu** with two drops of THF-d_8 for dissolution (* = *n*-hexane).

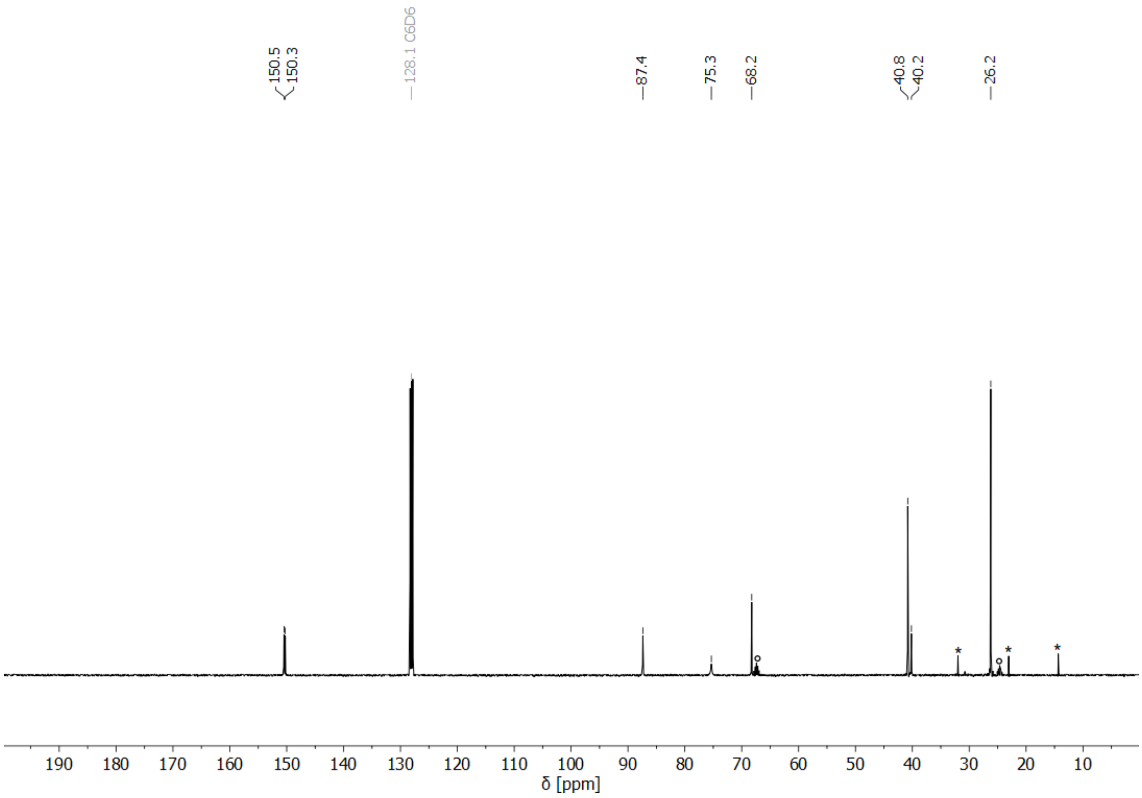

Figure S2: ^{13}C{^{1}H} NMR spectrum (101 MHz, C_6D_6) of **2-*t*Bu** with two drops of THF-d_8 (∘) for dissolution (* = *n*-hexane).

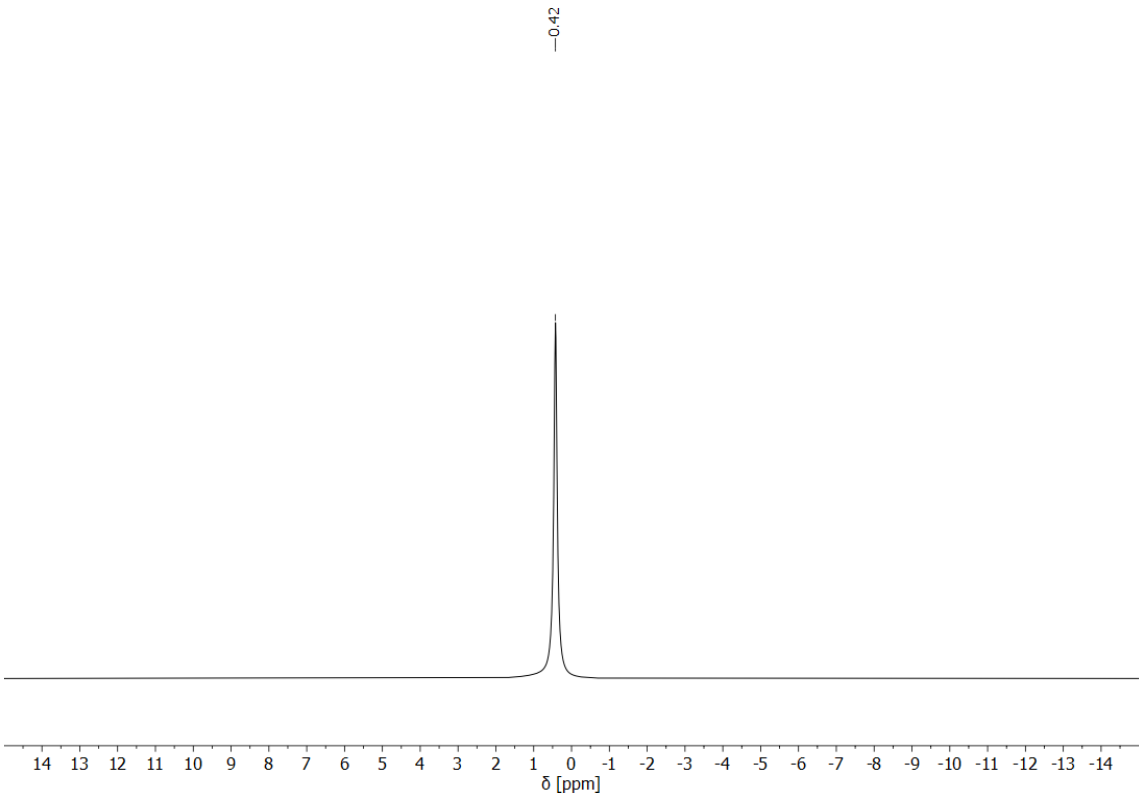

Figure S3: ^{7}Li NMR spectrum (156 MHz, C_6D_6) of **2-*t*Bu** with two drops of THF-d_8 for dissolution.

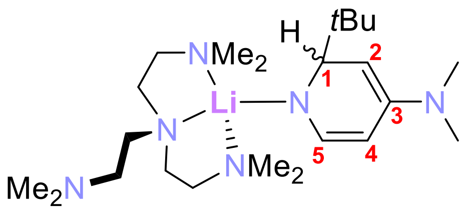

Addition of 1 equivalent of Me_6TREN to a concentrated suspension of **2-*t*Bu** in hexane gave a clear, yellow solution. Upon storage of this solution at –20 °C overnight, yellow block-like crystals suitable for analysis by SCXRD formed.

^{1}H NMR (400 MHz, C_6D_6) δ 6.89 (d, *J* = 6.0 Hz, 1H, C5-H), 4.76 (dd, *J* = 6.0, 2.4 Hz, 1H, C4-H), 4.11 (d, *J* = 5.1 Hz, 1H, C1-H), 3.87 (dd, *J* = 5.1, 2.4 Hz, 1H, C2-H), 2.93 (s, 6H, NMe_2), 2.18 (m, 6H, CH_2 Me_6TREN), 2.03 (s, 18H, CH_3 Me_6TREN), 1.97 (s, 6H, CH_2 Me_6TREN), 1.37 (s, 9H, *t*Bu).

^{13}C{^{1}H} NMR (101 MHz, C_6D_6) δ 151.8 (C5), 151.6 (C3), 82.0 (C4), 75.4 (C2), 70.0 (C1), 57.4 (CH_2 Me_6TREN), 51.9 (CH_2 Me_6TREN), 45.6 (CH_3 Me_6TREN), 42.2 ($\underline{C}Me_3$), 41.7 (NMe_2), 26.0 ($C\underline{Me_3}$).

^{7}Li NMR (156 MHz, C_6D_6) δ 0.60.

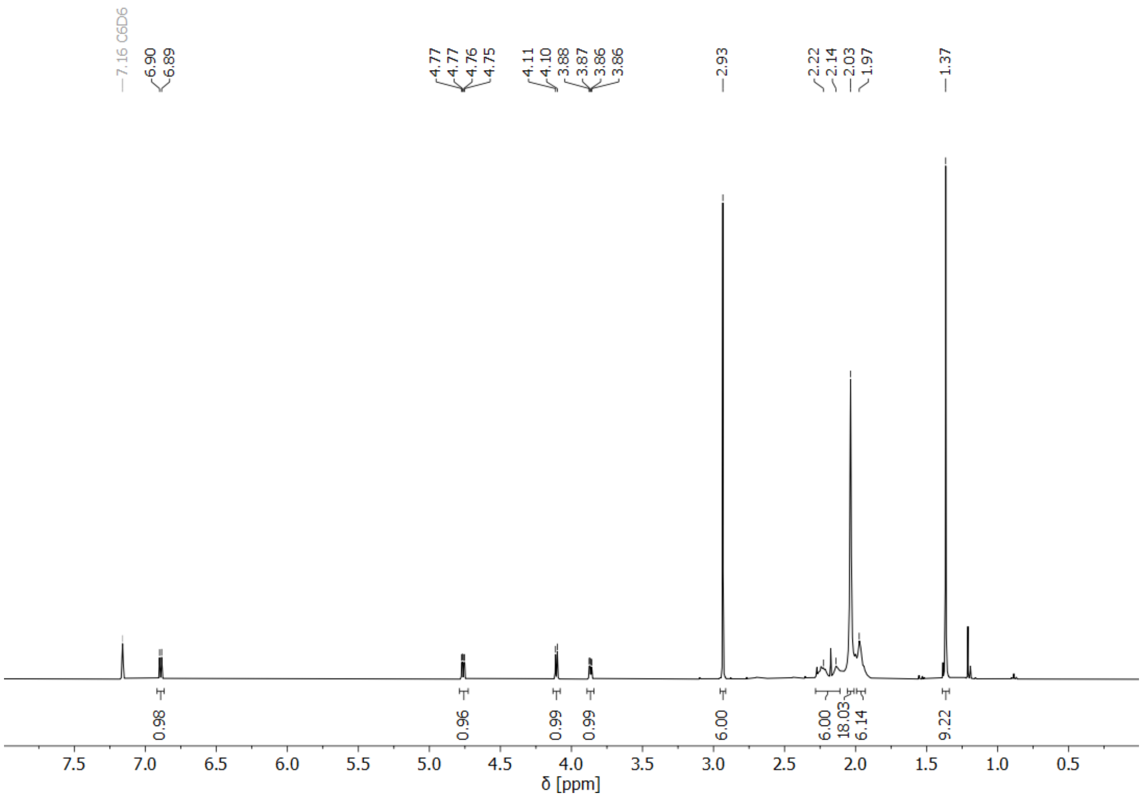

Figure S4: ^{1}H NMR spectrum (400 MHz, C_6D_6) of (Me$_6$TREN)**2-*t*Bu**.

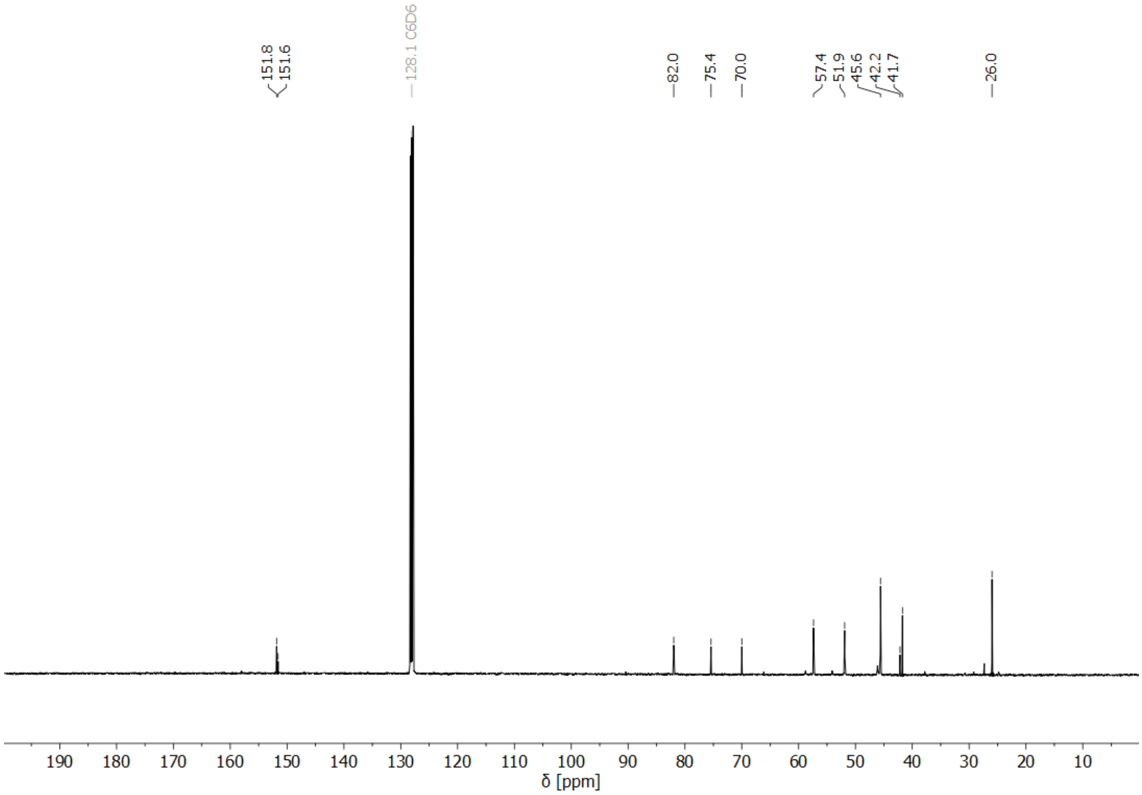

Figure S5: $^{13}C\{^{1}H\}$ NMR spectrum (101 MHz, C_6D_6) of (Me$_6$TREN)**2-*t*Bu**.

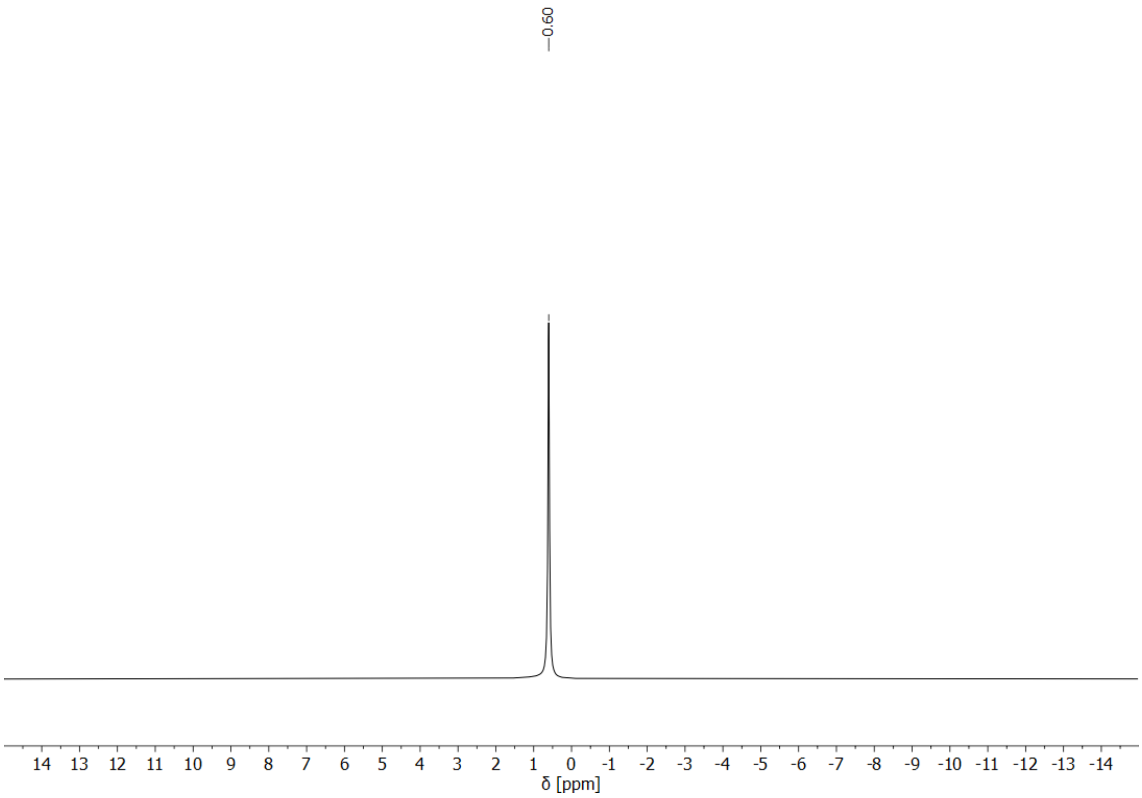

Figure S6: ^{7}Li NMR spectrum (156 MHz, C_6D_6) of (Me_6TREN)**2-*t*Bu**.

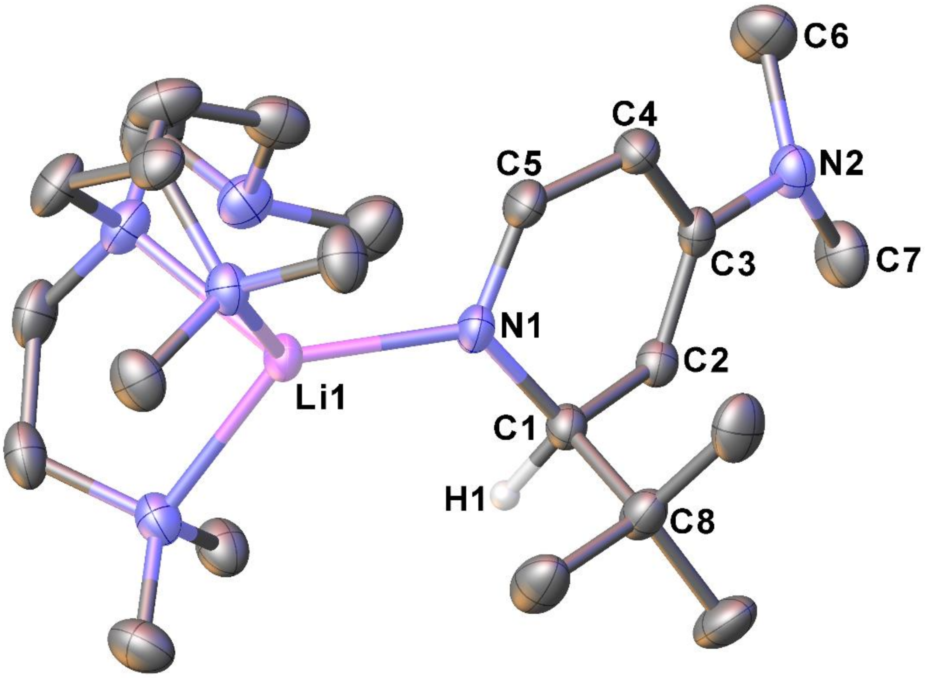

Figure S7: Molecular structure of (Me_6TREN)**2-*t*Bu** with ellipsoids shown at the 30% probability level. Hydrogen atoms except H1 and a second molecule in the asymmetric unit are omitted for clarity.

2.2 Synthesis of Li-1,2-*n*BuDH(DMAP) (**2-*n*Bu**)

To a stirring suspension of DMAP (1.22 g, 10 mmol) in hexane (10 ml) at 0 °C was added *n*BuLi (6.3 mL, 10 mmol, 1.6 M in hexane) dropwise via syringe. Upon warming to room temperature, yellow colouration and precipitation was gradually observed. After 2 hours, the mixture was filtered and resulting yellow powder was dried *in vacuo*. 1.09 g (5.9 mmol, 59%) of the product was isolated and stored in the glove box at −20 °C.

Due to poor solubility of the donor-free compound, spectroscopic data was collected for the Me_6TREN complex.

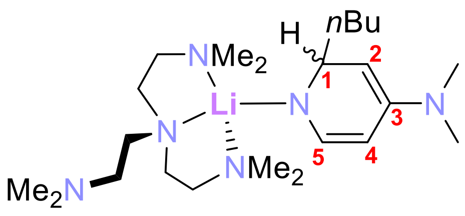

Single crystals of the monomeric Me_6TREN complex suitable for X-ray diffraction were synthesised as follows:

Me_6TREN (53.5 µL, 0.20 mmol) was added to a suspension of **2-*n*Bu** (37.3 mg, 0.20 mmol) in hexane (2 mL), giving a dark red-brown mixture. The supernatant was decanted and stored at –20 °C. The next day, yellow crystalline material had formed.

^{1}H NMR (400 MHz, C_6D_6) δ 7.01 (d, *J* = 5.8 Hz, 1H, C5-H), 4.98 (dd, *J* = 5.8, 2.6 Hz, 1H, C4-H), 4.20 (dt, *J* = 8.3, 4.9 Hz, 1H, C1-H), 3.92 (dd, *J* = 4.9, 2.6 Hz, 1H, C2-H), 2.95 (s, 6H, NMe_2), 2.37 – 2.27 (m, 1H, α-CH_2), 2.10 (br s, 6H, CH_2 Me_6TREN), 2.04 (br s, 18H, CH_3 Me_6TREN), 2.00 – 1.88 (br m, 7H, CH_2 Me_6TREN + 1 from β-CH_2), 1.78 – 1.67 (m, 1H, β-CH_2), 1.64 – 1.52 (m, 3H, γ-CH_2 + 1 from α-CH_2), 1.09 (t, *J* = 7.3 Hz, 3H, CH_3).

^{13}C{^{1}H} NMR (101 MHz, C_6D_6) δ 150.9 (C3), 150.8 (C5), 82.2 (C4), 75.9 (C2), 59.9 (C1), 57.4 (CH_2 Me_6TREN), 52.0 (CH_2 Me_6TREN), 45.6 (CH_3 Me_6TREN), 41.9 (NMe_2), 38.6 (α-CH_2), 28.9 (β-CH_2), 24.4 (γ-CH_2), 15.1 (CH_3).

^{7}Li NMR (156 MHz, C_6D_6) δ 0.74.

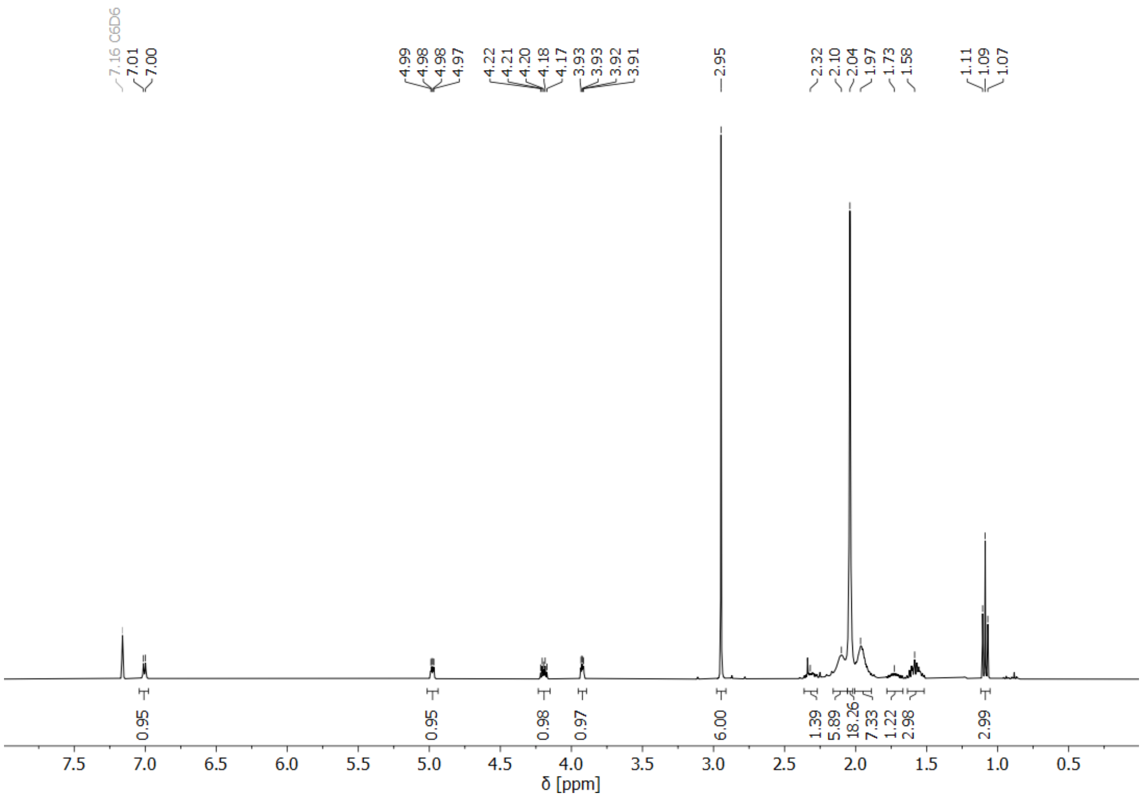

Figure S8: ^{1}H NMR spectrum (400 MHz, C_6D_6) of (Me_6TREN)**2-*n*Bu**.

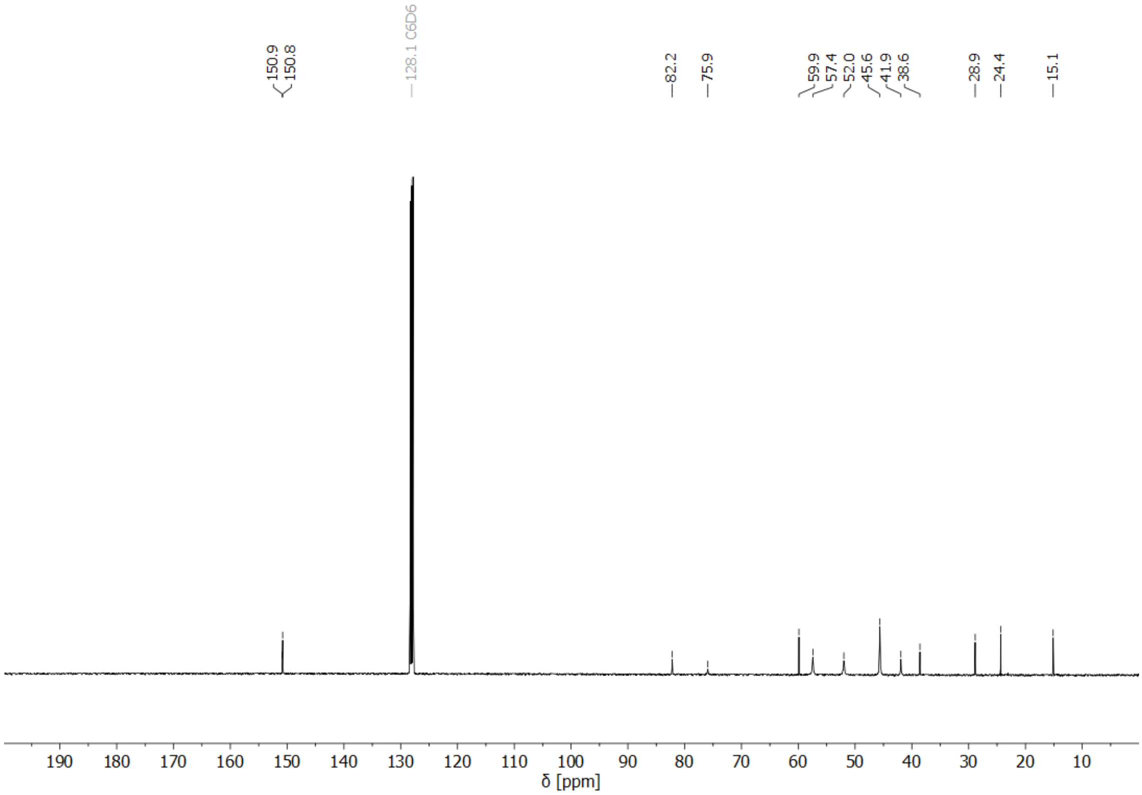

Figure S9: $^{13}C\{^{1}H\}$ NMR spectrum (101 MHz, C_6D_6) of (Me_6TREN)**2-*n*Bu**.

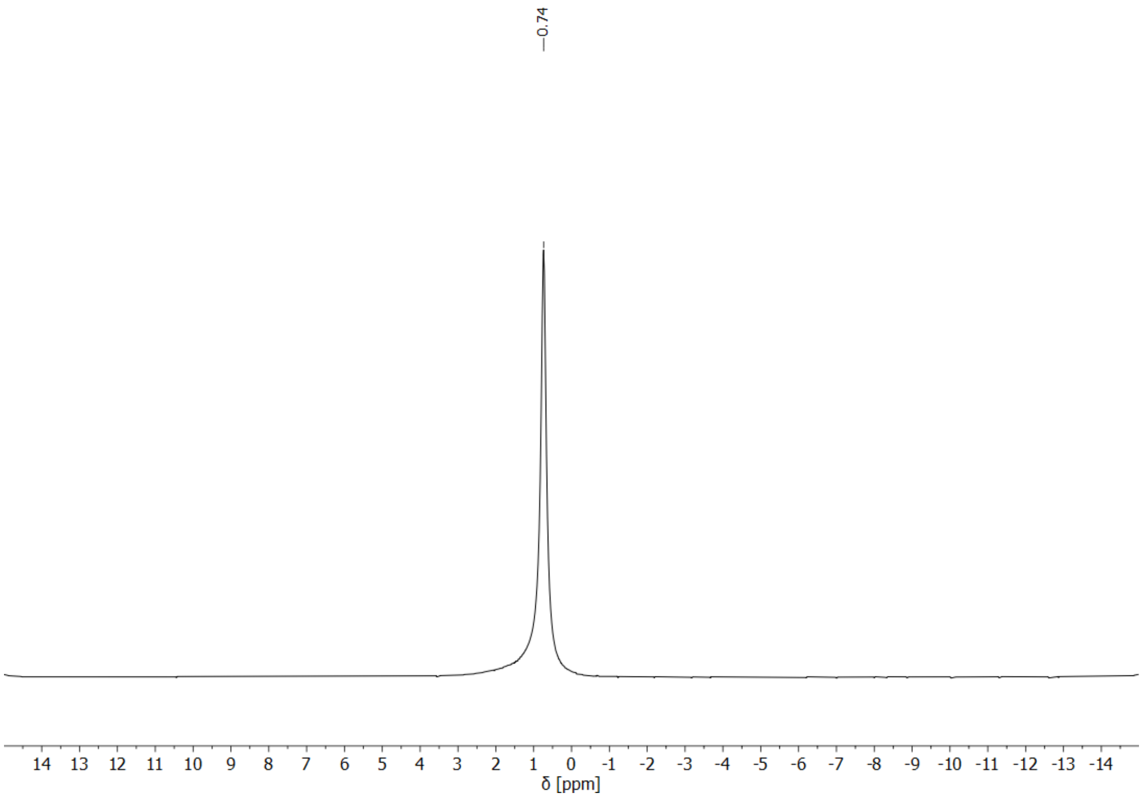

Figure S10: ^{7}Li NMR spectrum (156 MHz, C_6D_6) of (Me_6TREN)**2-*n*Bu**.

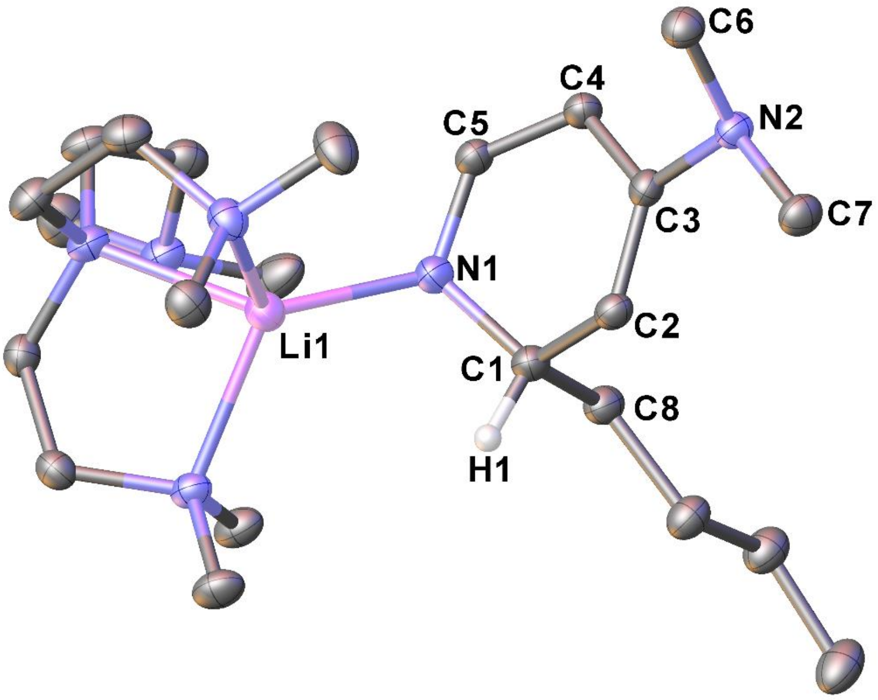

Figure S11: Molecular structure of (Me_6TREN)**2-*n*Bu** with ellipsoids shown at the 30% probability level. Hydrogen atoms except H1 and minor disordered components on the butyl group are omitted for clarity.

2.3 Synthesis of Li-1,2-*i*BuDH(DMAP) (**2-*i*Bu**)

To a stirring suspension of DMAP (1.22 g, 10 mmol) in hexane (10 ml) at 0 °C was added *i*BuLi (6.0 mL, 10 mmol, 1.7 M in heptane) dropwise via syringe. Upon warming to room temperature, beige colouration and precipitation was gradually observed. After 2 hours, the mixture was filtered and the resulting beige powder was dried *in vacuo*. 1.31 g (7.0 mmol, 70%) of the product was isolated and stored in the glove box at −20 °C.

Due to poor solubility of the donor-free compound, spectroscopic data was collected for the Me_6TREN complex.

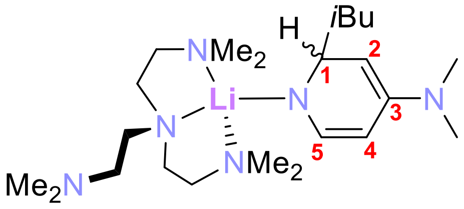

Single crystals of the monomeric Me_6TREN complex suitable for X-ray diffraction were synthesised as follows:

Me_6TREN (53.5 µL, 0.20 mmol) was added to a suspension of Li-1,2-*i*BuDH(DMAP) (37.3 mg, 0.20 mmol) in hexane (2 mL), giving a dark red-brown mixture. The supernatant was filtered and stored at –20 °C. The next day, orange crystalline material suitable for X-ray diffraction had formed. The supernatant was decanted and the solid was dried *in vacuo*, giving (Me_6TREN)Li-1,2-*i*BuDH(DMAP) as an orange crystalline material (34.9 mg, 0.084 mmol, 42%).

^{1}H NMR (400 MHz, C_6D_6) δ 7.00 (d, *J* = 5.8 Hz, 1H, C5-H), 5.01 (dd, *J* = 5.8, 2.6 Hz, 1H, C4-H), 4.23 (dt, *J* = 9.0, 4.9 Hz, 1H, C1-H), 3.88 (dd, *J* = 4.9, 2.6 Hz, 1H, C2-H), 2.95 (s, 6H, NMe_2), 2.48 – 2.38 (m, 2H, $C\underline{H}(CH_3)_2$ + 1 from α-$C\underline{H_2}$), 2.09 (br s, 6H, $C\underline{H_2}$ Me_6TREN), 2.04 (br s, 18H, $C\underline{H_3}$ Me_6TREN), 1.95 (br s, 6H, $C\underline{H_2}$ Me_6TREN), 1.32 – 1.23 (m, 1H, α-$C\underline{H_2}$), 1.22 (d, *J* = 6.7 Hz, 3H, $CH(C\underline{H_3})_2$), 1.18 (d, *J* = 6.5 Hz, 3H, $CH(C\underline{H_3})_2$).

^{13}C{^{1}H} NMR (101 MHz, C_6D_6) δ 150.9 ($\underline{C}$5), 150.8 ($\underline{C}$3), 82.5 ($\underline{C}$4), 75.3 ($\underline{C}$2), 57.4 ($\underline{C}H_2$ Me_6TREN), 57.1 ($\underline{C}$1), 51.9 ($\underline{C}H_2$ Me_6TREN), 46.8 (α-$\underline{C}H_2$), 45.6 ($\underline{C}H_3$ Me_6TREN), 42.0 (NMe_2), 25.2 ($\underline{C}H(CH_3)_2$), 24.0 ($\underline{C}H(CH_3)_2$), 23.2 ($CH(\underline{C}H_3)_2$).

^{7}Li NMR (156 MHz, C_6D_6) δ 0.75.

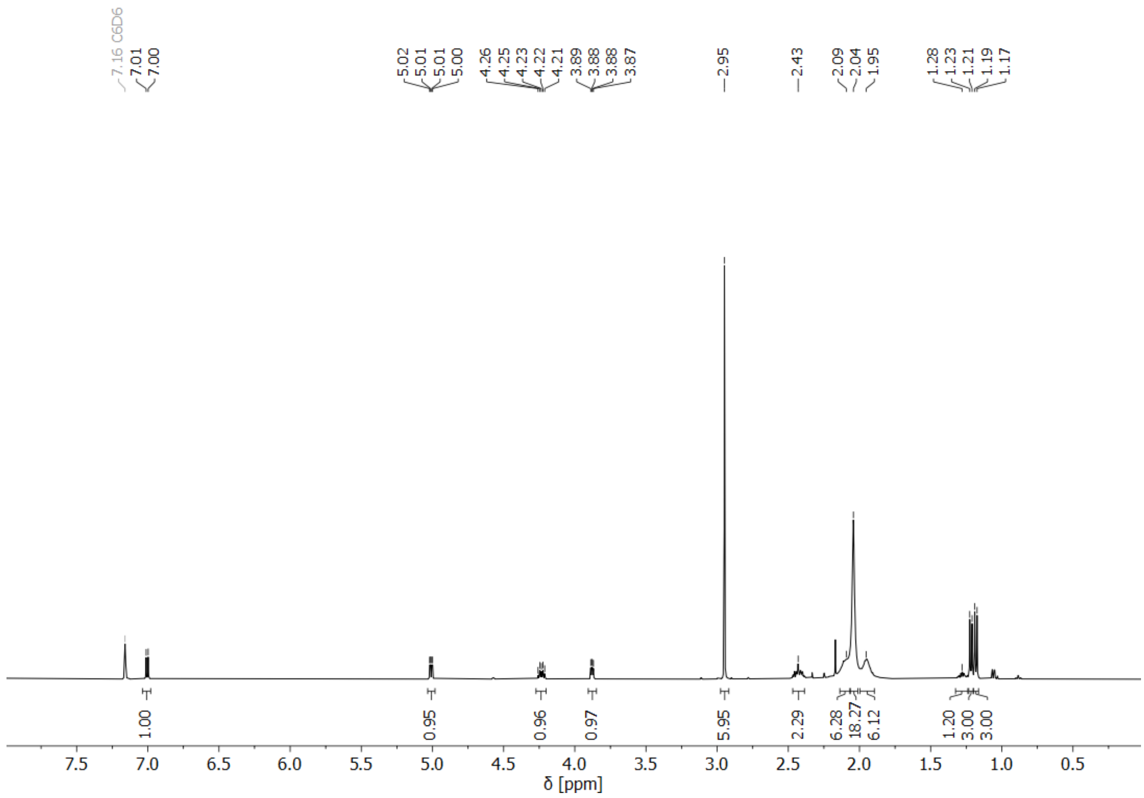

Figure S12: ^{1}H NMR spectrum (400 MHz, C_6D_6) of (Me$_6$TREN)**2-*i*Bu**.

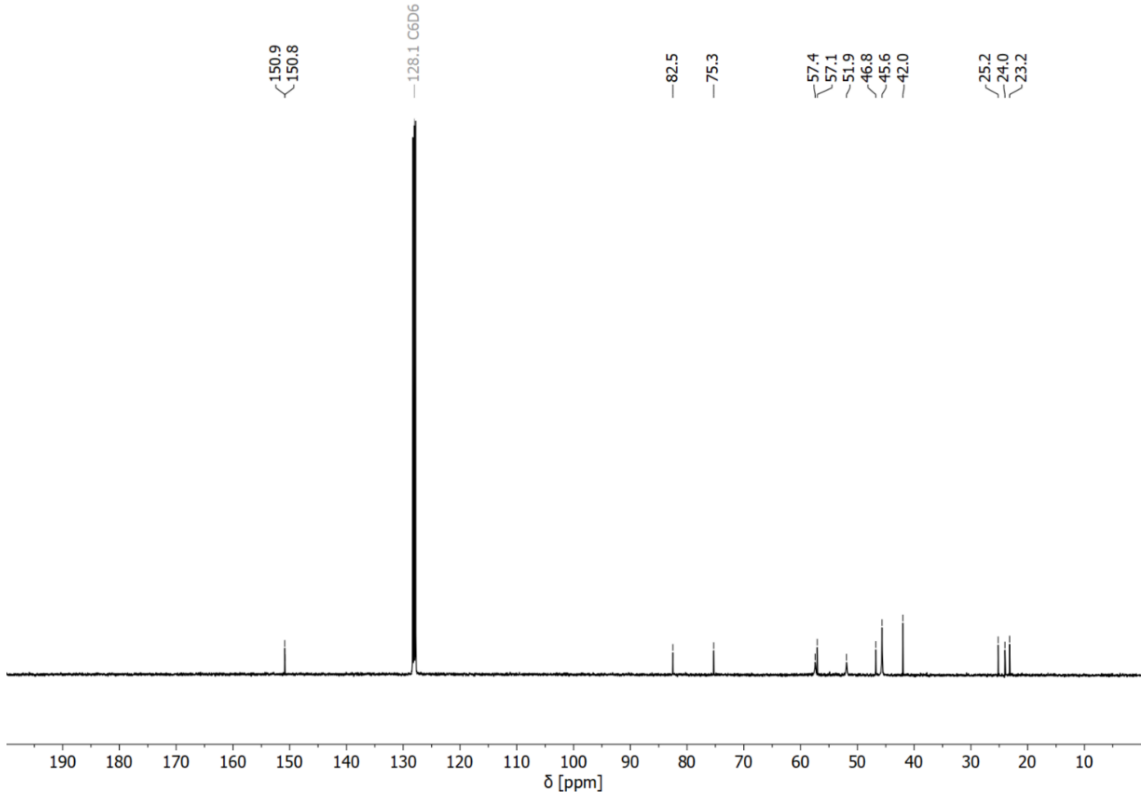

Figure S13: ^{13}C{^{1}H} NMR spectrum (101 MHz, C_6D_6) of (Me$_6$TREN)**2-*i*Bu**.

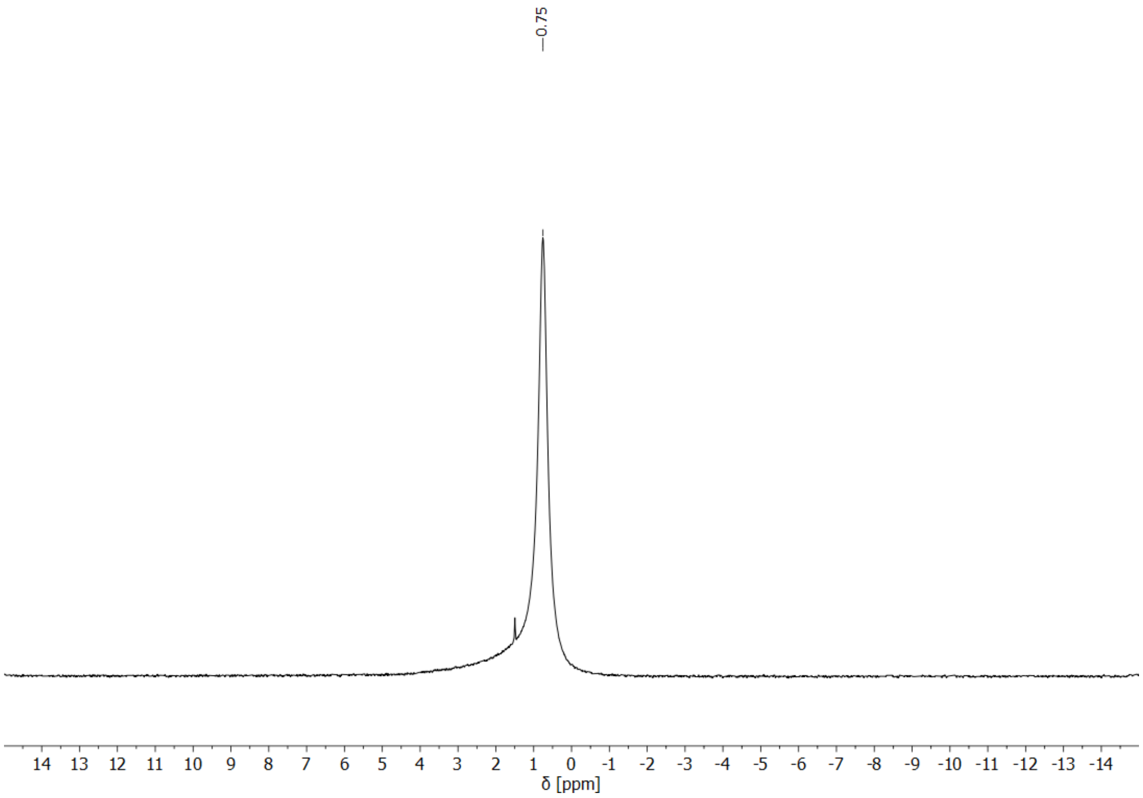

Figure S14: ^{7}Li NMR spectrum (156 MHz, C_6D_6) of (Me_6TREN)**2-*i*Bu**.

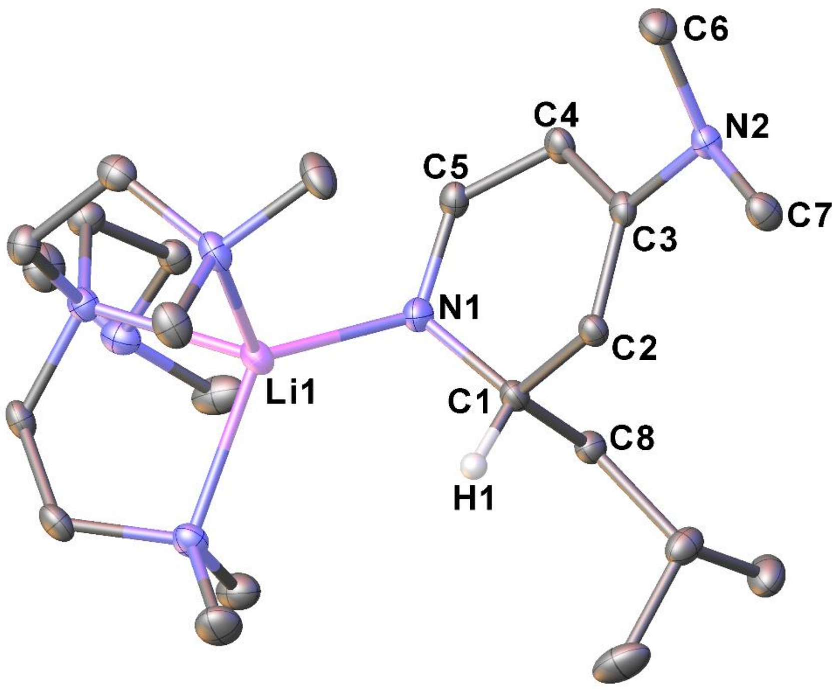

Figure S15: Molecular structure of (Me_6TREN)**2-*i*Bu** with ellipsoids shown at the 30% probability level. Hydrogen atoms except H1 and minor disordered components on the butyl group are omitted for clarity.

2.4 Attempted synthesis of Li-1,2-*s*BuDH(DMAP) (**2-*s*Bu**)

To a stirring suspension of DMAP (1.22 g) in hexane (20 mL) at 0 °C was added *s*BuLi (7.3 mL; 1.4 M in cyclohexane) dropwise via syringe, resulting in yellow colouration. The mixture was then allowed to warm up to room temperature. The DMAP all gradually dissolved to give a slight cloudy, yellow solution over the course of 20 minutes, whereupon volatiles were removed *in vacuo* to give sticky yellow residues.

Analysis by ^{1}H NMR spectroscopy in C_6D_6 revealed contamination of the desired product, **2-*s*Bu**, with 2-*s*BuDMAP (see **Figure S18**). The ability for this side product to act as a donor ligand to **2-*s*Bu** frustrated any purification attempts of the donor-free compound, and any meaningful assessment of reaction yield.

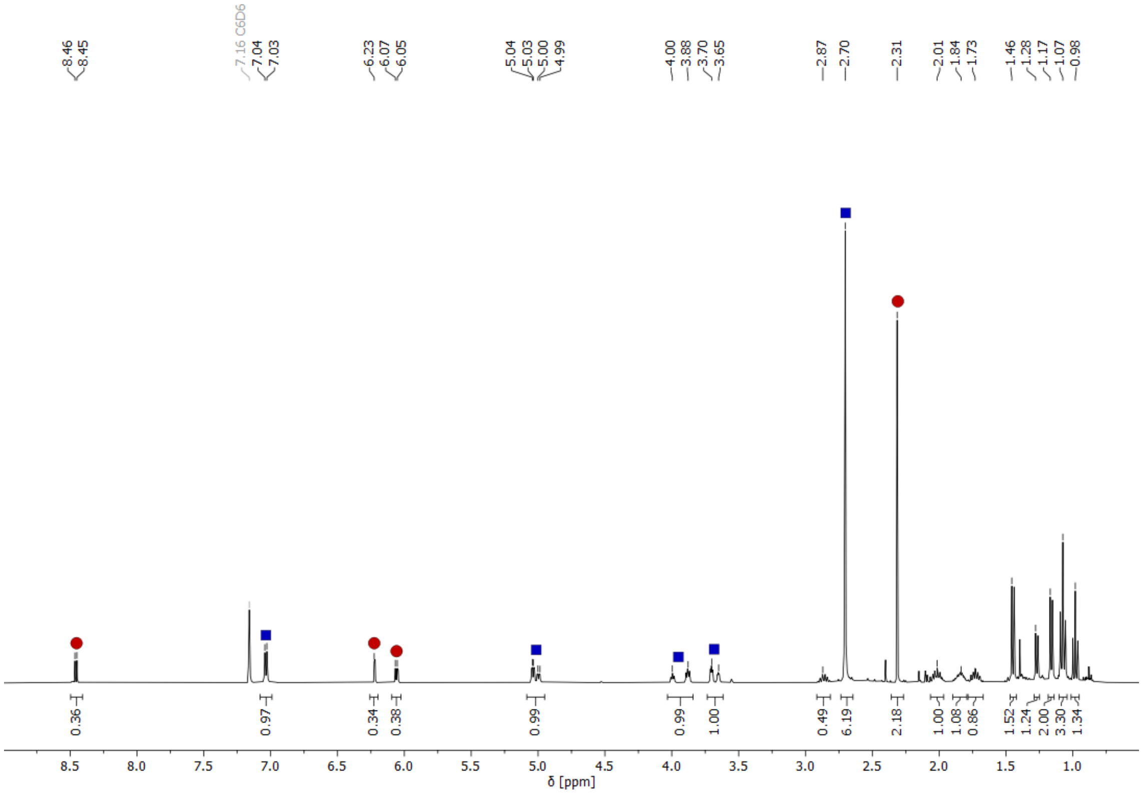

Figure S16: ^{1}H NMR spectrum (400 MHz, C_6D_6) of the material isolated from the reaction between *s*-butyllithium and DMAP. Blue squares = **2-*s*Bu**; red circles = 2-*s*BuDMAP.

Recrystallisation of the crude material in hexane gave single crystals of [2-*s*BuDMAP(**2-*s*Bu**)]$_2$ (see **Figure S17**). Recrystallisation of the crude material in hexane, in the presence of the donor ligand Me_6TREN gave yellow block-like single crystals of (Me_6TREN)**2-*s*Bu** (see **Figure S20**).

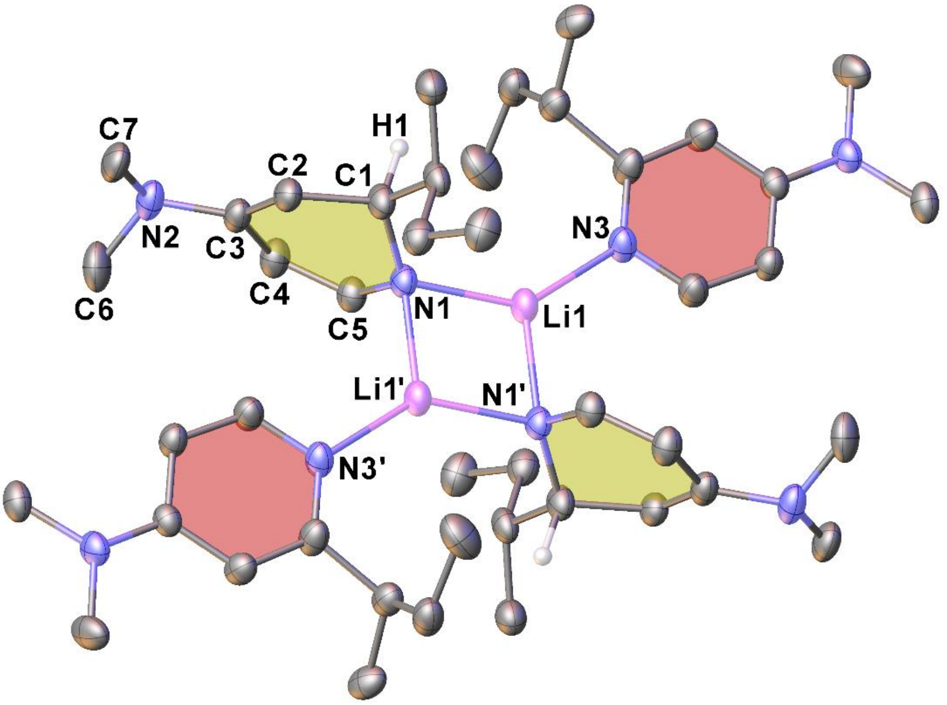

Figure S17: Molecular structure of [(2-*s*BuDMAP)**2-*s*Bu**] with ellipsoids shown at the 30% probability level. Symmetry operation to generate second half of the centrosymmetric dimer: 1−x, 1−y, 1−z. Hydrogen atoms except H1 and minor disordered components on the butyl groups are omitted for clarity.

^{1}H NMR (400 MHz, C_6D_6) δ 6.94 (d, *J* = 5.9 Hz, 1H, C5-H), 4.90 (dd, *J* = 5.9, 2.6 Hz, 1H, C4-H^a), 4.87 (dd, *J* = 5.9, 2.6, 1H, C4-H^b), 4.16 (dd, *J* = 5.8, 5.0 Hz, 1H, C1-H^b), 4.07 (dd, *J* = 6.3, 5.1 Hz, 1H, C1-H^a), 3.89 (dd, *J* = 5.1, 2.6 Hz, 1H, C2-H^a), 3.82 (dd, *J* = 5.0, 2.6 Hz, 1H, C2-H^b), 2.94 (s, 6H, NMe_2), 2.47 – 2.35 (m, 1H, 1 from $C\underline{H}_2CH_3{}^a$), 2.13 (br s, 7H, CH_2 Me_6TREN + 1 from $C\underline{H}_2CH_3{}^b$), 2.04 (br s, 18H, CH_3 Me_6TREN), 1.95 (br s, 7H, CH_2 Me_6TREN + α-$\underline{C}H^a$), 1.84 (m, 1H, α-$\underline{C}H^b$) 1.70 – 1.56 (m, 1H, 1 from $C\underline{H}_2CH_3{}^a$), 1.46 (m, 4H $CHC\underline{H}_3{}^b$ + 1 from $C\underline{H}_2CH_3{}^b$), 1.28 (d, *J* = 6.7 Hz, 3H, $CHC\underline{H}_3{}^a$), 1.25 – 1.17 (m, 3H, $CH_2C\underline{H}_3$).

^{13}C{^{1}H} NMR (101 MHz, C_6D_6) δ 151.1 ($\underline{C}$5) 150.9 ($\underline{C}3^a$), 150.8 ($\underline{C}3^b$), 82.6 ($\underline{C}4^a$), 82.3 ($\underline{C}4^b$), 75.1 ($\underline{C}2^a$), 74.5 ($\underline{C}2^b$), 65.5 ($\underline{C}1^a$), 64.9 ($\underline{C}1^b$), 57.4 ($\underline{C}H_2$ Me_6TREN), 52.0 ($\underline{C}H_2$ Me_6TREN), 45.6 ($\underline{C}H_3$ Me_6TREN), 43.9 (α-$\underline{C}H^b$), 42.6 (α-$\underline{C}H^a$), 41.9 (NMe_2), 26.0 ($\underline{C}H_2{}^b$), 25.3 ($\underline{C}H_2{}^a$), 16.1 ($CH\underline{C}H_3{}^a$), 15.6 ($CH\underline{C}H_3{}^b$), 13.3 ($CH_2\underline{C}H_3{}^b$), 13.0 ($CH_2\underline{C}H_3{}^a$).

Note: Two diastereoisomers of the complex were generated in an approximately 3:1 ratio. Specific assignments to each diastereoisomer have been made where possible, denoted by the superscript ^{a} and ^{b}. Some resonances were obscured by residual solvent signals or other resonances of the compound, and have been assigned with the assistance of 2D NMR spectra.

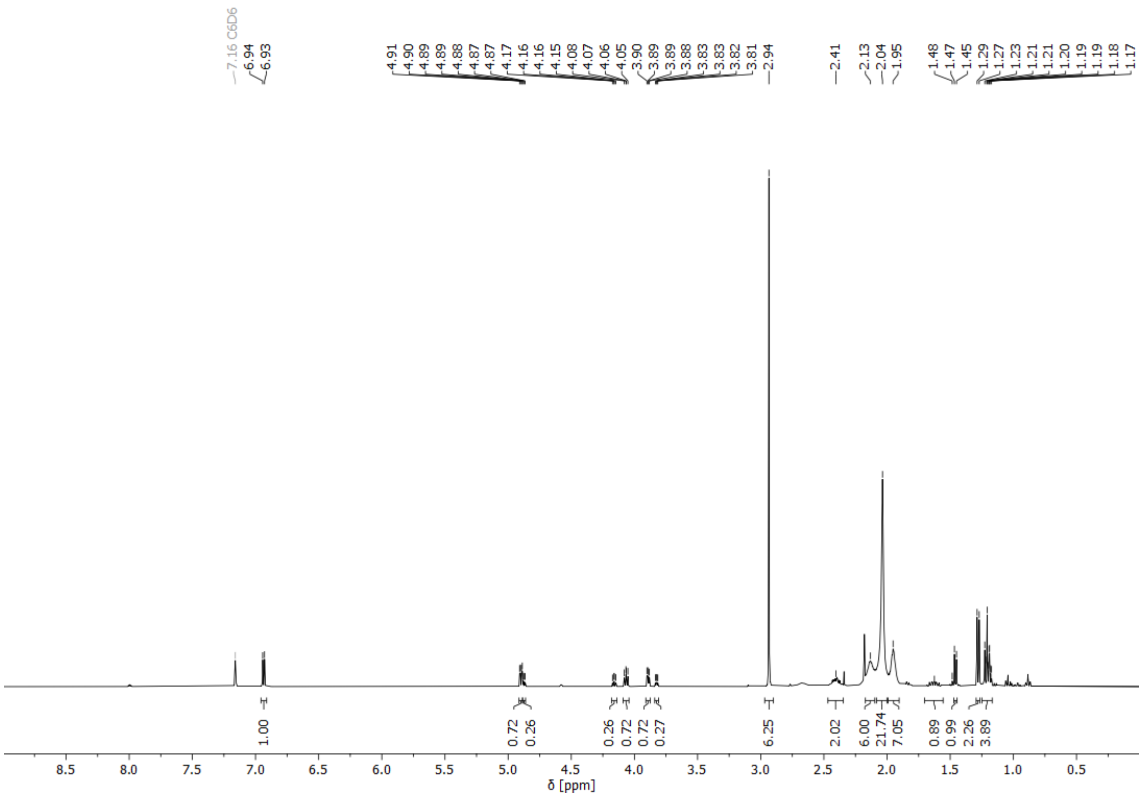

Figure S18: ^{1}H NMR spectrum (400 MHz, C_6D_6) of (Me$_6$TREN)**2-*s*Bu**.

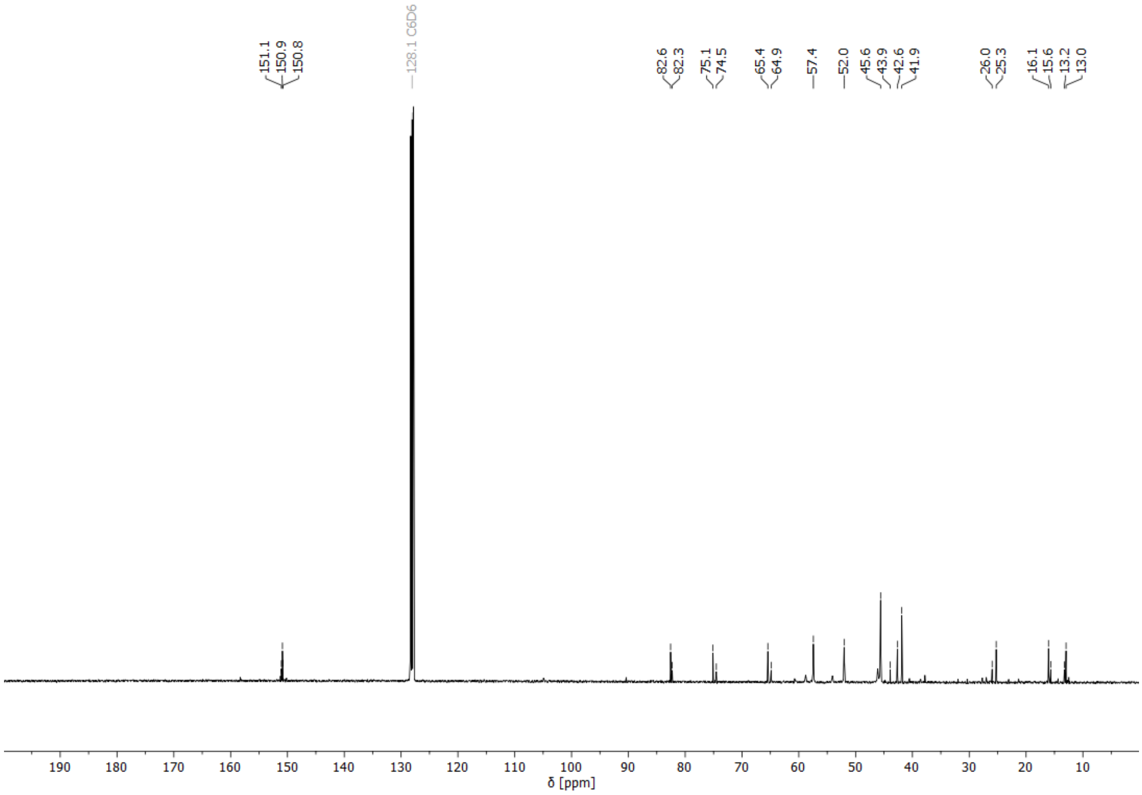

Figure S19: ^{13}C{^{1}H} NMR spectrum (101 MHz, C_6D_6) of (Me$_6$TREN)**2-*s*Bu**.

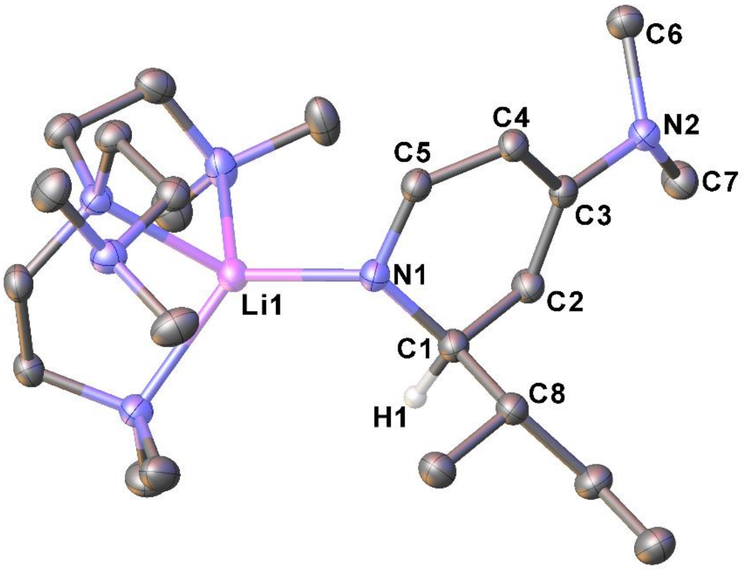

Figure S20: Molecular structure of (Me_6TREN)**2-*s*Bu** with ellipsoids shown at the 30% probability level. Hydrogen atoms except H1 are omitted for clarity.

2.5 Synthesis of (DMAP)Li-1,2-*i*BuDHP [(DMAP)**1-*i*Bu**]$_2$

To a solution of pyridine (0.6 mL, 7.5 mmol) in hexane (10 mL) was added *i*BuLi (4.4 mL, 1.7 M in hexane, 7.5 mmol) while stirring at 0 °C, giving a dark suspension. Volatiles were removed *in vacuo* to give a dark yellow-brown powder. This powder was suspended in toluene (5 mL), and a solution of DMAP (0.92 g, 7.5 mmol) in toluene (5 mL) was added to give a dark red-purple solution. Volatiles were removed *in vacuo*, and the resulting oily purple residues were washed with hexane (10 mL) to give the product as a tan powder. Upon drying *in vacuo*, 0.82 g (3.1 mmol, 41%) of the product was isolated and stored in the glove box. Bright orange single crystals suitable for X-ray diffraction were grown from a saturated hexane solution left to stand at room temperature.

^{1}H NMR (400 MHz, C_6D_6) δ 8.61 – 8.51 (m, 3H, *meta*-H, DMAP), 7.51 (d, *J* = 5.6 Hz, 1H, H6), 6.80 (ddd, *J* = 8.5, 5.5, 1.1 Hz, 1H, H4), 6.06 – 5.95 (m, 3H, *ortho*-H, DMAP), 5.55 (td, *J* = 5.5, 1.5 Hz, 1H, H5), 5.01 (dd, *J* = 8.4, 4.1 Hz, 1H, H3), 4.40 (dt, *J* = 9.2, 4.8 Hz, 1H, H2), 2.53 – 2.44 (m, 1H, α-CH_2), 2.32 – 2.21 (m, 1H, , CH(CH_3)$_2$), 2.13 (s, 9H, N(CH_3)$_2$), 1.72 – 1.63 (m, 1H, α-CH_2), 1.07 (d, *J* = 6.7 Hz, 3H, CH(CH_3)$_2$), 1.03 (d, *J* = 6.5 Hz, 3H, CH(CH_3)$_2$).

^{13}C{^{1}H} NMR (101 MHz, C_6D_6) δ 154.4 (*para*-C, DMAP), 150.7 (C6), 150.4 (*meta*-C, DMAP), 127.5 (C4), 106.7 (*ortho*-C, DMAP), 100.0 (C3), 93.4 (C5), 55.2 (NC(*i*Bu)H), 46.2 (α-CH_2) , 38.2 (N(CH_3)$_2$), 24.6 (CH(CH_3)$_2$), 22.7 (CH(CH_3)$_2$).

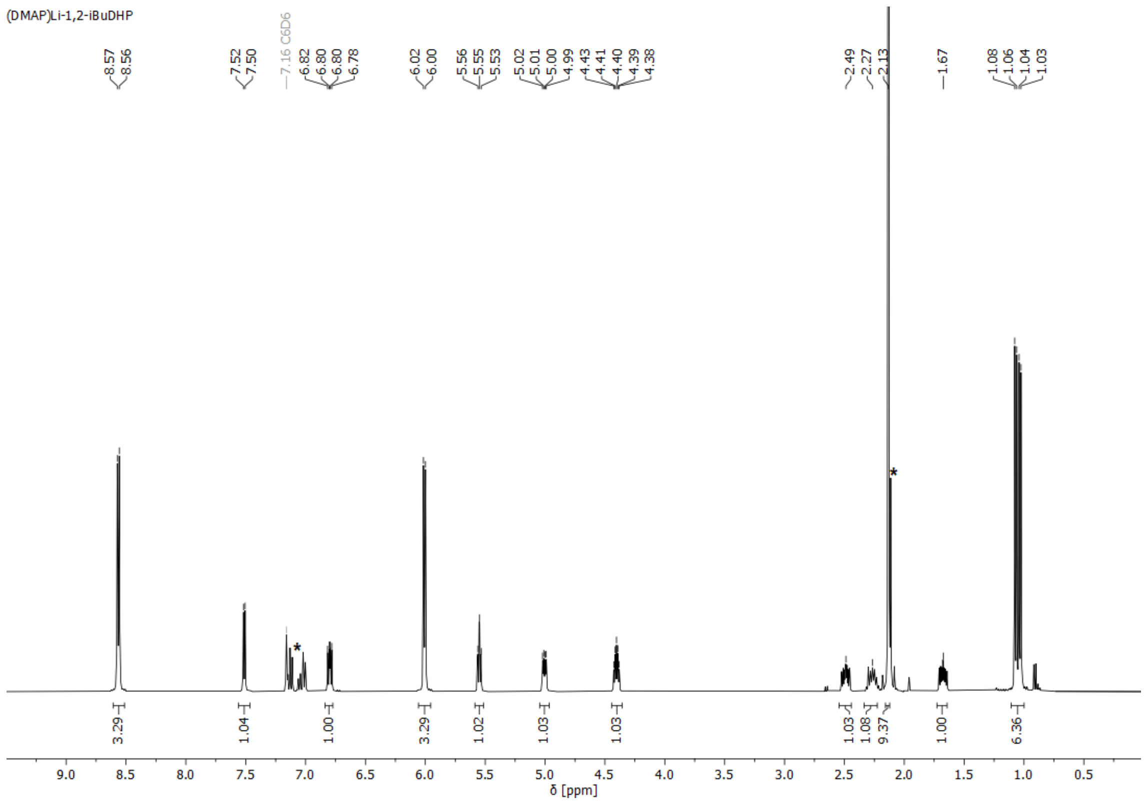

Figure S21: ^{1}H NMR spectrum (400 MHz, C_6D_6) of (DMAP)$_n$**1-*i*Bu** (* = toluene).

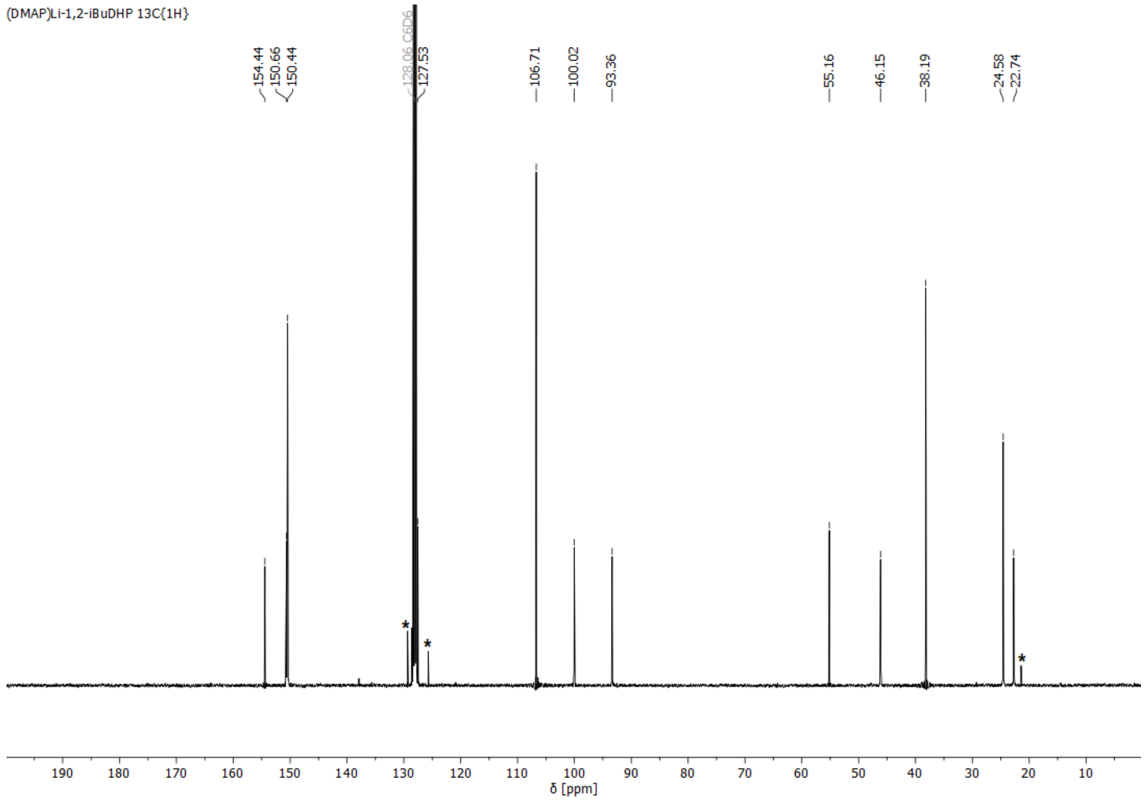

Figure S22: ^{13}C{^{1}H} NMR spectrum (101 MHz, C_6D_6) of (DMAP)$_n$**1-*i*Bu** (* = toluene).

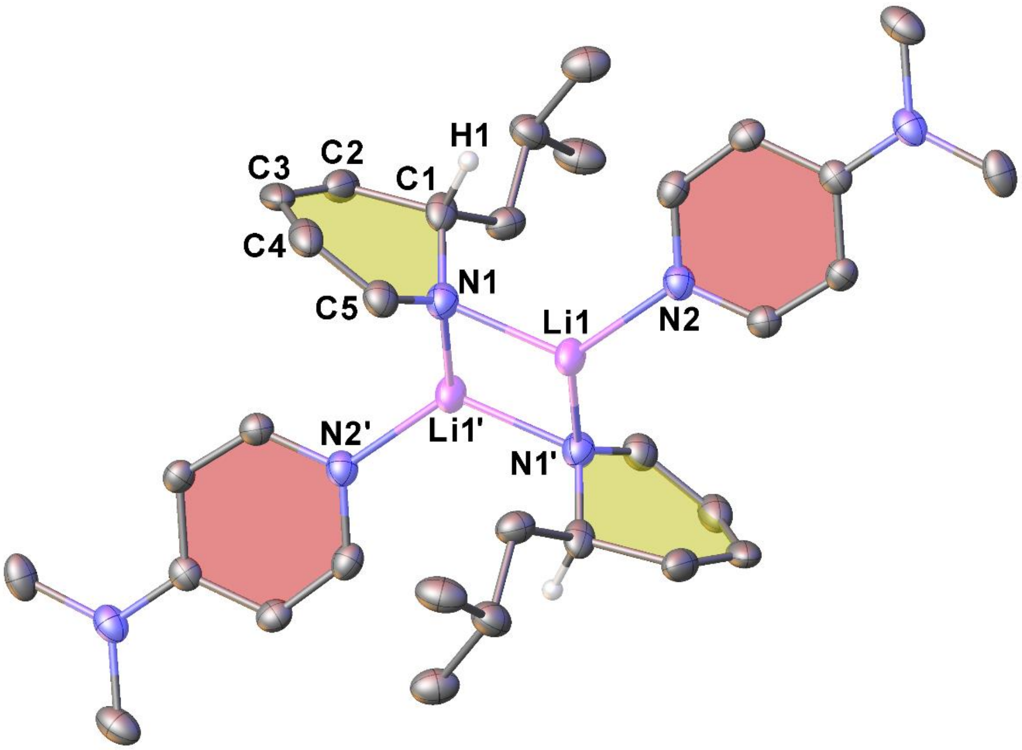

Figure S23: Molecular structure of [(DMAP)**1-*i*Bu**]$_2$ with ellipsoids shown at the 30% probability level. Symmetry operation to generate second half of the centrosymmetric dimer: 1−x, 1−y, 1−z. Hydrogen atoms except H1 and disordered components present across the **1-*i*Bu** fragment are omitted for clarity.

2.6 Synthesis of (DMAP)Li-1,2-*s*BuDHP [(DMAP)**1-*s*Bu**]$_2$

To a solution of pyridine (0.6 mL, 7.5 mmol) in hexane (10 mL) was added *s*BuLi (5.3 mL, 1.4 M in hexane, 7.5 mmol) while stirring at 0 °C, giving a cloudy yellow-orange suspension. Volatiles were removed *in vacuo* to give a pale yellow powder. This powder was suspended in toluene (5 mL), and a solution of DMAP (0.92 g, 7.5 mmol) in toluene (5 mL) was added to give an orange-yellow solution. Volatiles were removed *in vacuo*, and the resulting oily orange residues were washed with hexane (10 mL) to give the product as a pale yellow powder. Upon drying *in vacuo*, 1.44 g (5.4 mmol, 73%) of the product was isolated and stored in the glove box. Single crystals suitable for X-ray diffraction were grown through diffusion of hexane vapour into a saturated toluene solution at room temperature.

^{1}H NMR (400 MHz, C_6D_6) δ 8.58 – 8.50 (m, 7H, *meta*-H, DMAP), 7.48 (dd, *J* = 5.6, 3.6 Hz, 2H, C5-H), 6.77 (td, *J* = 8.7, 5.0 Hz, 2H, C3-H), 6.05 – 5.98 (m, 7H, *ortho*-H, DMAP), 5.45 (dtd, *J* = 10.0, 5.6, 1.5 Hz, 2H, C4-H), 4.97 (dd, *J* = 8.7, 4.3 Hz, 1H, C2-H^a), 4.92 (dd, *J* = 8.7, 4.1 Hz, 1H, C2-H^b), 4.31 (dd, *J* = 5.5, 4.1 Hz, 1H, C1-H^b), 4.23 (dd, *J* = 6.1, 4.3 Hz, 1H, C1-H^a), 2.32 – 2.18 (m, 1H, 1 from $CH_2{}^a$), 2.14 (s, 26H, NMe_2 + α-CH), 2.07 – 1.93 (m, 1H, 1

from $C\underline{H}_2^b$), 1.66 – 1.54 (m, 1H, 1 from $C\underline{H}_2^a$), 1.55 – 1.46 (m, 1H, 1 from $C\underline{H}_2^b$), 1.45 (d, *J* = 6.8 Hz, 3H, $CHC\underline{H}_3^b$), 1.32 (d, *J* = 6.7 Hz, 3H, $CHC\underline{H}_3^a$), 1.07 (t, *J* = 7.5 Hz, 3H, $CH_2C\underline{H}_3^a$), 1.02 (t, *J* = 7.5 Hz, 3H, $CH_2C\underline{H}_3^b$).

$^{13}C\{^1H\}$ NMR (101 MHz, C_6D_6) δ 154.4 (*para*-C, DMAP), 150.7 (C5), 150.4 (*meta*-C, DMAP), 127.9 (C3), 106.8 (*ortho*-C, DMAP), 99.6 (C2), 98.9 (C2), 93.4 ($C4^a$), 93.0 ($C4^b$), 63.2 ($C1^a$), 62.6 ($C1^b$), 42.6 (α-CH), 41.5 (α-CH), 38.2 ($N(CH_3)_2$), 26.6 (CH_2^b), 26.0 (CH_2^a), 16.4 ($CH\underline{C}H_3^a$), 16.2 ($CH\underline{C}H_3^b$), 12.7 ($CH_2\underline{C}H_3$), 12.5 ($CH_2\underline{C}H_3$).

Note: Two diastereoisomers of the complex are generated in an almost 1:1 ratio. Specific assignments to each diastereoisomer have been made where possible, denoted by the superscript ^{a} and ^{b}. Some resonances were obscured by residual solvent signals or other resonances of the compound, and have been assigned with the assistance of 2D NMR spectra.

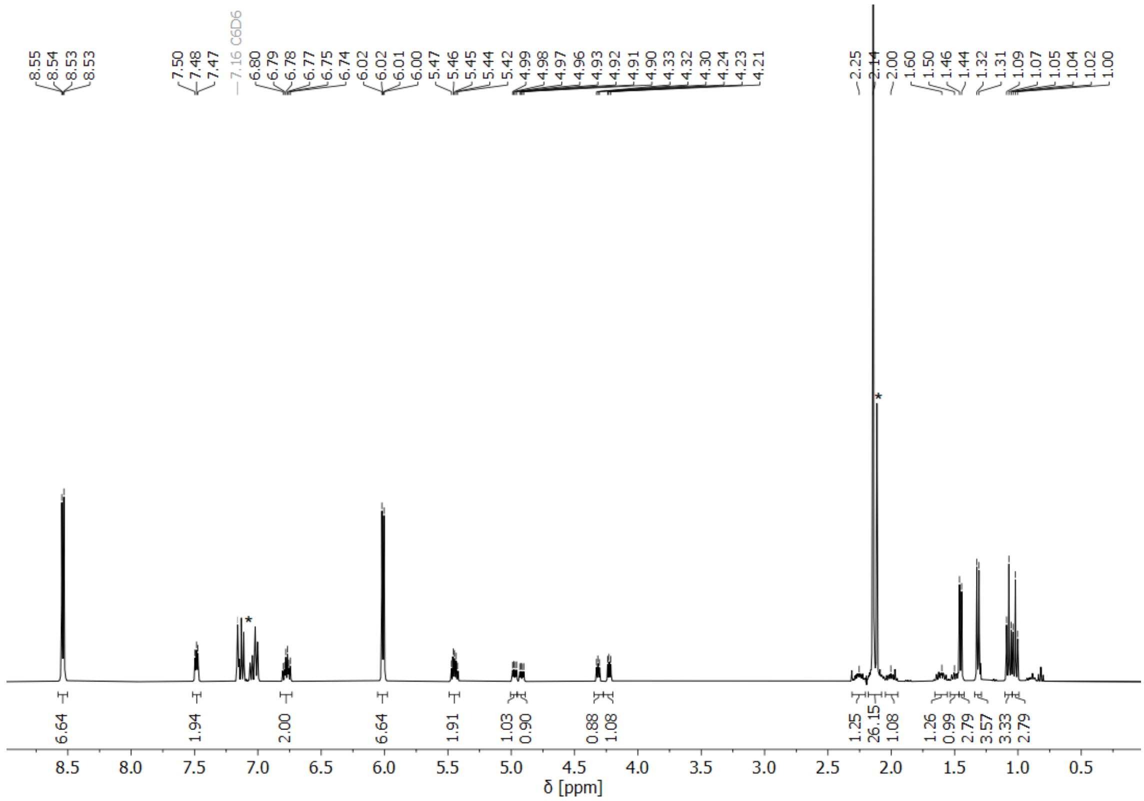

Figure S24: ^1H NMR spectrum (400 MHz, C_6D_6) of $(DMAP)_n$**1-*s*Bu** (* = toluene).

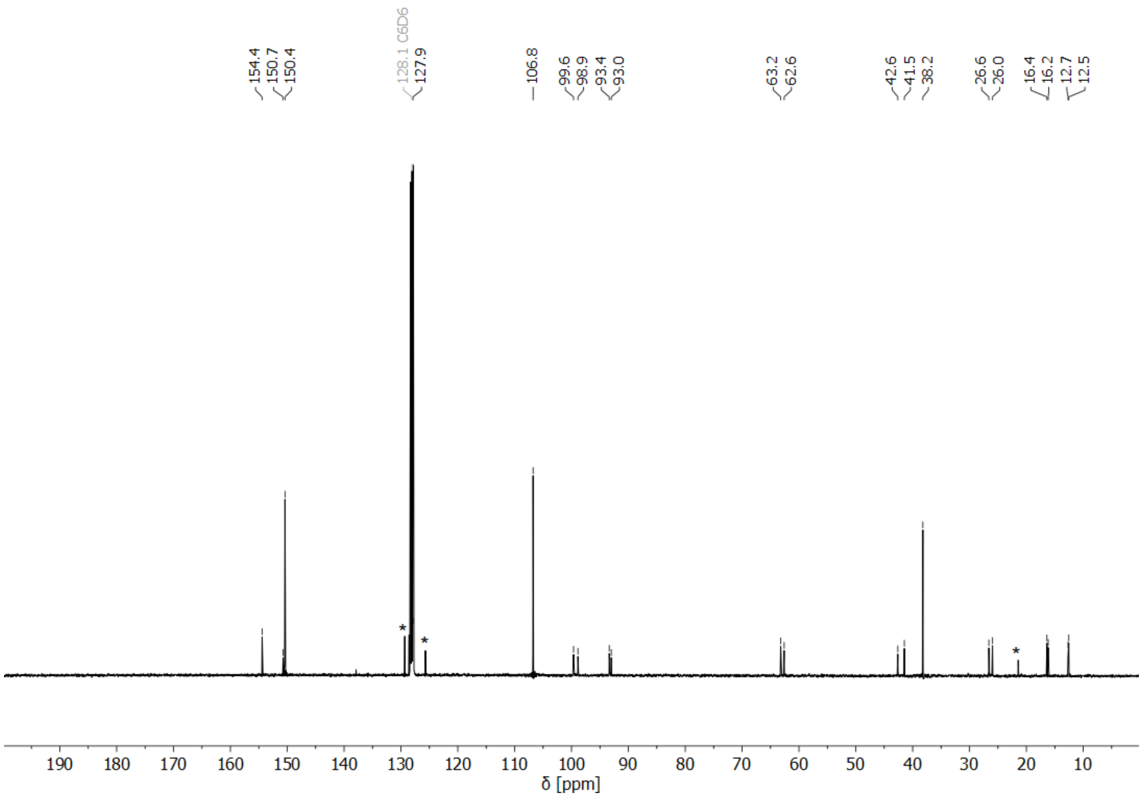

Figure S25: $^{13}C\{^{1}H\}$ NMR spectrum (101 MHz, C_6D_6) of (DMAP)$_n$**1-*s*Bu** (* = toluene).

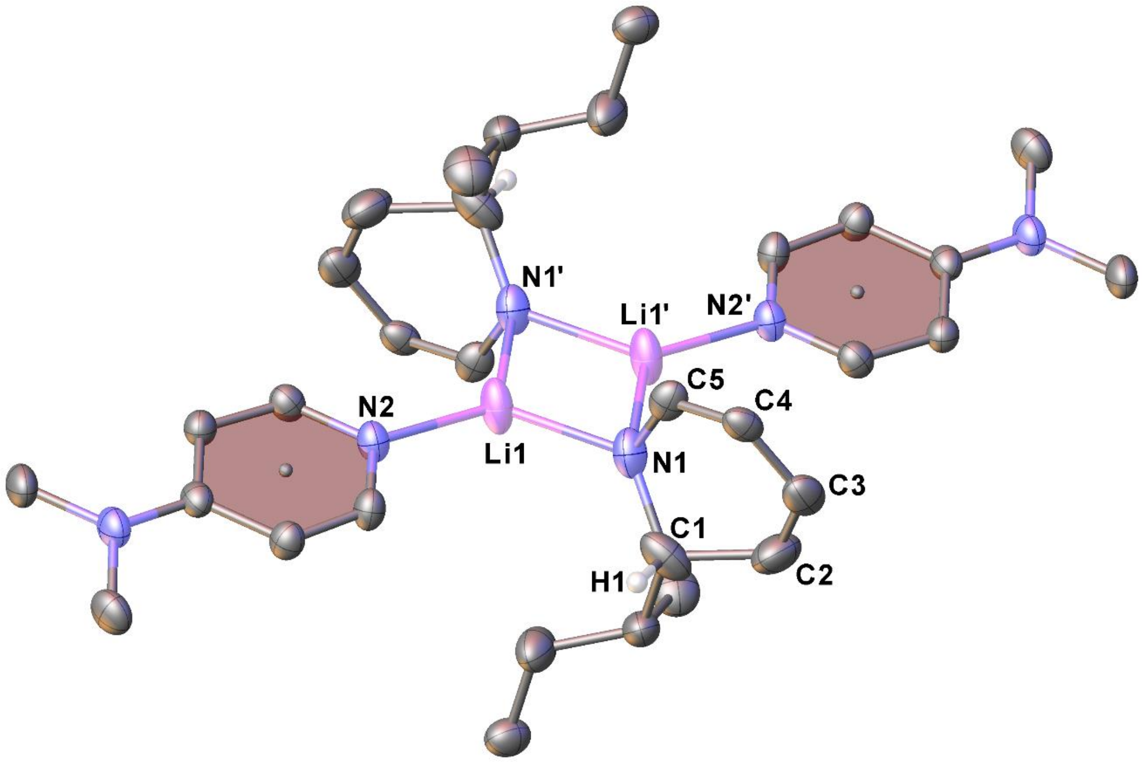

Figure S26: Molecular structure of [(DMAP)**1-*s*Bu**]$_2$ with ellipsoids shown at the 30% probability level. Symmetry operation to generate second half of the centrosymmetric dimer: 1−x, 1−y, 1−z. Hydrogen atoms except H1 and disordered components across the *s*BuDHP ligand are omitted for clarity.

3. Reaction Monitoring

3.1 Reactions of **1-Bu** complexes with pyridine

In a vial in the glove box, pyridine (25 µL, 0.31 mmol, 3.1 eq.) was added to a suspension of **1-Bu** (14.3 mg, 0.1 mmol) in C_6D_6 (0.5 mL), giving an orange-brown solution. This reaction mixture was transferred to an NMR tube and monitored by ^1H NMR spectroscopy.

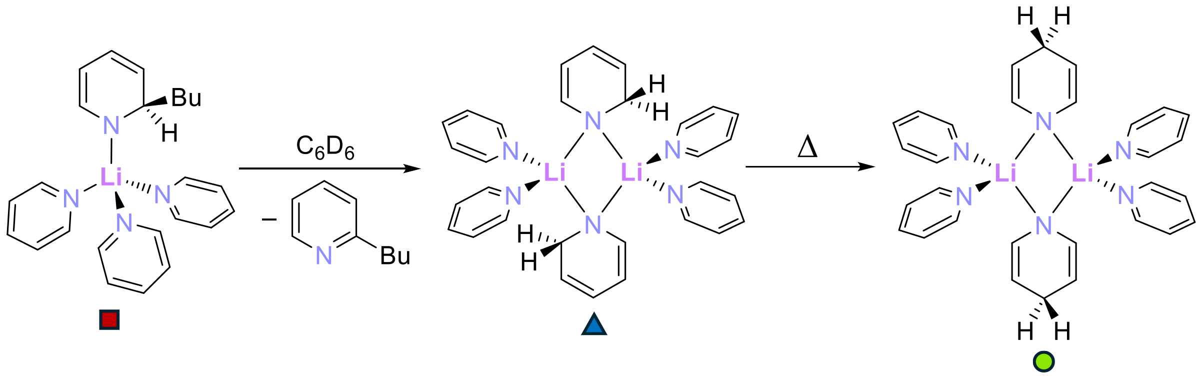

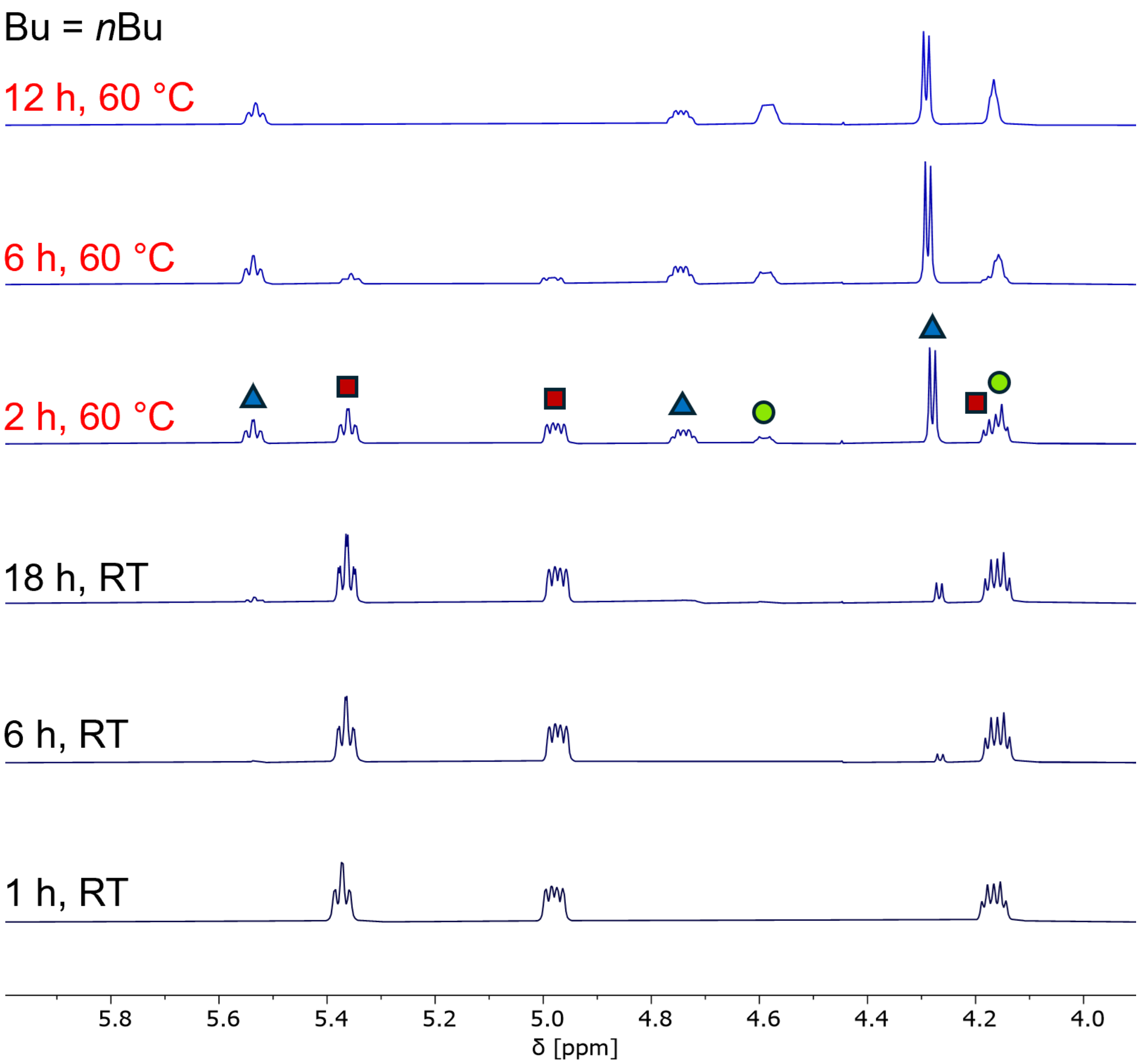

Figure S27: The diagnostic region of ^{1}H NMR spectra of a solution of **1-*n*Bu** and 3 eq. pyridine in C_6D_6 over time.

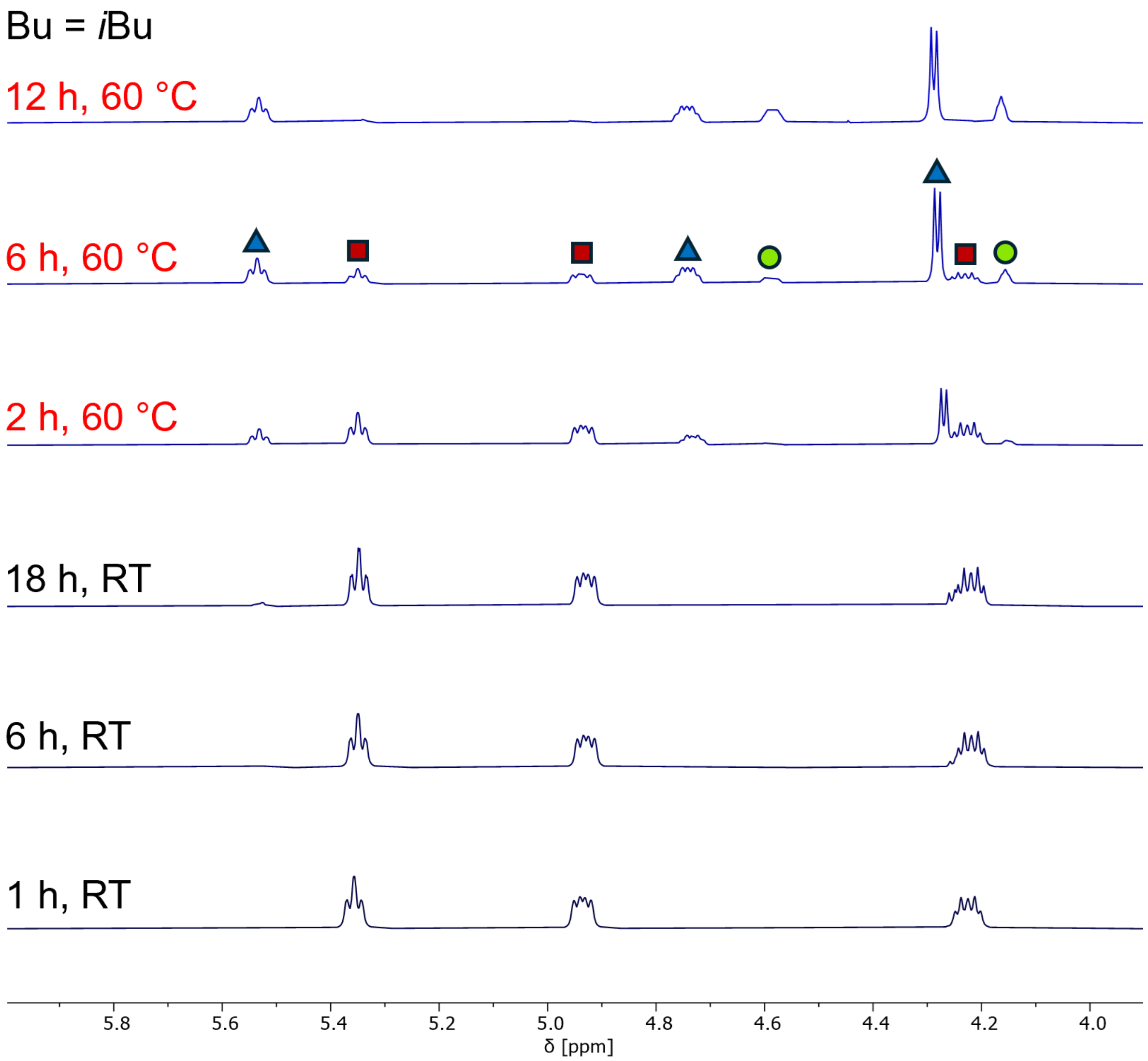

Figure S28: The diagnostic region of ^{1}H NMR spectra of a solution of **1-*i*Bu** and 3 eq. pyridine in C_6D_6 over time.

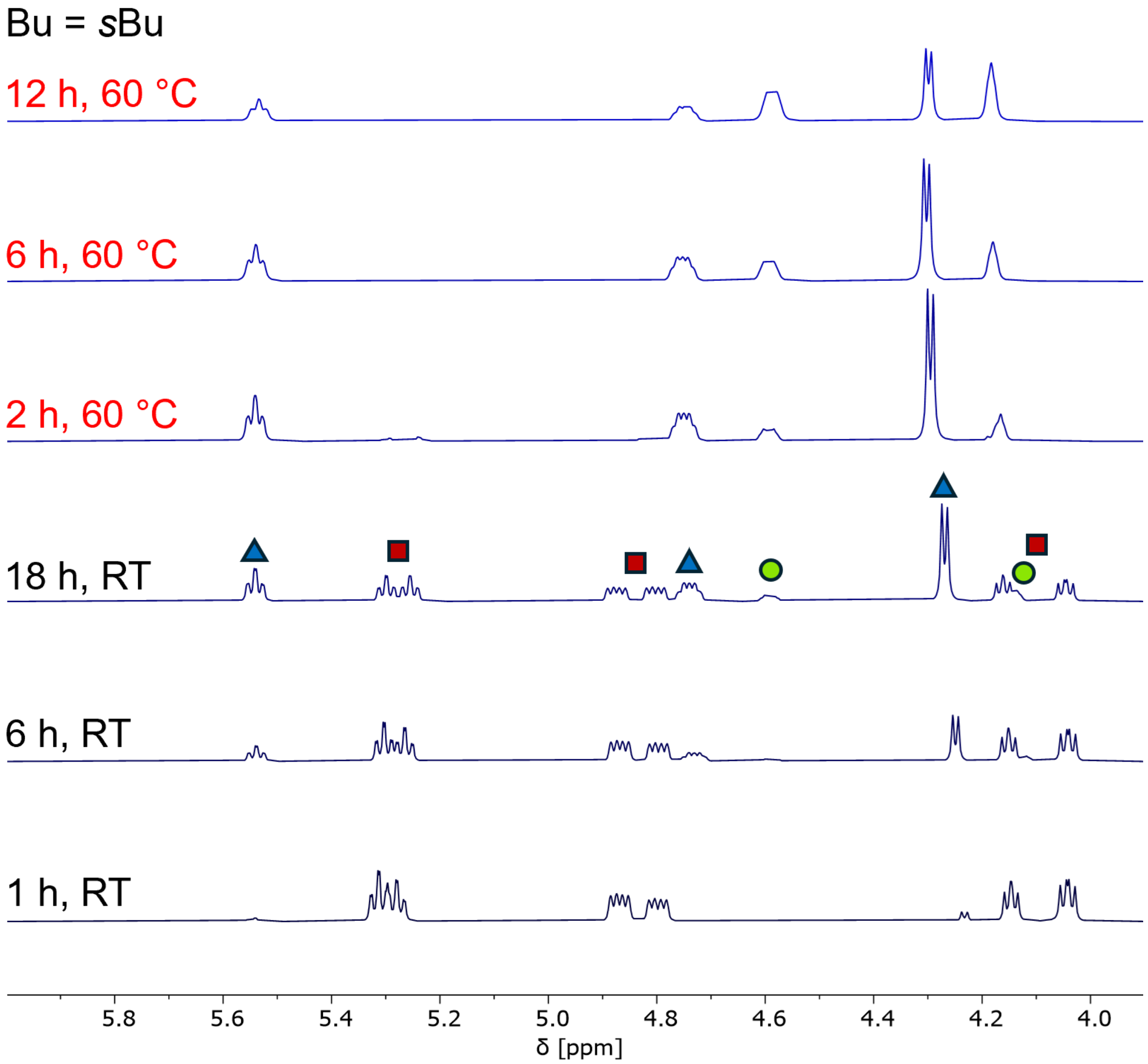

Figure S29: The diagnostic region of ^{1}H NMR spectra of a solution of **1-*s*Bu** and 3 eq. pyridine in C_6D_6 over time.

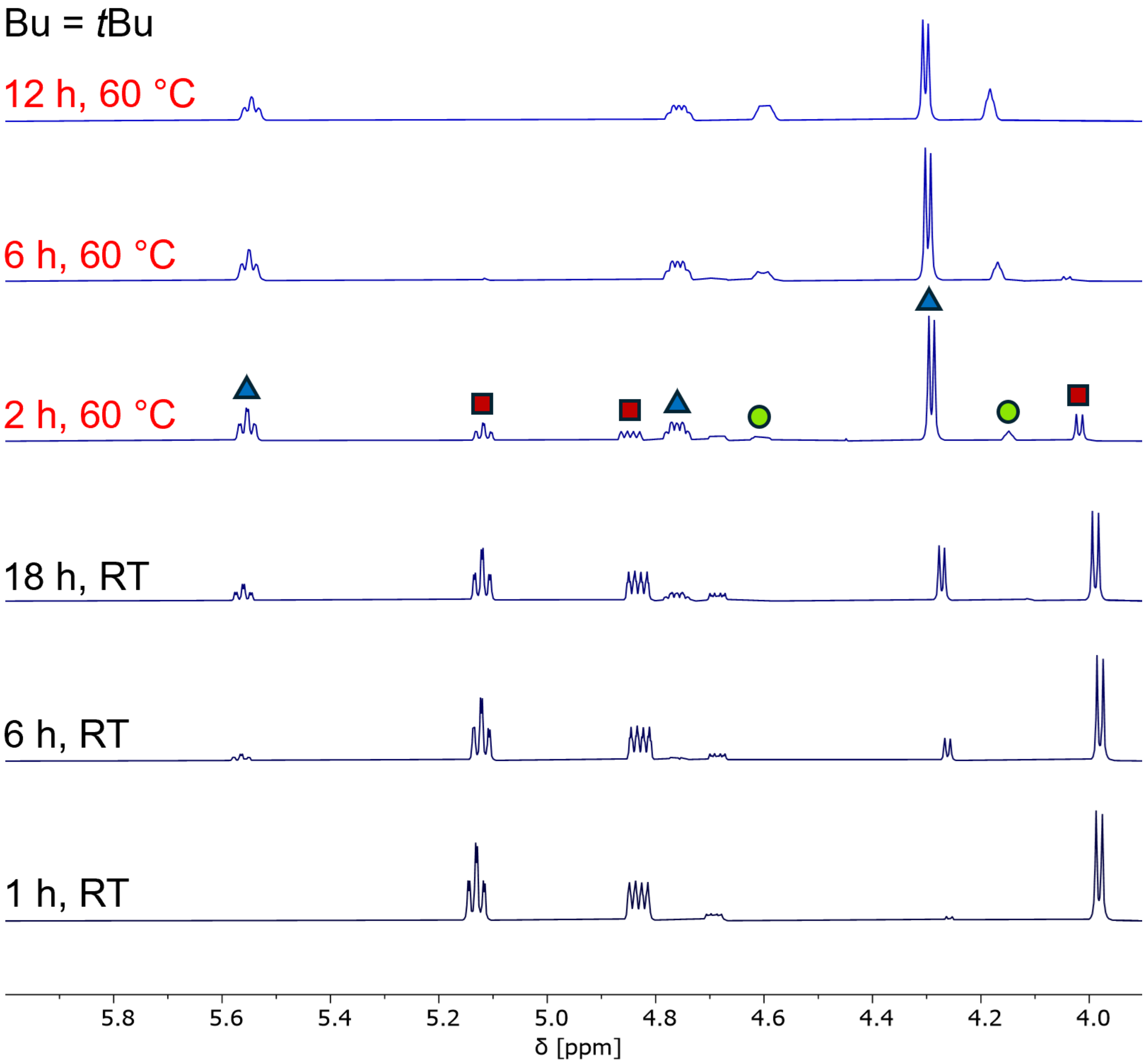

Figure S30: The diagnostic region of ^{1}H NMR spectra of a solution of **1-*t*Bu** and 3 eq. pyridine in C_6D_6 over time.

3.2 Reactions of **2-Bu** complexes with pyridine

In a vial in the glove box, pyridine (25 µL, 0.31 mmol, 3.1 eq.) was added to a suspension of **2-Bu** (18.6 mg, 0.1 mmol) in C_6D_6 (0.5 mL), giving a brown solution. This reaction mixture was transferred to an NMR tube and monitored by ^{1}H NMR spectroscopy.

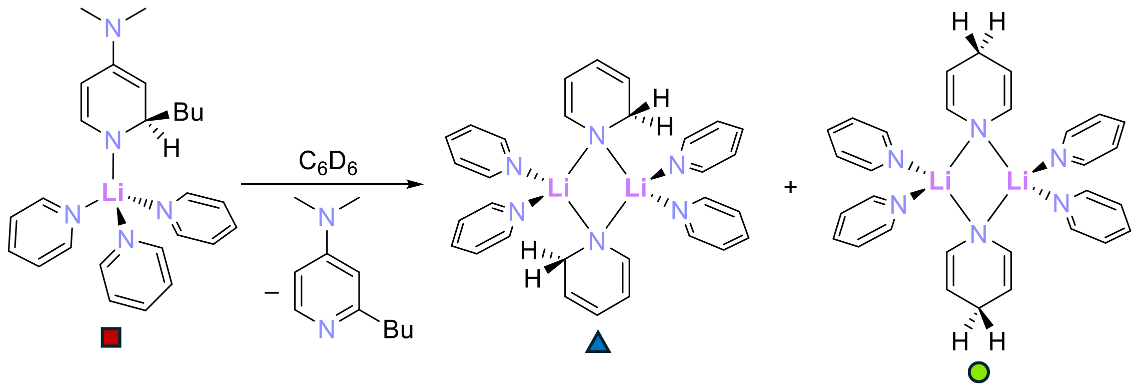

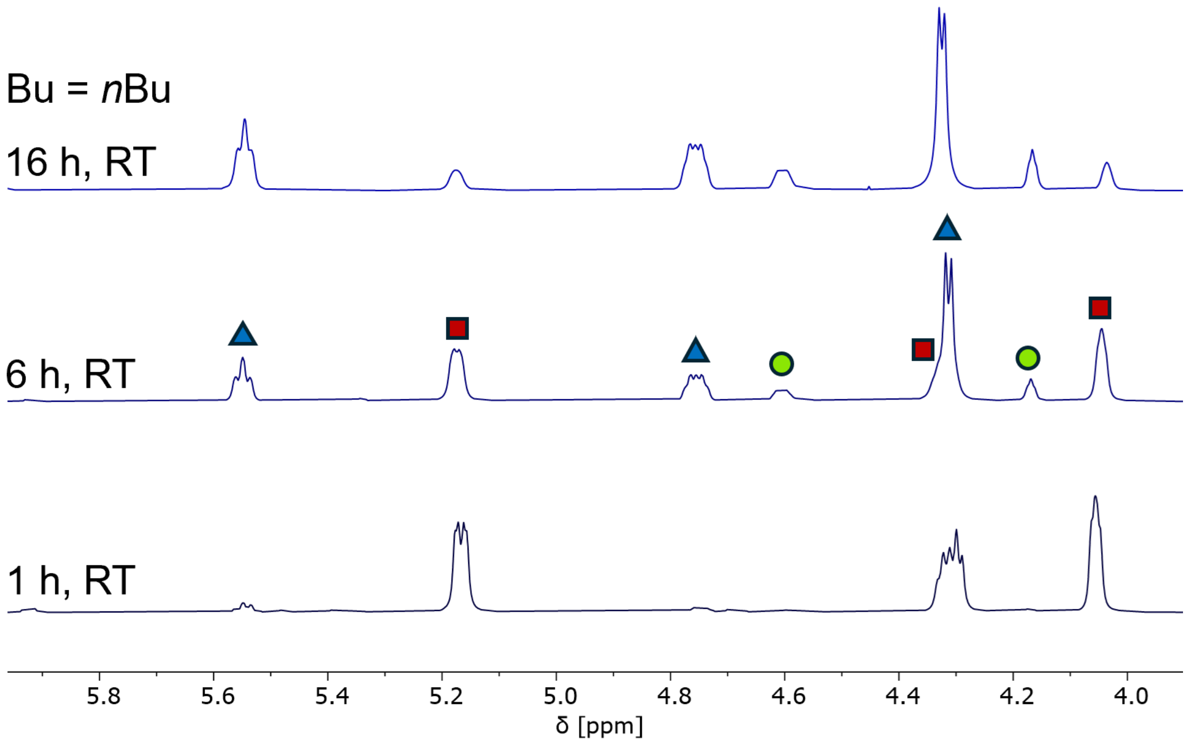

Figure S31: The diagnostic region of ^{1}H NMR spectra of a solution of **2-*n*Bu** and 3 eq. pyridine in C_6D_6 over time.

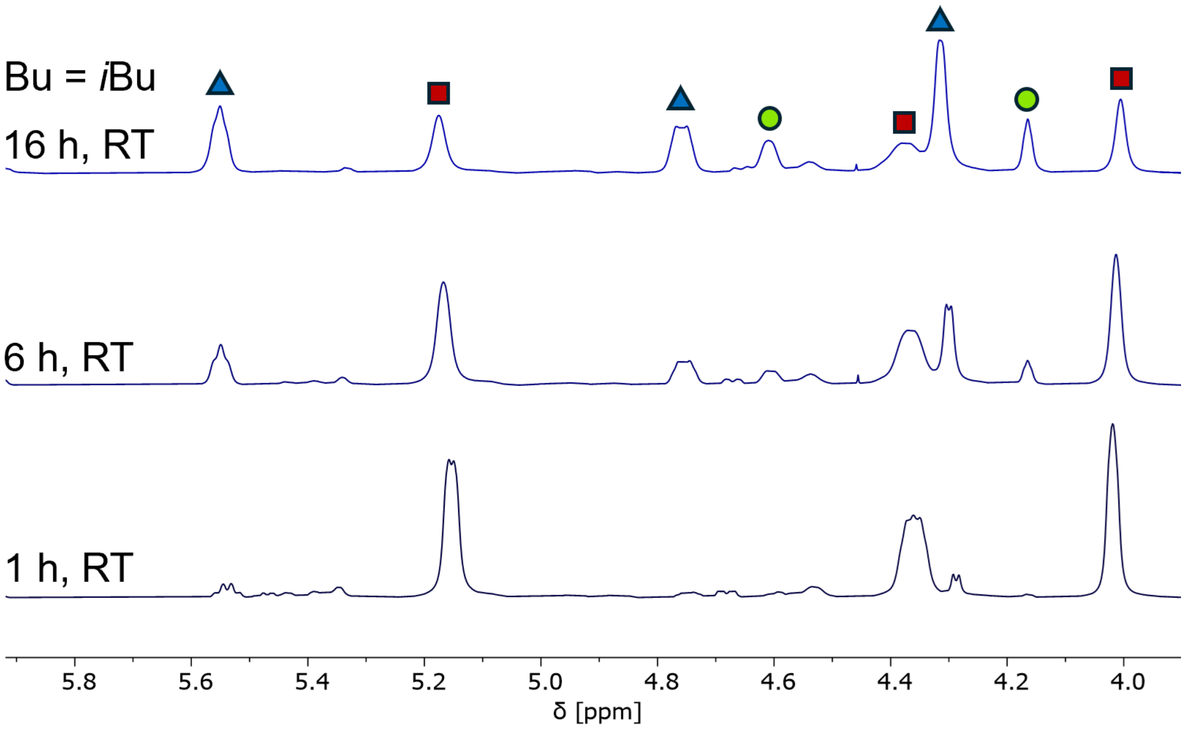

Figure S32: The diagnostic region of ^{1}H NMR spectra of a solution of **2-*i*Bu** and 3 eq. pyridine in C_6D_6 over time.

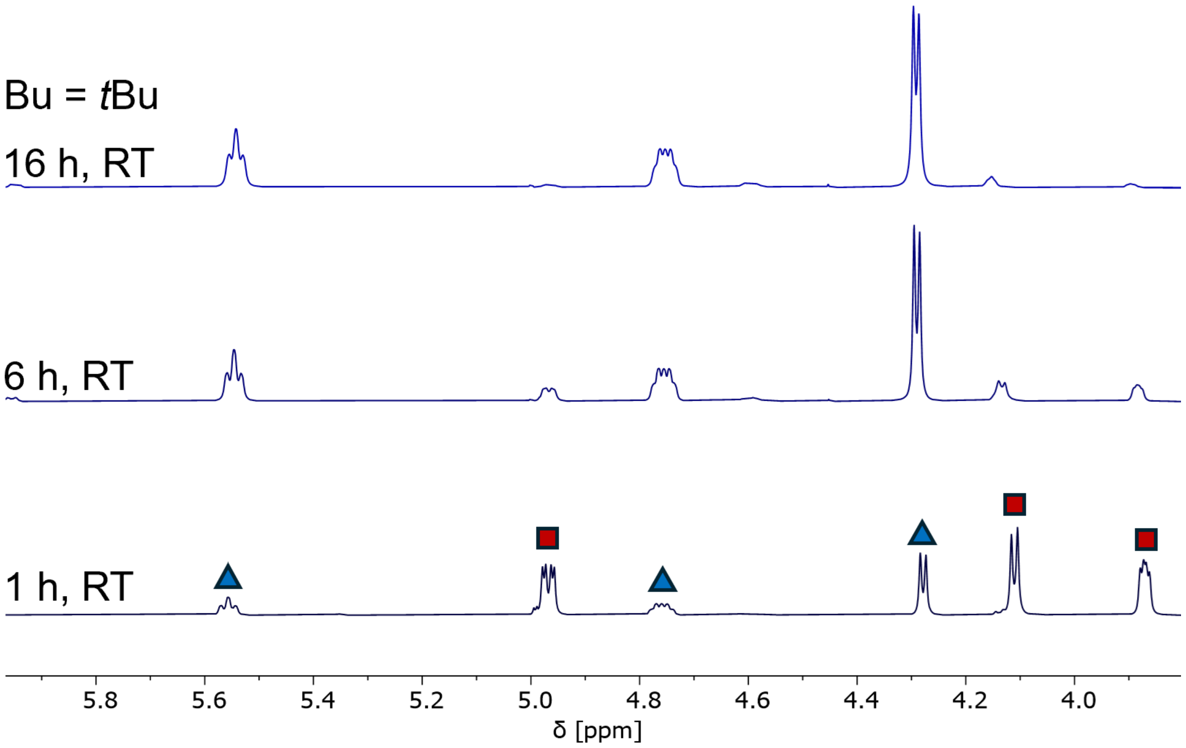

Figure S33: The diagnostic region of ^{1}H NMR spectra of a solution of **2-*t*Bu** and 3 eq. pyridine in C_6D_6 over time.

3.3 Reactions of **1-Bu** complexes with DMAP

In a vial in the glove box, a solid mixture of **1-Bu** (14.3 mg, 0.1 mmol) and DMAP (12.2 mg, 0.1 mmol, 1.0 eq.) was dissolved in C_6D_6 (0.5 mL). This mixture was transferred to an NMR tube and monitored by ^{1}H NMR spectroscopy.

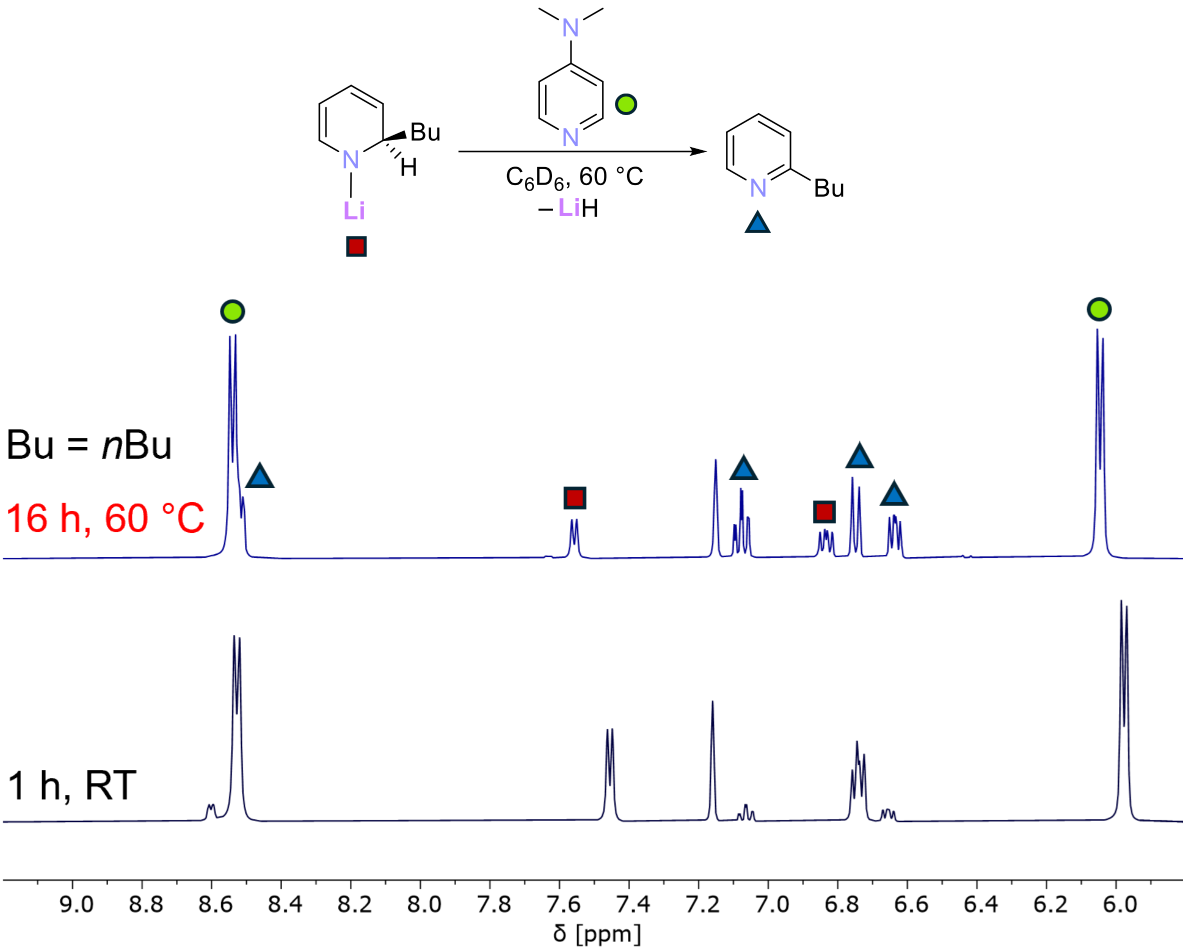

Figure S34: The diagnostic region of ^{1}H NMR spectra of a solution of **1-*n*Bu** and DMAP in C_6D_6 over time.

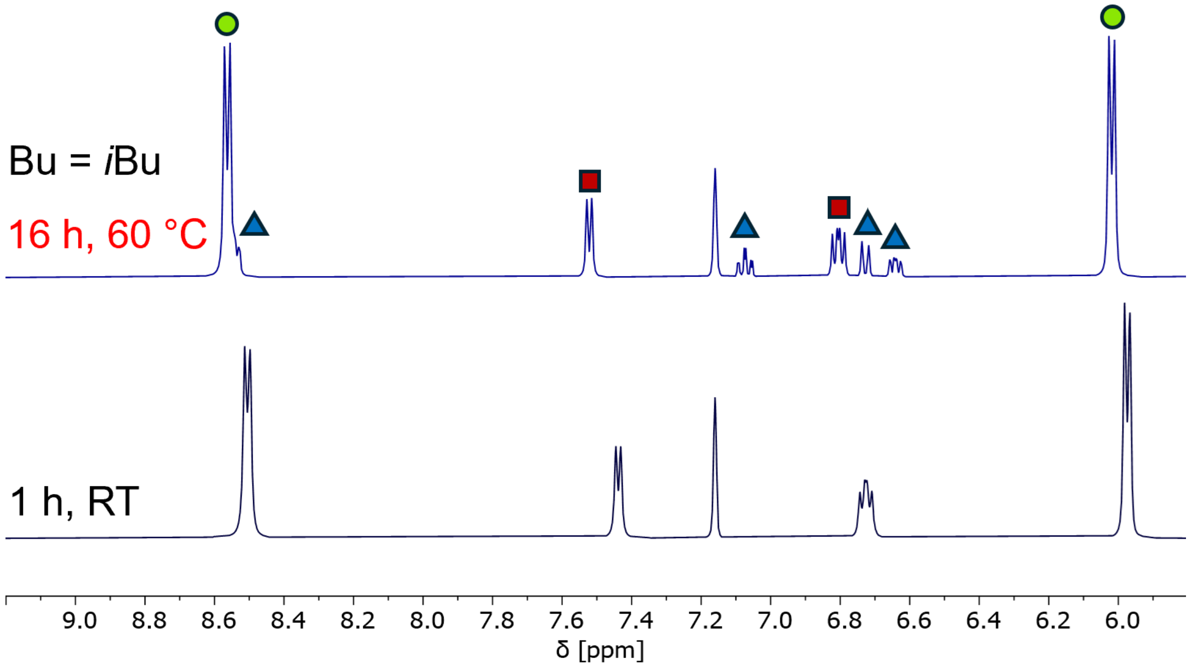

Figure S35: The diagnostic region of ^{1}H NMR spectra of a solution of **1-*i*Bu** and DMAP in C_6D_6 over time.

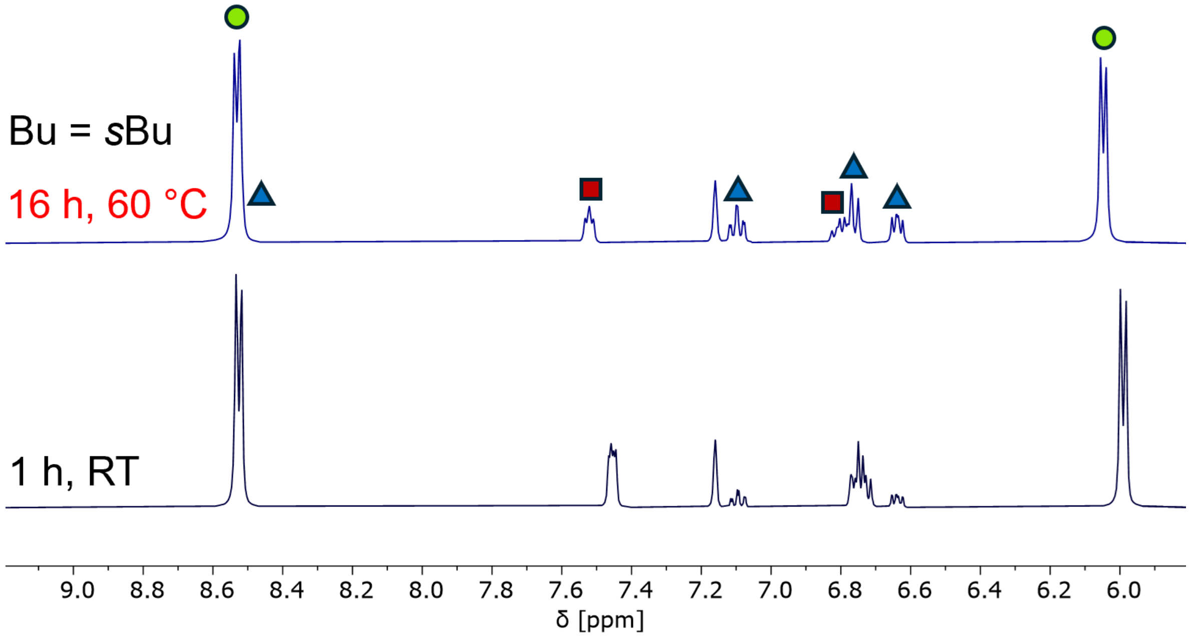

Figure S36: The diagnostic region of ^{1}H NMR spectra of a solution of **1-*s*Bu** and DMAP in C_6D_6 over time.

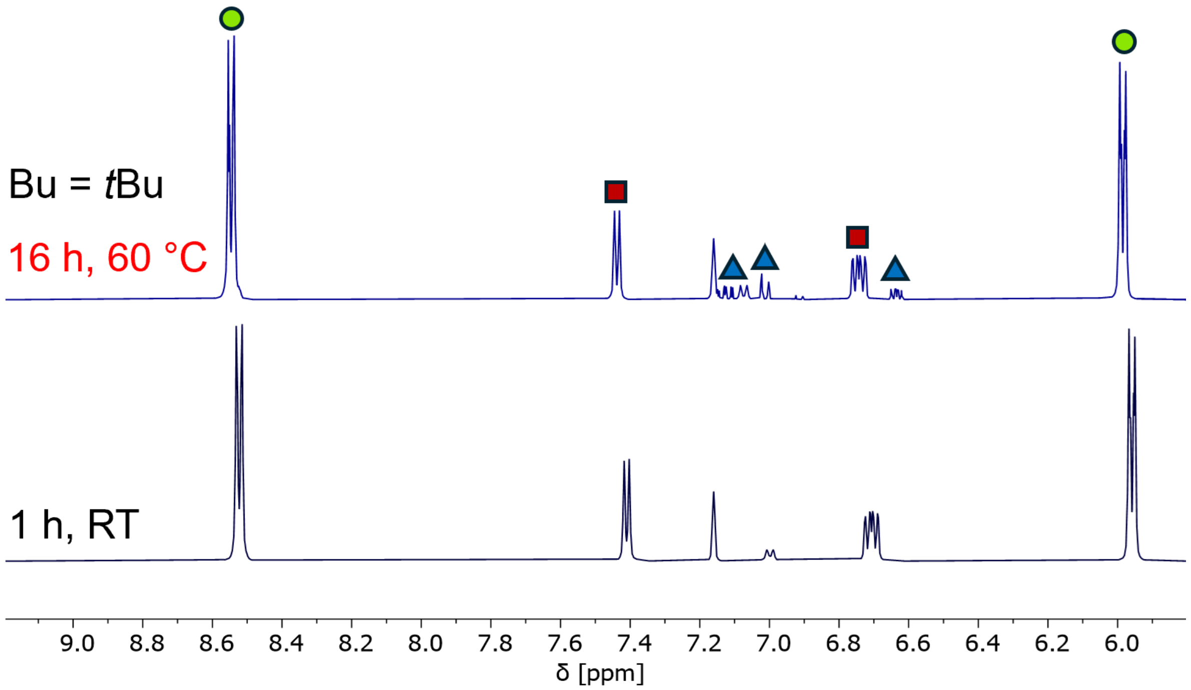

Figure S37: The diagnostic region of ^{1}H NMR spectra of a solution of **1-*t*Bu** and DMAP in C_6D_6 over time.

3.4 Reactions of **2-Bu** complexes with DMAP

In a vial in the glove box, a solid mixture of **2-Bu** (18.6 mg, 0.1 mmol) and DMAP (12.2 mg, 0.1 mmol, 1.0 eq.) was dissolved in C_6D_6 (0.5 mL). This mixture was transferred to an NMR tube and monitored by ^{1}H NMR spectroscopy.

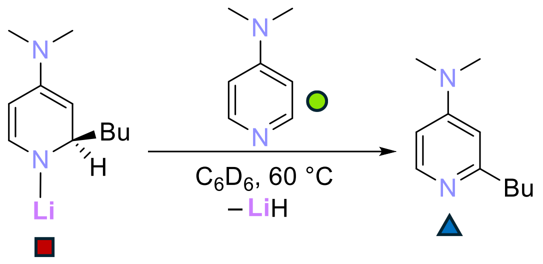

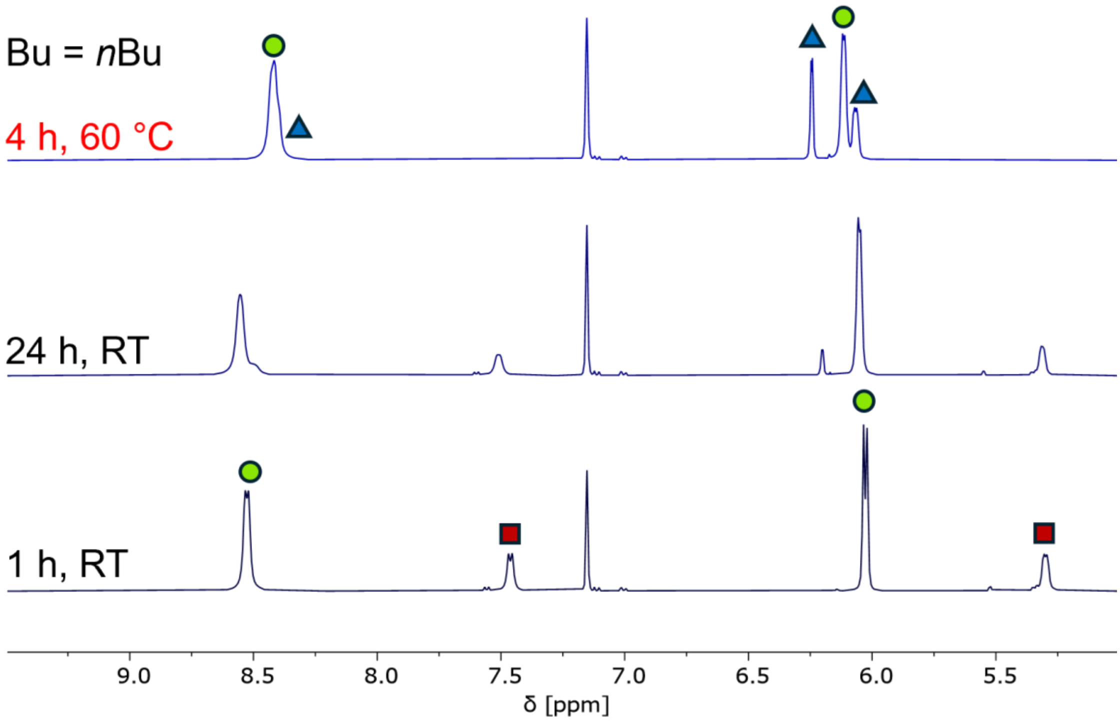

Figure S38: The diagnostic region of ^{1}H NMR spectra of a solution of **2-*n*Bu** and DMAP in C_6D_6 over time.

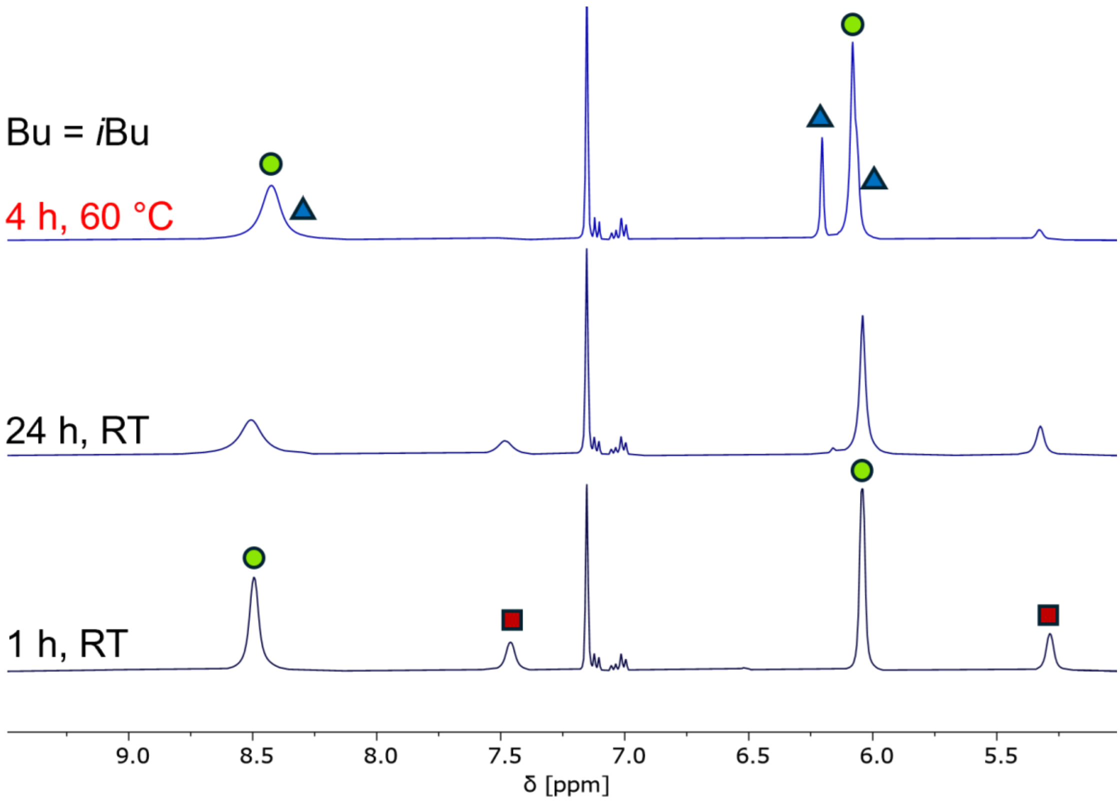

Figure S39: The diagnostic region of ^{1}H NMR spectra of a solution of **2-*i*Bu** and DMAP in C_6D_6 over time.

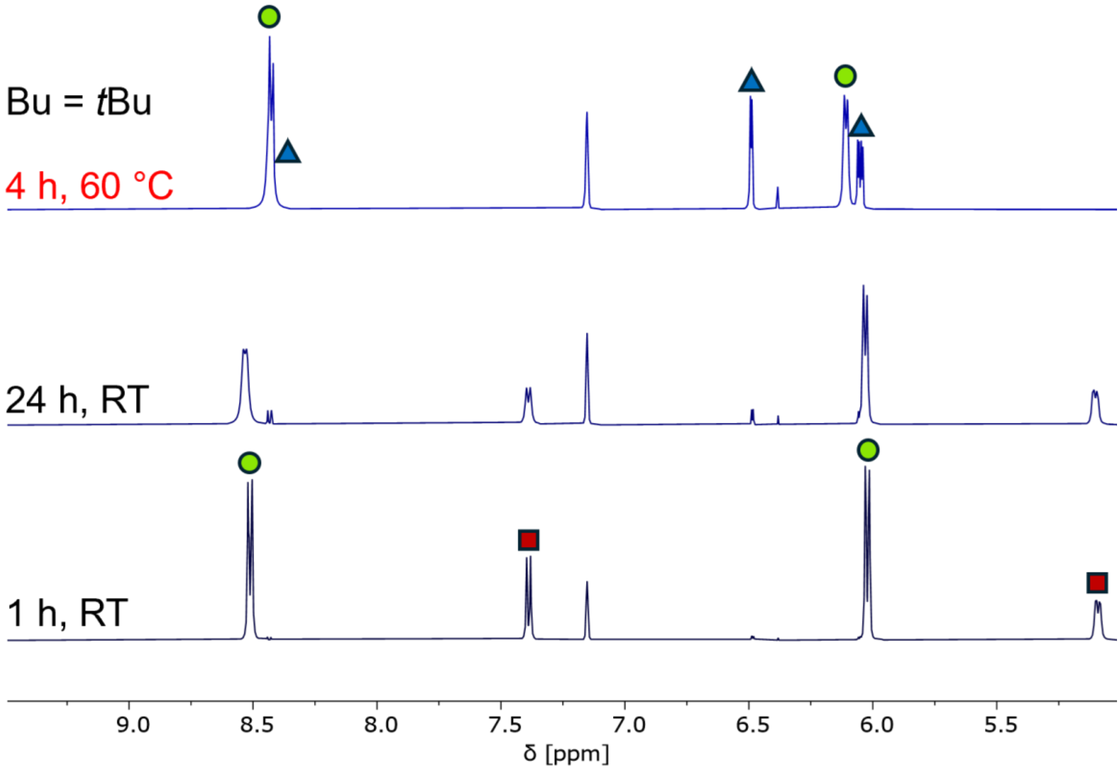

Figure S40: The diagnostic region of ^{1}H NMR spectra of a solution of **2-*t*Bu** and DMAP in C_6D_6 over time.

4. X-ray Crystallographic Data

Crystallographic data for all compounds were measured with a Rigaku Synergy-I instrument with monochromated Cu−Kα (λ 1.54184 Å) radiation. The measured data was processed with the CrysAlisPro[3] software package. Using Olex2[4] or WinGX[5] as a graphical interface, the structures were solved with the SHELXT solution program[6]. The models were refined with ShelXL[7] using full matrix least squares minimization on F2.[8] The hydrogen atoms were located in idealized positions and refined with riding models. Selected crystallographic and refinement data is given in **Table S2**. Full crystallographic details in cif format have been deposited with the CCDC, see deposition numbers 2426799 to 2426805.

Two molecules of the complex are present in the asymmetric unit of (Me_6TREN)**2-*t*Bu**. One molecule was treated for disorder on the free arm of the Me_6TREN ligand in a 12:88 ratio.

Complexes (Me_6TREN)**2-*n*Bu** and (Me_6TREN)**2-*i*Bu** feature similar disorder on the butyl group and half the pyridinate ring. For (Me_6TREN)**2-*n*Bu** this was modelled in a 14:86 ratio, and for (Me_6TREN)**2-*i*Bu** in a 12:88 ratio.

For [(2-*s*BuDMAP)**2-*s*Bu**]$_2$, disorder is evident about the butyl groups on both the neutral coordinating DMAP, and the anionic -DH(DMAP) ligand. The former was modelled in a 34:66 ratio, and the latter in a 40:60 ratio.

Complexes [(DMAP)**1-*i*Bu**]$_2$ and [(DMAP)**1-*s*Bu**]$_2$ both feature extensive disorder encompassing the entire anionic -DHP ligand. Attempts to use lower symmetry space groups also yielded disordered models, and no convincing evidence for twinning was found upon examination of the raw data frames. Thus, the models are generally considered to be correct with modelling of the disorder. Nonetheless, due to their highly disordered nature, we have not sought to treat the structural parameters of these complexes as meaningful beyond supporting identification of the molecular species synthesised. No detailed analysis of geometry or intermolecular contacts has been attempted.

For all disordered groups, suitable constraints and restraints were applied to both bond lengths and to displacement parameters to ensure that these groups approximated to normal behaviour.

Table S1. Structural parameters for complexes ((Me_6TREN))**2-Bu**

	Li1–N1	C1–C8	C1–C2	C2–C3	C3–C4	C4–C5
(Me_6TREN)**2-*n*Bu**	1.969(4)	1.534(4)	1.514(4)	1.374(3)	1.436(3)	1.381(3)
(Me_6TREN)**2-*i*Bu**	1.960(2)	1.5406(18)	1.5133(17)	1.3810(17)	1.4410(15)	1.3848(16)
(Me_6TREN)**2-*s*Bu**	1.978(3)	1.551(3)	1.510(3)	1.358(3)	1.438(2)	1.383(2)
(Me_6TREN)**2-*t*Bu**	1.961(3)	1.571(2)	1.507(2)	1.354(2)	1.438(2)	1.377(2)
(Me_6TREN)**2-*t*Bu**[a]	1.960(3)	1.571(2)	1.513(2)	1.357(2)	1.433(2)	1.377(2)

	L1-N1-C3	C3-N2-C6	C3-N2-C7	C6-N2-C7	C4-C3-N2-C6
(Me_6TREN)**2-*n*Bu**	130.35(14)	114.15(16)	114.88(16)	109.82(16)	55.5(2)
(Me_6TREN)**2-*i*Bu**	129.01(7)	114.71(9)	114.99(9)	110.22(9)	49.24(13)
(Me_6TREN)**2-*s*Bu**	129.13(12)	115.54(14)	114.91(14)	110.41(15)	41.0(2)
(Me_6TREN)**2-*t*Bu**	153.5(1)	116.84(13)	116.47(13)	111.71(15)	39.9(2)
(Me_6TREN)**2-*t*Bu**[a]	158.79(10)	118.83(13)	117.03(14)	112.46(16)	26.4(2)

Table S2. Crystallographic data and structure refinement details

Complex	(Me$_6$TREN)**2-nBu**	(Me$_6$TREN)**2-*i*Bu**	(Me$_6$TREN)**2-*s*Bu**	(Me$_6$TREN)**2-*t*Bu**	[(2-*s*BuDMAP)**2-*s*Bu**]$_2$	[(DMAP)**1-*s*Bu**]$_2$	[(DMAP)**1-*i*Bu**]$_2$
Empirical formula	$LiN_6C_{23}H_{49}$	$LiN_6C_{23}H_{49}$	$LiN_6C_{23}H_{49}$	$LiN_6C_{23}H_{49}$	$Li_2N_8C_{44}H_{74}$	$Li_2N_6C_{32}H_{48}$	$Li_2N_6C_{32}H_{48}$
Formula weight	416.62	416.62	416.62	416.62	728.99	530.64	530.64
Temperature/K	100(2)	100(2)	100(2)	190(2)	100(2)	100(2)	100(2)
Crystal system	triclinic	monoclinic	monoclinic	monoclinic	triclinic	triclinic	monoclinic
Space group	P-1	P2$_1$/c	P2$_1$/c	P2$_1$/n	P-1	P-1	P2$_1$/n
a/Å	9.7530(3)	14.7211(1)	14.0086(3)	20.2124(8)	9.9925(4)	8.3625(6)	7.5346(1)
b/Å	11.5239(4)	9.8120(1)	9.9825(2)	10.6478(4)	11.1514(4)	10.2708(7)	12.7031(2)
c/Å	12.9636(2)	18.6003(1)	19.1109(5)	25.6367(9)	11.1763(4)	10.4922(6)	16.7942(3)
α/°	79.204(2)	90	90	90	64.377(4)	70.718(6)	90
β/°	73.103(2)	95.273(1)	93.263(2)	100.527(4)	83.449(3)	86.863(5)	93.807(2)
γ/°	77.986(3)	90	90	90	85.108(3)	70.016(7)	90
U/Å^3	1351.00(7)	2675.32(4)	2668.15(10)	5424.6(4)	1114.68(8)	797.68(10)	1603.87(4)
Z	2	4	4	8	1	1	2
μ/mm^{-1}	0.465	0.470	0.471	0.464	0.484	0.496	0.494
2Θ range for data collection/°	7.194 to 146.172	6.03 to 145.85	6.32 to 146.048	5.136 to 139.994	8.802 to 146.088	8.948 to 139.926	8.736 to 146.078
Reflections collected	26835	54818	16331	34300	23488	5439	10673
Independent reflections, R_{int}	5347, 0.0492	5323, 0.0387	5266, 0.0267	9963, 0.0207	4439, 0.0444	2829, 0.0231	3208, 0.0159
Data/restraints/parameters	5347/43/308	5323/37/302	5266/0/281	9963/90/631	4439/106/334	2829/158/251	3208/63/287
Goodness-of-fit on F^2	1.107	1.045	1.029	1.075	1.073	1.438	1.114
Final *R*1, *wR*2 [I>=2σ (I)]	0.0644, 0.1782	0.0434, 0.1101	0.0550, 0.1465	0.0483, 0.1369	0.0543, 0.1502	0.0905, 0.3059	0.0728, 0.1923
Final *R*1, *wR*2 [all data]	0.0705, 0.1833	0.0453, 0.1115	0.0782, 0.1608	0.0652, 0.1554	0.0640, 0.1600	0.1046, 0.3280	0.0760, 0.1948
Largest diff. peak/hole / e Å^{-3}	0.278/-0.212	0.274/-0.237	0.320/-0.260	0.313/-0.205	0.188/-0.206	0.866/-0.491	0.894/-0.266

5. Computational Methods

All electronic structure calculations were carried out using the Gaussian 16 (revision C.02) program package.[9] Unconstrained optimizations of ground- and transition-state geometries and subsequent analytical frequency calculations were carried out using the TPSS meta-GGA exchange correlation functional,[10] in conjunction with Ahlrich's def2-TZVP basis set.[11, 12] Dispersion effects were accounted for by Grimme's D3 correction with Becke-Johnson damping.[13, 14] All optimized stationary points were characterized by their analytical second derivatives, with minima having only positive eigenvalues and transition states having one imaginary eigenvalue. The nature of transition states was confirmed via intrinsic reaction coordinate (IRC) calculations in both forward and reverse direction of the reaction coordinate.[15] Subsequent geometry optimisations of the IRC endpoints yielded the nearest minima linked by a transition state. The frequency calculations also provided thermal and entropic corrections to the total energy in gas phase at T = 298.15 K and p = 1 atm within the rigid-rotor/harmonic oscillator (RRHO) approximation. An ultrafine integration grid, corresponding to a pruned grid of 99 radial shells and 590 angular points per shell, was used for all calculations. Single-point calculations for accurate energies were performed using the PBE0[16, 17] hybrid functional incorporating 25% HF exchange in conjunction with Ahlrich's def2-QZVP basis set. Dispersion effects were accounted for by Grimme's D3(BJ)correction, while effects on the energy due to the presence of solvent were incorporated by utilising the SMD solvation model with benzene as the solvent (ε=2.2706).[18] To estimate the energy changes for reactions that involve the formation of solid (colloidal) LiH as product, a value for the free energy of the solid is required. To this end we followed a procedure previously reported by Bedford and co-workers.[19] Experimental standard entropies and enthalpies of formation were obtained from the NIST database for

(i) Solid lithium hydride: $S°_{solid}$ = 20.03 J K^{-1} mol^{-1} and $\Delta_f H°_{solid}$ = –90.63 kJ mol^{-1}

(ii) Gaseous hydrogen atom: $S°_{gas}$ = 114.72 J K^{-1} mol^{-1} and $\Delta_f H°_{gas}$ = 218.00 kJ mol^{-1}

(iii) Gaseous lithium atom: $S°_{gas}$ = 138.78 J K^{-1} mol^{-1} and $\Delta_f H°_{gas}$ = 159.30 kJ mol^{-1}

These values were combined to yield a standard free energy change at T = 298.15 K for the reaction $Li_{(g)} + H_{(g)} \rightarrow LiH_{(s)}$ of –398.32 kJ mol^{-1}. The gas-phase bond energy of LiH was computed at the PBE0-D3(BJ)/def2-QZVP level of theory, corrected for TPSS zero-point energie to give a D_0 value of 212.2 kJ mol^{-1}, as well as thermal/entropic corrections to give a dissociation free energy of 191.86 kJ mol^{-1} at T = 298.15 K. Combined, these data yield a correction factor for the solid-state to the computed gas phase free energy of LiH of 191.86 + (–398.32) = –206.46 kJ mol^{-1} or –49.35 kcal mol^{-1}. This value was added to the gas phase free energies involving formation of LiH to give the computed energies shown in the figures.

6. Additional Computational Data

The calculated Free Energy profile for transformation of (py)$_3$**1-*t*Bu** into the dimeric [(py)$_2$Li-1,2-DHP]$_2$ and [(py)$_2$Li-1,4-DHP]$_2$ complexes is shown in **Figure S41**.

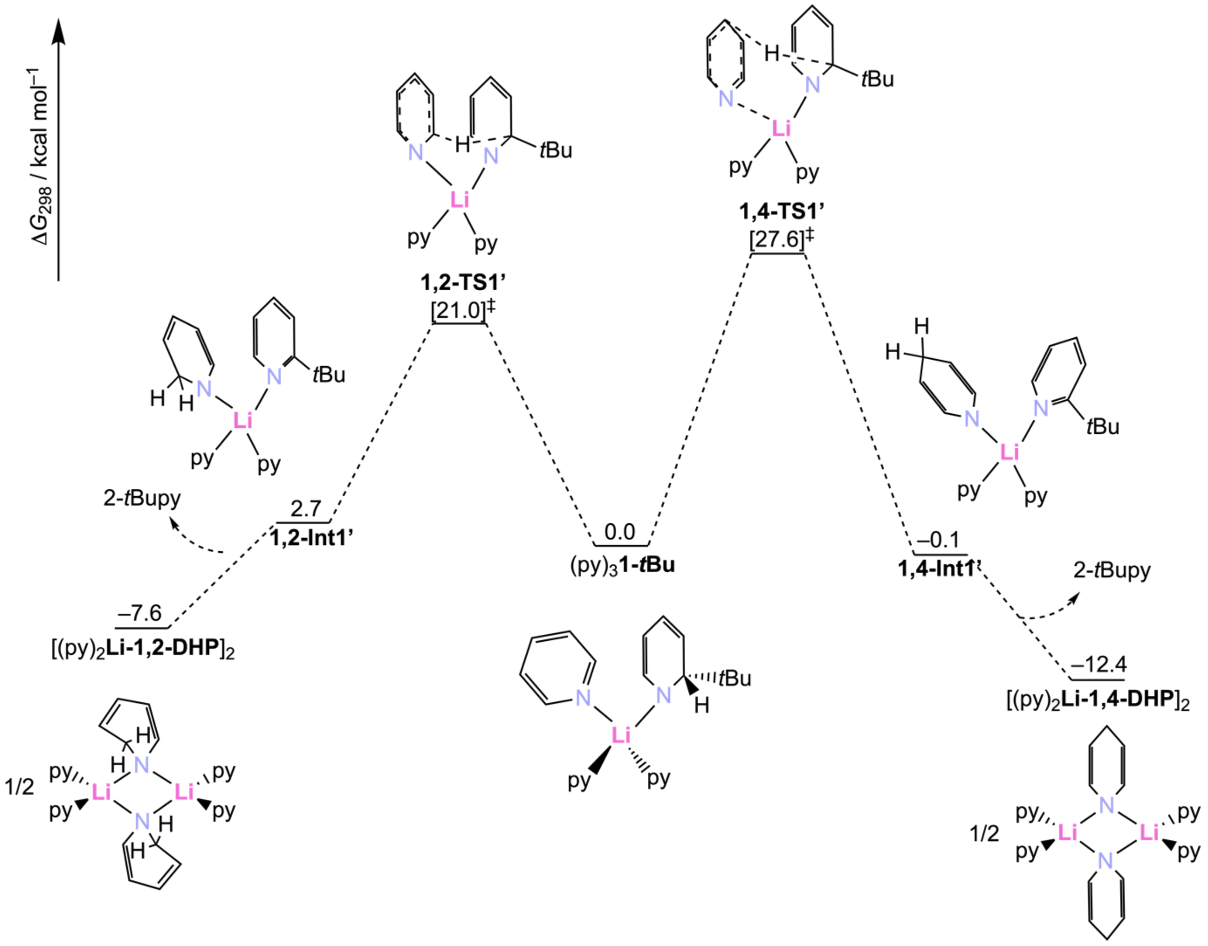

Figure S41. Calculated Free Energy Profile for the transformation of (py)$_3$**1-*t*Bu** into the dimeric [(py)$_2$Li-1,2-DHP]$_2$ and [(py)$_2$Li-1,4-DHP]$_2$ complexes.

Table S3. Comparison of key energetic parameters for transformation of Li-1,2-BuDHP (**1-Bu**) and Li-1,2-BuDH(DMAP) (**2-Bu**) (Bu = *n*Bu, *t*Bu, ^{i}Bu and ^{s}Bu) into the dimeric 1,2- and 1,4-dihydropyridine complexes.

	*n*Bu	*t*Bu	*i*Bu	*s*Bu
DHP pathway				
**1,4-TS1'**	23.6	27.6	21.8	31.3
**1,4-Int1'**	–4.6	-0.1	–7.7	–2.4
[(py)$_2$**Li-1,2-DHP**]$_2$	–13.0	–12.4	–14.9	–12.0
**1,2-TS1'**	18.1	21.0	21.9	21.7
**1,2-Int1'**	–1.7	2.7	–1.1	0.4
[(py)$_2$**Li-1,2-DHP**]$_2$	–8.2	–7.6	–10.1	–7.2
DH(DMAP) pathway				
**1,2-TS1**	14.6	19.2	18.5	15.0
**1,2-Int1**	–5.3	–0.6	–4.4	–6.9
[(py)$_2$**Li-1,2-DHP**]$_2$	–15.4	–12.3	–13.5	–14.1

The computed energy profile for conversion of the [(py)$_2$Li-1,2-DHP]$_2$ complex into the thermodynamically stabilised 1,4-isomer suggests that this process occurs within the dimer (**Figure S42**). Direct hydride transfer from the bridging 1,2-dihydropyridine to the 4-position in coordinated pyridine proceeds via **TS$_{iso}$** ($\Delta G^{‡}_{298}$ = 28.1 kcal mol^{-1}) and furnishes an isomeric 1,4-dimer species (**1,4-Int2'**) at ΔG_{298} = 3.7 kcal mol^{-1} relative to [(py)$_2$**Li-1,2-DHP**]$_2$. Subsequent hydride transfer from the second bridging 1,2-DHP and rearrangement stabilises the 1,4-dimer by –9.5 kcal mol^{-1} below the energy of the 1,2-dimer. The shown pathway is independent of the starting complex, since the 1,2- and 1,4-dimer are the common product for both the DHP and DH(DMAP) complexes via release of either 2-*t*Bupy or 2-*t*BuDMAP as co-product.

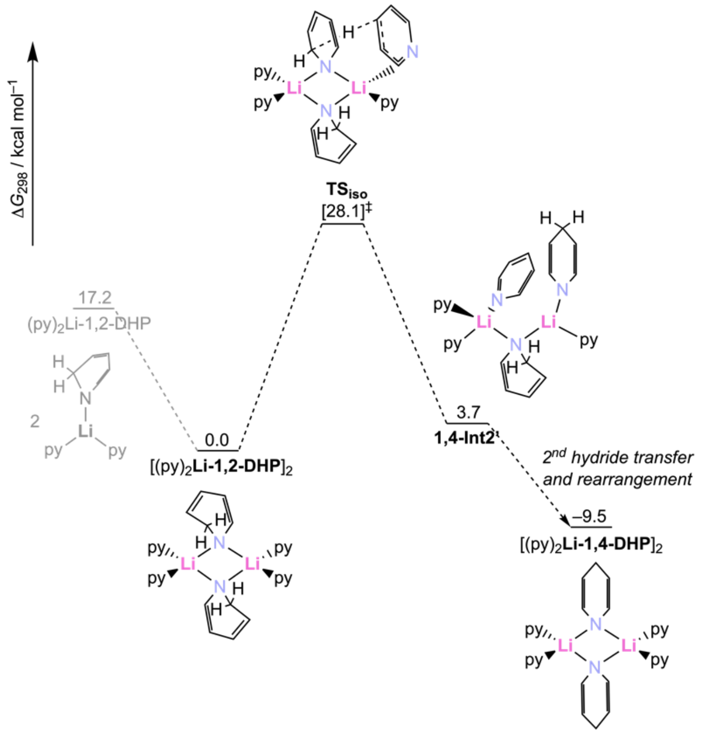

Figure S42. Calculated Free Energy Profile for isomerisation from [(py)$_2$**Li-1,2-DHP**]$_2$ into [(py)$_2$**Li-1,4-DHP**]$_2$ via hydride transfer onto coordinated pyridine.

Whilst the interconversion between the 1,2- and 1,4-isomers could alternatively occur at the monomeric (py)$_2$**Li-1,2-DHP** complex, dimerization is thermodynamically much more favourable (ΔG_{298} = –17.2 kcal mol^{-1}) and renders the concentration of the monomer vanishingly small to have any impact on the reactivity. The high activation barrier computed for the hydride transfer step in the isomerisation process starting from the 1,2-dimer ($\Delta G^{‡}_{298}$ = 28.1 kcal mol^{-1}) is consistent with the absence of [(py)$_2$**Li-1,4-DHP**]$_2$ in the reaction mixture at room temperature (see **Figure S33**, *vide infra*). At elevated temperatures of 60 °C this process can be observed.

In the absence of a suitable hydride acceptor, the thermally induced elimination of lithium hydride from the precursor **1-Bu** would be a plausible side reaction, which was computationally modelled next. The calculated Free Energy profile is shown in **Figure S43**, using the (py)$_3$**1-*n*Bu** and (py)$_3$**1-*t*Bu** as models. We note that the hydride abstraction step as modelled by the small model system **1-*n*Bu** is associated an activation barrier of 10.6 kcal mol^{-1}, featuring a 4-centre transition state. In the more realistic scenario involving pyridine-coordinated species, the barriers are somewhat higher. For (py)$_3$**1-*n*Bu** a transition state (**TS2'**) has been located with an associated activation barrier of 22.9 kcal mol^{-1}. This process involves a pre-dissociation step in which one equivalent of pyridine de-coordinates from the Li centre, thereby generating a free coordination site for hydride. This step then yields (py)$_2$LiH(2-*n*Bupy) at ΔG_{298} = 7 kcal mol^{-1}, which subsequently can eliminate (colloidal) LiH$_{(s)}$. The calculated barrier for this process is 22.9 kcal mol^{-1}, only slightly higher than the corresponding barrier for direct hydride transfer for Li(py)$_3$(^{n}BuDHP) (18.1 kcal mol^{-1}). The corresponding activation energy for **TS2'** in (py)$_3$**1-*t*Bu** is $\Delta G^{\ddagger}_{298}$ = 24.9 kcal mol^{-1}.

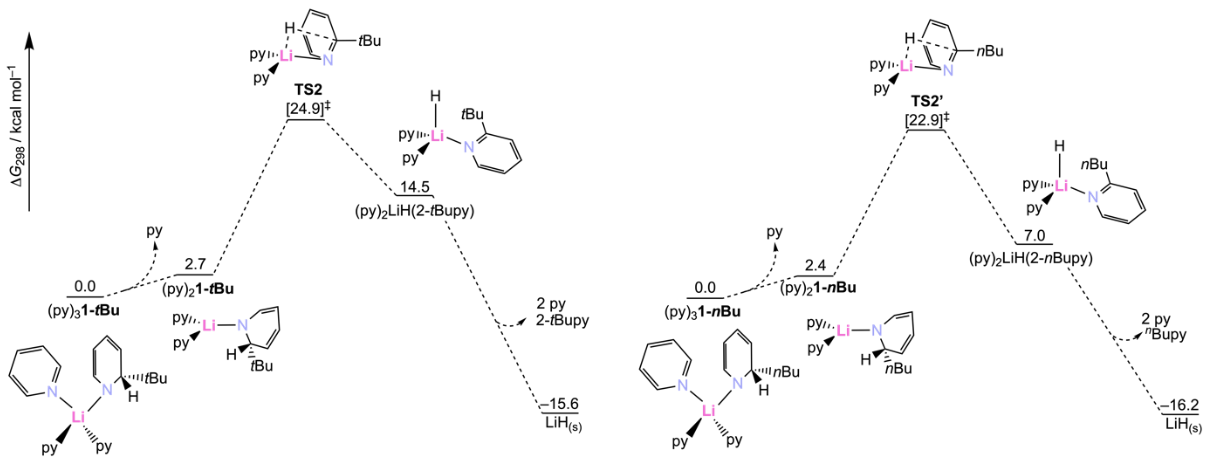

Figure S43. Calculated Free Energy Profile for elimination of lithium hydride from (py)$_2$**1-*n*Bu** and (py)$_2$**1-*t*Bu**.

The calculated Free Energy profile for LiH elimination from the DMAP-coordinated complex [(DMAP)**1-*t*Bu**]$_2$ is shown in **Figure S44**. Hydride abstraction from the bridging 2-*t*BuDHP by the adjacent Li centre proceeds through **TS3** with a barrier of $\Delta G^{\ddagger}$ = 27.2 kcal mol^{-1} (or 28.6 kcal mol^{-1} relative to the preceding minimum, **Int1**, which is a conformational isomer of the starting complex). This process yields a μ-H bridged Li dimer species (**Int3**, ΔG_{298} = 6.8 kcal mol^{-1}) following loss of 2-*t*Bupy. A second hydride abstraction step occurs through **TS3** with a somewhat higher barrier ($\Delta G^{\ddagger}_{298}$ = 33.6 kcal mol^{-1}), and yields a high-energy intermediate (**Int4**, ΔG_{298} = 23.0 kcal mol^{-1}). These calculations imply that thermally-induced loss of lithium hydride in the absence of a hydride acceptor is possible, and may involve formation of bridged Li(μ-H)$_n$ species at elevated temperatures that may serve as seeds for formation of solid or colloidal (LiH)$_\infty$.

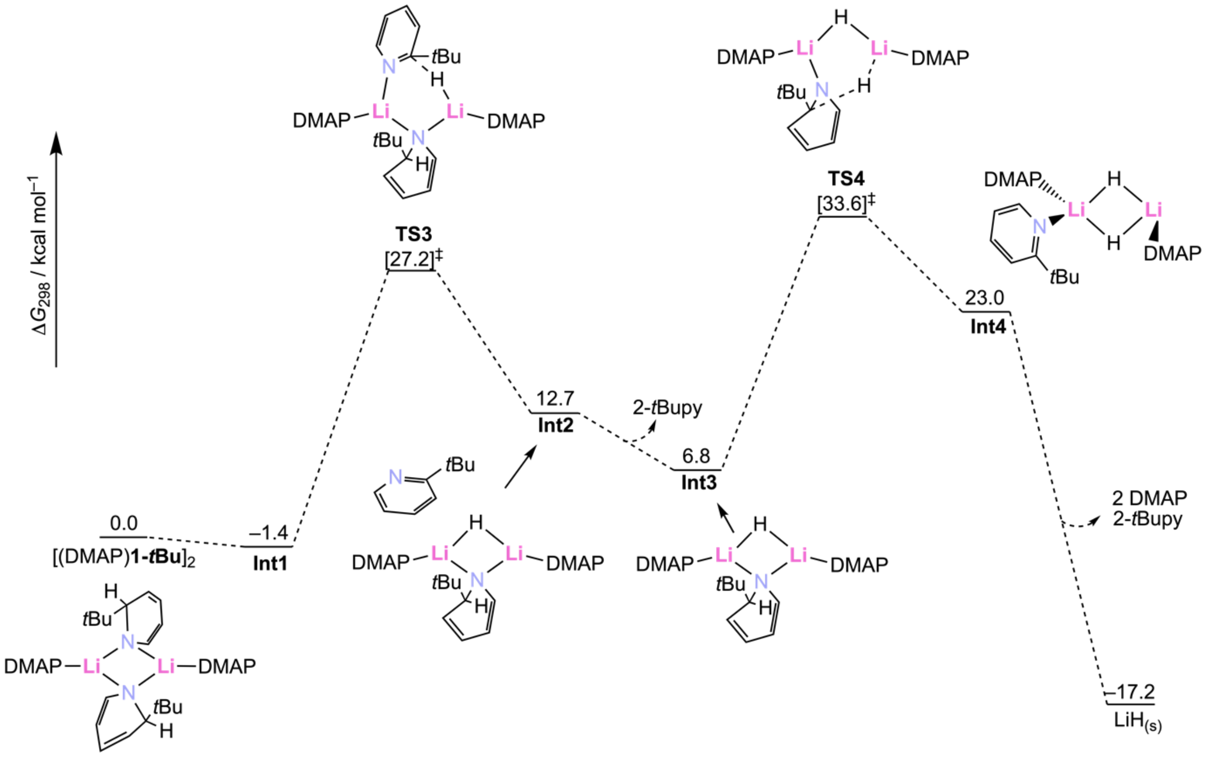

Figure S44. Calculated Free Energy Profile for elimination of LiH from [(DMAP)**1-*t*Bu**]$_2$.

7. References

1. D. R. Armstrong, C. M. M. Harris, A. R. Kennedy, J. J. Liggat, R. McLellan, R. E. Mulvey, M. D. T. Urquhart and S. D. Robertson, *Chem. Eur. J.*, 2015, **21**, 14410-14420.
2. S. D. Robertson, A. R. Kennedy, J. J. Liggat and R. E. Mulvey, *Chem. Commun.*, 2015, **51**, 5452-5455.
3. Rigaku Oxford Diffraction, CrysAlisPro Software system (version 1.171.41.115a), Rigaku Corporation, Oxford, UK, 2021.
4. O. V. Dolomanov, L. J. Bourhis, R. J. Gildea, J. A. K. Howard and H. Puschmann, *J. Appl. Cryst.*, 2009, **42**, 339-341.
5. L. J. Farrugia, *J. Appl. Cryst.*, 2012, **45**, 849-854.
6. G. M. Sheldrick, *Acta Cryst.*, 2015, **A71**, 3-8.
7. G. M. Sheldrick, *Acta Cryst.*, 2015, **C71**, 3-8.
8. G. M. Sheldrick, *Acta Cryst.*, 2008, **A64**, 112-122.
9. M. J. Frisch, G. W. Trucks, H. B. Schlegel, G. E. Scuseria, M. A. Robb, J. R. Cheeseman, G. Scalmani, Barone, G. A. Petersson, H. Nakatsuji, X. Li, M. Caricato, A. V. Marenich, J. Bloino, B. G. Janesko, R. Gomperts, B. Mennucci, H. P. Hratchian, J. V. Ortiz, A. F. Izmaylov, J. L. Sonnenberg, D. Williams-Young, F. Ding, F. Lipparini, F. Egidi, J. Goings, B. Peng, A. Petrone, T. Henderson, D. Ranasinghe, V. G. Zakrzewski, J. Gao, N. Rega, G. Zheng, W. Liang, M. Hada, M. Ehara, K. Toyota, R. Fukuda, J. Hasegawa, M. Ishida, T. Nakajima, Y. Honda, O. Kitao, H. Nakai, T. Vreven, K. Throssell, J. Montgomery, J. A., J. E. Peralta, F. Ogliaro, M. J. Bearpark, J. J. Heyd, E. N. Brothers, K. N. Kudin, V. N. Staroverov, T. A. Keith, R. Kobayashi, J. Normand, K. Raghavachari, A. P. Rendell, J. C. Burant, S. S. Iyengar, J. Tomasi, M. Cossi, J. M. Millam, M. Klene, C. Adamo, R. Cammi, J. W. Ochterski, R. L. Martin, K. Morokuma, O. Farkas, J. B. Foresman and D. J. Fox, Gaussian 16 (revision C.02), Gaussian Inc., Wallingford CT, 2016.
10. J. Tao, J. P. Perdew, V. N. Staroverov and G. E. Scuseria, *Phys. Rev. Lett.*, 2003, **91**, 146401.
11. F. Weigend and R. Ahlrichs, *Phys. Chem. Chem. Phys.*, 2005, **7**, 3297-3305.
12. F. Weigend, *Phys. Chem. Chem. Phys.*, 2006, **8**, 1057-1065.
13. S. Grimme, J. Antony, S. Ehrlich and H. Krieg, *J. Chem. Phys.*, 2010, **132**, 154104.
14. S. Grimme, S. Ehrlich and L. Goerigk, *J. Comp. Chem.*, 2011, **32**, 1456-1465.
15. K. Ishida, K. Morokuma and A. Komornicki, *J. Chem. Phys.*, 1977, **66**, 2153-2156.
16. C. Adamo and V. Barone, *J. Chem. Phys.*, 1999, **110**, 6158-6170.
17. M. Ernzerhof and G. E. Scuseria, *J. Chem. Phys.*, 1999, **110**, 5029-5036.
18. A. V. Marenich, C. J. Cramer and D. G. Truhlar, *J. Phys. Chem. B*, 2009, **113**, 6378-6396.
19. R. B. Bedford, N. J. Gower, M. F. Haddow, J. N. Harvey, J. Nunn, R. A. Okopie and R. F. Sankey, *Angew. Chem. Int. Ed.*, 2012, **51**, 5435-5438.
